# Flow cytometry sorting of nuclei enables the first global characterization of *Paramecium* germline DNA and transposable elements

**DOI:** 10.1186/s12864-017-3713-7

**Published:** 2017-04-26

**Authors:** Frédéric Guérin, Olivier Arnaiz, Nicole Boggetto, Cyril Denby Wilkes, Eric Meyer, Linda Sperling, Sandra Duharcourt

**Affiliations:** 1 Institut Jacques Monod, CNRS, UMR 7592, Université Paris Diderot, Sorbonne Paris Cité, Paris, F-75205 France; 2 Institute of Integrative Biology of the Cell, UMR9198 CNRS CEA Univ, Paris-Sud Université Paris-Saclay, 91198 Gif-sur-Yvette, France; 3 Current address: Institut de Biologie et de Technologies de Saclay (IBITECS), CEA, F-91191 Gif-sur-Yvette Cedex, France; 4 IBENS, Département de Biologie, Ecole Normale Supérieure, CNRS, Inserm, PSL Research University, F-75005 Paris, France

**Keywords:** Flow cytometry, Non-LTR retrotransposons, ITm DNA transposons, Programmed DNA elimination, High throughput sequencing

## Abstract

**Background:**

DNA elimination is developmentally programmed in a wide variety of eukaryotes, including unicellular ciliates, and leads to the generation of distinct germline and somatic genomes. The ciliate *Paramecium tetraurelia* harbors two types of nuclei with different functions and genome structures. The transcriptionally inactive micronucleus contains the complete germline genome, while the somatic macronucleus contains a reduced genome streamlined for gene expression. During development of the somatic macronucleus, the germline genome undergoes massive and reproducible DNA elimination events. Availability of both the somatic and germline genomes is essential to examine the genome changes that occur during programmed DNA elimination and ultimately decipher the mechanisms underlying the specific removal of germline-limited sequences.

**Results:**

We developed a novel experimental approach that uses flow cell imaging and flow cytometry to sort subpopulations of nuclei to high purity. We sorted vegetative micronuclei and macronuclei during development of *P. tetraurelia*. We validated the method by flow cell imaging and by high throughput DNA sequencing. Our work establishes the proof of principle that developing somatic macronuclei can be sorted from a complex biological sample to high purity based on their size, shape and DNA content. This method enabled us to sequence, for the first time, the germline DNA from pure micronuclei and to identify novel transposable elements. Sequencing the germline DNA confirms that the Pgm domesticated transposase is required for the excision of all ~45,000 Internal Eliminated Sequences. Comparison of the germline DNA and unrearranged DNA obtained from *PGM*-silenced cells reveals that the latter does not provide a faithful representation of the germline genome.

**Conclusions:**

We developed a flow cytometry-based method to purify *P. tetraurelia* nuclei to high purity and provided quality control with flow cell imaging and high throughput DNA sequencing. We identified 61 germline transposable elements including the first *Paramecium* retrotransposons. This approach paves the way to sequence the germline genomes of *P. aurelia* sibling species for future comparative genomic studies.

**Electronic supplementary material:**

The online version of this article (doi:10.1186/s12864-017-3713-7) contains supplementary material, which is available to authorized users.

## Background

**Fig. 1:**
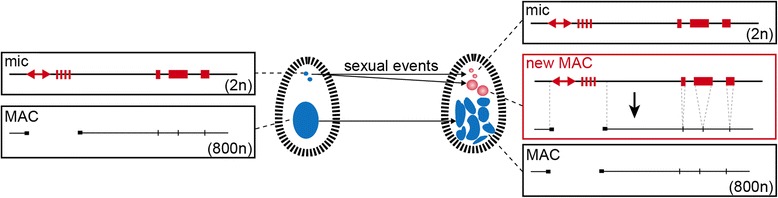
Nuclear dimorphism and programmed DNA elimination in *Paramecium tetraurelia. Left panel*. Each cell contains two distinct types of nuclei: two diploid germline micronuclei (MIC, 2n) and one highly polyploid somatic macronucleus (MAC, 800n). Both nuclei develop from copies of the zygotic nucleus after fertilization. *Right panel*. Massive and reproducible elimination of germline DNA occurs during macronuclear development. Imprecise elimination of germline DNA containing repetitive sequences such as minisatellites (*hatched rectangle*) and transposable elements (*double*-*headed arrow*) is associated with the fragmentation of germline chromosomes into shorter macronuclear molecules healed by *de novo* telomere addition (*black rectangles*). In addition, 45,000 short, non-coding Internal Eliminated Sequences (IESs) (*red rectangles*) scattered throughout the germline genome are precisely excised from coding and intergenic sequences, restoring open reading frames and allowing gene expression

Major genome changes can occur during somatic differentiation. In diverse organisms, programmed DNA elimination leads to the removal of specific-germline DNA sequences during development of somatic cells and thus generates germline and somatic genomes with distinct architectures. This process has been described in a wide variety of animals and in ciliates, suggesting that it has likely arisen independently in different lineages [1]. Ciliates are unicellular eukaryotes with separate germline and somatic nuclei. In the ciliate *Paramecium tetraurelia*, two small, genetically identical diploid micronuclei (MIC, 2n, ~ 3 μm) contain the germline genome that is transmitted to sexual progeny after meiosis. A large, transcriptionally active somatic macronucleus (MAC, 800n, ~ 30 μm) contains a reduced genome streamlined for gene expression. At each sexual cycle, the parental MAC is lost, while new MICs and MACs, destined for the progeny, develop from a copy of the diploid zygotic nucleus. In the new developing MAC, the germline genome is endoreplicated to reach its final ploidy of ~ 800n and undergoes massive programmed DNA elimination (for review [2]) (Fig. 1). Large DNA regions containing transposable elements and other repeated sequences are eliminated, leading to chromosome breakage and *de novo* telomere addition. In addition, ~ 45,000 short, unique, Internal Eliminated Sequences (IESs) are precisely excised. At least 25% of the ~ 100 Mb MIC genome is removed [3]. The distinctive genome architectures of ciliates make them attractive model systems to study the complex mechanisms underlying programmed DNA elimination. Meiosis-specific small RNA and chromatin modification pathways, similar to those found in plants and animals for the formation of heterochromatin and silencing of repeated sequences, are involved in the epigenetic programming of DNA elimination [4, 5].

Comprehensive description of genome changes that occur during programmed DNA elimination requires comparison of the germline and the somatic genomes. While the rearranged somatic MAC genome was sequenced and assembled 10 years ago allowing gene annotation [6], technical difficulties in obtaining pure MIC DNA (0.5% of total genomic DNA) have long been a major obstacle to sequencing the germline genome of *P. tetraurelia*. Pioneering work used Percoll gradient centrifugation to separate MICs from MACs [7]. Despite high bacterial contamination of the resultant MIC DNA [3], this led to the discovery of germline-limited sequences [7, 8]. More recently, DNA enriched in un-rearranged germline-like sequences was obtained from cells RNAi-depleted of PiggyMac (Pgm), the domesticated transposase required for developmental genome rearrangements [9]. Deep-sequencing of this DNA (PGM DNA) enabled genome wide-characterization of 45,000 IESs in *P. tetraurelia* [3]. However, how faithfully PGM DNA mimics the true germline genome found in the MIC remains an open question.

We report here a new and reliable method to purify MICs involving a critical step of flow cytometry. The method also allows isolation of developing MACs. Complete separation of nuclei was validated by flow cell imaging and by high throughput DNA sequencing. We show that PGM DNA is in fact not equivalent to MIC DNA. Contigs assembled from the MIC DNA allowed discovery of new *P. tetraurelia* transposable element families.

## Results and Discussion

### Purification of new developing MACs

**Fig. 2:**
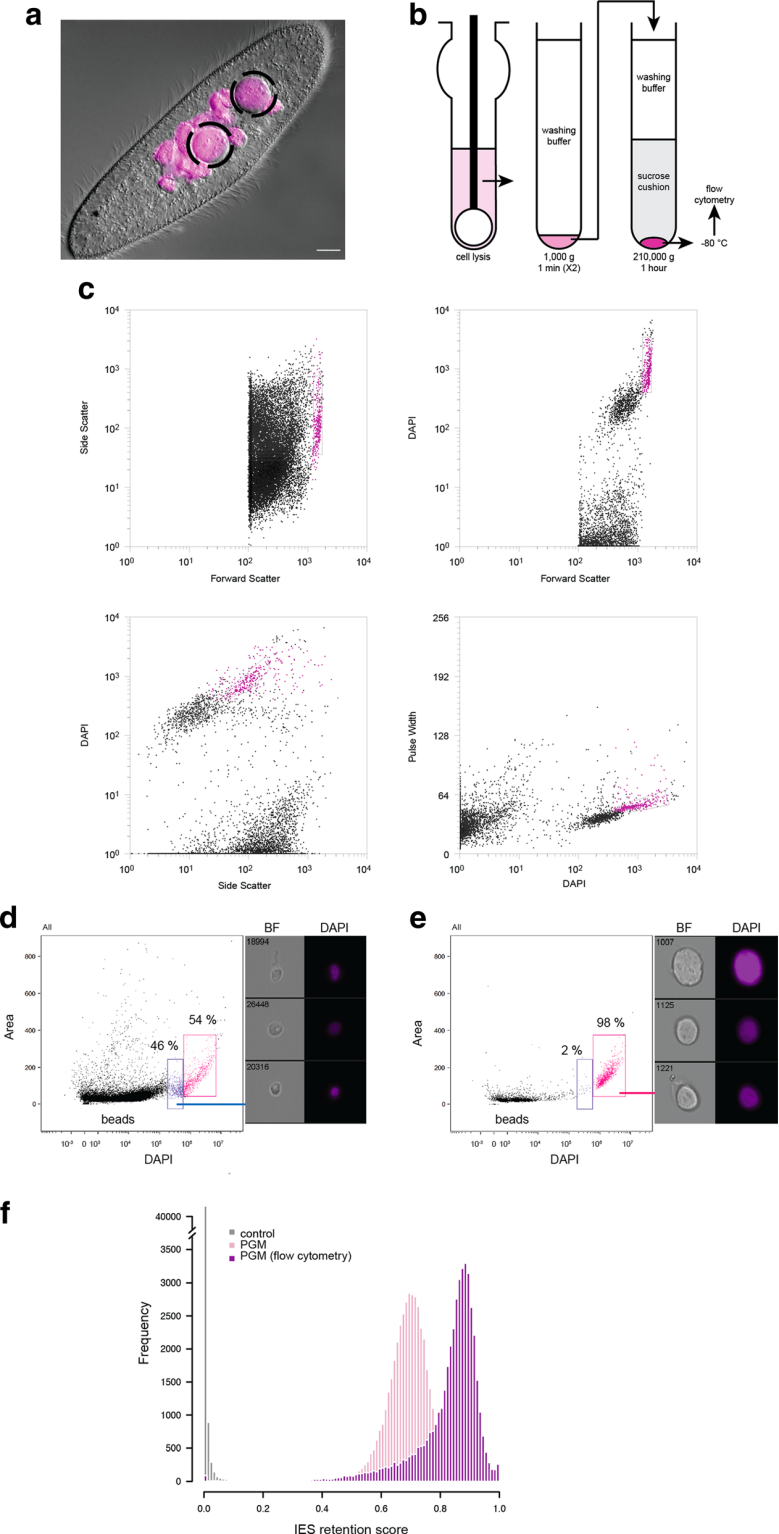
Purification of new developing MACs from *Paramecium tetraurelia* by flow cytometry and validation by flow cell imaging and high throughout DNA sequencing. **a**. DAPI staining of a cell upon *PGM* RNAi at a late developmental stage of the sexual process of autogamy (self-fertilization) is shown on the picture: the two large new developing MACs (*dotted circle*) and the small fragments of the maternal MAC are detected. The scale bar is 10 microns. **b**. Following gentle lysis and cell fractionation, the nuclei preparation is submitted to flow cytometry after staining with DAPI. **c**. Multi-gating flow cytometry strategy used for sorting. Sorting is based on size, granularity and DAPI staining signal of the new developing MACs. An empiric iterative procedure coupled with flow imaging allowed discrimination between developing MACs and fragments, identification of the population of interest, and optimization of the sorting strategy. **d** - **e**. The Amnis ImageStream^X^ imaging flow cytometer is used for quality control. Distribution of DAPI intensity is shown for each event in the sample before (**d**) and after sorting (**e**), respectively. Representative images are displayed in BF (bright field) and DAPI. Objective ×60. **f. **Validation of the sorting strategy by high-throughput DNA sequencing. Histograms of IES retention scores are shown for control (no RNAi), *PGM* RNAi (no sorting) and *PGM* RNAi after sorting (flow cytometry)

Before tackling the purification of the tiny MICs, we decided to purify new developing MACs from cells undergoing the sexual process of autogamy (self-fertilization) (Fig. 1). At each sexual cycle, the parental MAC disintegrates into about 30 small pieces that persist in the cytoplasm, while new MICs and MACs, destined for the progeny, develop from a copy of the diploid zygotic nucleus. Thus, new developing MACs coexist with the two MICs and about 30 small fragments of the maternal MAC (Fig. 2a). We used a published procedure to fractionate the nuclei of Pgm-depleted cells [3] (Fig. 2b). Briefly, nuclei from lysed cells were separated from contaminating organelles and cell debris on a sucrose cushion. The nuclear fraction, containing a mixture of different types of nuclei, was then submitted to flow cytometry (Additional file 1: Figure S1).

A fully developed MAC has a ploidy of 800n [10]. Therefore, new MACs at an advanced developmental stage emit a more intense DAPI (DNA staining) signal than the other nuclei present in the cell at the same stage (MICs and fragments of the maternal MAC). They are also considerably larger than the other nuclei, to accommodate this large amount of chromatin, and are spherical in shape (Fig. 2a). Taking advantage of these characteristics, we FACS-sorted new MACs (~15 μm) according to size (Forward-scattered light, FSC), granularity (Side-scattered light, SSC), pulse width and DAPI signal (Fig. 2c). Purity was measured by flow cell imaging before and after sorting. The developing MAC fraction, that represented 54% of the total nuclear sample before sorting, was enriched to 98% after sorting (Fig. 2d-e). Thus, the sorting procedure conferred considerable improvement over the pre-existing protocol.

To further validate the sorting procedure, we performed high throughput Illumina sequencing of DNA extracted from 266,000 sorted developing MACs (“sorted PGM DNA”) (Additional file 2: Table S1). To identify the IESs in a sequencing sample, we used our previously published pipeline [11]. A total of 44,947 IESs was identified in the sorted PGM DNA, compared to 44,928 IESs in unsorted PGM DNA [3]. The fact that 97% (*n* = 43,839) of the IESs identified in the sorted PGM DNA correspond to the same IESs identified in unsorted PGM DNA testifies to the reliability of our procedure. The 3% difference lies within the estimated error rate of the method [3, 11].

We then quantified the enrichment of our samples in un-rearranged sequences, by calculating a retention score for each of the 44,928 IES sequences present in the previously published *P. tetraurelia* IES reference set [3]. Retention score values range from 0 for no IES retention to 1 for complete IES retention, when the IES is retained in all sequenced copies of the genomic locus in question. As expected (Fig. 2f), retention score distribution in the rearranged MAC DNA control sample is close to 0 (mean 0.005), whereas a Gaussian distribution is observed for the unsorted non-rearranged PGM DNA, with a mean retention score of 0.69. Even if the Pgm endonuclease is required for all IES excision events, the mean retention score of this sample can never reach 1, because the un-rearranged DNA from the developing new MACs is present in the unsorted sample alongside rearranged DNA from the fragments of the maternal MAC. By contrast, the sorted PGM DNA gave a Gaussian distribution with a mean retention score of 0.82. This higher retention score, obtained from the same starting material, reflects greater enrichment in un-rearranged DNA, and thus in developing nuclei, providing further validation for the superiority of the sorting procedure over the existing protocol. In conclusion, this experiment establishes the proof of principle that nuclei can be sorted from a complex biological sample to high purity based on their size, shape and DNA content.

### Purification of MICs from vegetative cells

**Fig. 3:**
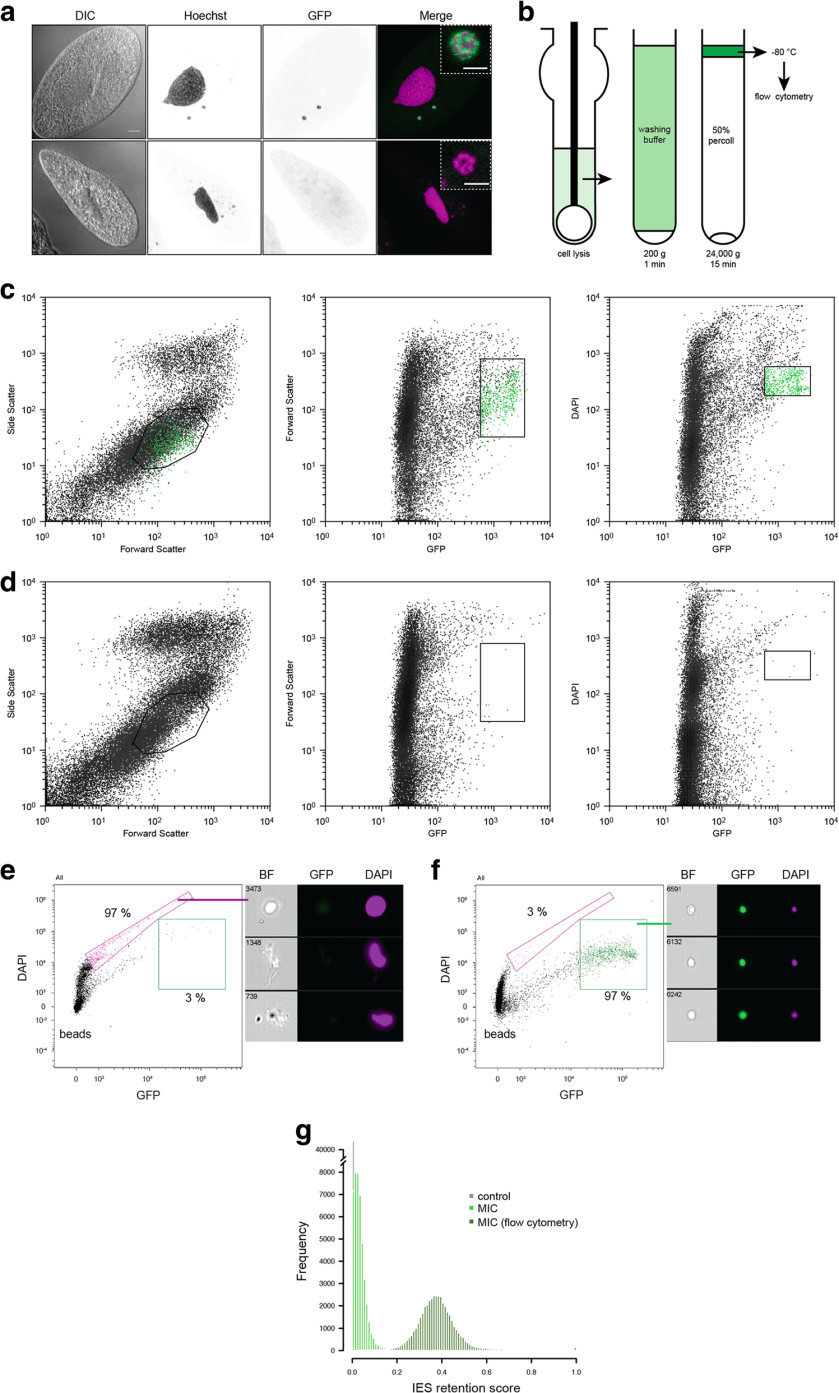
Purification of germline MICs from vegetative *Paramecium tetraurelia* by flow cytometry and validation by flow cell imaging and high throughout DNA sequencing. **a**. In *CENH3a*-*GFP* transgenic *Paramecium* vegetative cells (*upper panels*), but not in control cells (*lower panels*), the MICs are GFP positive. Scale bar is 10 microns. Higher magnification: Scale bar is 3 microns. **b**. Fractionation scheme used to isolate the MIC-enriched fraction. **c** - **d**. Multi-gating strategy used for sorting the MICs. Sorting is based on size, granularity and DAPI staining and GFP signals in **c**) CENH3a-GFP transgenic cells and **d**) control cells. An empiric iterative procedure coupled with flow imaging allowed discrimination between MICs and DAPI containing contaminants, identification of the population of interest, and optimization of the sorting strategy. **e** - **f**. The Amnis ImageStream^X^imaging flow cytometer is used for quality control: sample before (**e**) and after sorting (**f**). **g.** Validation of the sorting strategy by high-throughput DNA sequencing. Histograms of IES retention scores are shown for control (no RNAi), MIC (no sorting) and MIC after sorting (flow cytometry)

We used a similar strategy to sort the small germline MICs from vegetative cells (Fig. 3 and Additional file 1: Figure S1). The available MIC isolation method, that relies on Percoll density gradient centrifugation [7], does not provide a MIC fraction sufficiently pure for exclusive MIC genome sequencing, owing to contamination from i) the MAC DNA (800n vs 2n in MIC), and ii) bacteria, on which *Paramecium* cells feed. MIC isolation has been achieved in other ciliates [12–14] but the same methods were not successful in *Paramecium*. We hypothesized that the contamination issues can be solved by the use of a specific fluorophore that is unambiguously and exclusively associated with the MICs. We previously generated transgenic *Paramecium* cells that constitutively express a MIC-localized version of the Green Fluorescent Protein (GFP) fused to centromeric histone H3 (CenH3a) [15]. Transgenic *CENH3a*-*GFP* cells have green fluorescent MICs, but neither the MAC nor the bacteria are GFP positive (Fig. 3a). We used the same fractionation scheme as the one previously published, with some improvements, to enrich for MICs [7] (Fig. 3b), and submitted the sample to flow cytometry. MICs were sorted based on the SSC, FSC, DAPI (DNA staining) and GFP signals (Fig. 3c-d). The procedure was optimized by flow cell imaging to define the population of interest and refine the sorting parameters (Additional file 1: Figure S1). We obtained 528,000 MICs from 3 million cells.

As previously, purity before and after sorting was measured by flow cell imaging. The MICs represented only 3% of the total sample before sorting and 97% after sorting (Fig. 3e-f). Thus, the sorting procedure is indispensable for effective MIC purification. We performed high throughput Illumina sequencing of the DNA extracted from sorted MICs (528,000 sorted MICs; 60 ng) and from the MIC-enriched sample before sorting. As expected, the bacterial DNA contamination greatly diminished after sorting (8.2% of known contaminants before and 0.2% of known contaminants after sorting) (Additional file 2: Table S1). We identified 44,851 IESs in the sorted sample, but only 5,192 IESs in the unsorted nuclear fraction. Calculation of mean IES retention scores indicated that enrichment in MIC-limited sequences increased from 0.04 in MIC DNA to 0.38 in sorted MIC DNA (Fig. 3g). The fact that 97% MIC purity only led to approximately 40% MIC DNA is explained by the much higher DNA content of the 3% MAC-derived contaminating fraction. We conclude that flow cytometry sorting is necessary to directly sequence all IESs in unperturbed cells. The fact that 97% (*n* = 43,741) of the IESs identified in the sorted MIC DNA correspond to the same IESs identified in the sorted PGM DNA confirms that the genome-wide set of IESs in PGM DNA reflects the complete set of MIC IESs (Additional file 1: Figure S2). These data demonstrate that the Pgm domesticated transposase is required for the excision of all IESs.

### A first glimpse of the germline genome reveals new transposable elements

The sequence complexity of the MIC assembly is presented in Table 1. Coverage by MAC reads was used to define MAC-destined as opposed to MIC-limited compartments. The 98 Mb assembly consists of 74 Mb (~75%) of MAC-destined sequences and 24 Mb (~25%) of MIC-limited sequences, consistent with the size of the MAC reference genome assembly (72 Mb, [6]). It is important to realize that the MIC assembly we have obtained is highly fragmented (N50 = 37 kb; half of the assembly is in contigs smaller than 37 kb). The most fragmented part of the assembly is the MIC-limited compartment (N50 = 13 kb; half of the MIC-limited sequence is in contigs smaller than 13 kb). With such an assembly, it is possible to annotate germline-limited elements such as IESs and transposable elements (TEs), but not to analyze long-range features such as chromosome structure. For that, additional information, e.g. from mate-pair libraries or third generation long read sequencing, is necessary to handle repeats and build scaffolds.

**Table 1:** Characterization of MIC contigs

	MIC assembly	MAC-destined	MIC-limited
Complexity	98 489 268 bp	74 212 942 bp (75.4%)	24 276 326 bp (24.6%)
N50	37.2 Kb	46.9 Kb	12.7 Kb
GC content	27.40%	27.97%	25.66%
IES	3 517 996 bp	147 387 bp (4.2%)	3 370 609 bp (95.8%)
Transposable Elements	2 973 685 bp	237 838 bp (8%)	2 735 847 bp (92%)
Tandem Repeats	1 393 130 bp	485 112 bp (34.8%)	908 018 bp (65.2%)

The MIC assembly consists of all of the contigs assembled using Velvet as launched by ParTIES [11] (Additional file 2: Table S2). The MIC-limited and the MAC-destined parts of the assembly are defined as a function of MAC read depth, using the 3 MAC datasets described in (Additional file 2: Table S1). Any nucleotide with a MAC read depth <20× is considered MIC-limited, else the nucleotide is MAC-destined. N50 means that half an assembly is contained in contigs larger than the N50 value. The MIC-limited part of the assembly is thus much more fragmented than the MAC-destined part. The number of nucleotides covered by Internal Eliminated Sequences (IES), Transposable Elements (TE) and Tandem Repeats (TR) are given. MIC-limited sequences contain almost all IESs and TEs, 95.8% and 92% respectively. The majority (65%) of TR are found in the MIC-limited sequences, however 35%, reflecting WD40, TPR and other repeats, are found in the MAC-destined compartment.

The MIC contigs were used to identify TEs, starting from three previously identified *Paramecium* DNA transposons [3, 16] and a partial reverse transcriptase (RT) consensus (see Methods). tblastn searches using the DDE transposases or RT as queries identified a number of distinct elements, and potentially functional consensus sequences were reconstructed in most cases from the alignment of 10–20 copies (full range 4–48). The majority of TEs (*n* = 38) are Class I non-LTR retrotransposons, while 13 belong to the IS630-Tc1-mariner (ITm) super-family of Class II DNA transposons. The remaining consensus sequences are putative non-autonomous Class I SINE or solo-ORF1 elements. Characterization of the elements is provided (Additional file 3: Table S3 and Table S4). This analysis significantly augments knowledge of TE in the *Paramecium* germline and presents the first *Paramecium* Class I elements.

**Fig. 4:**
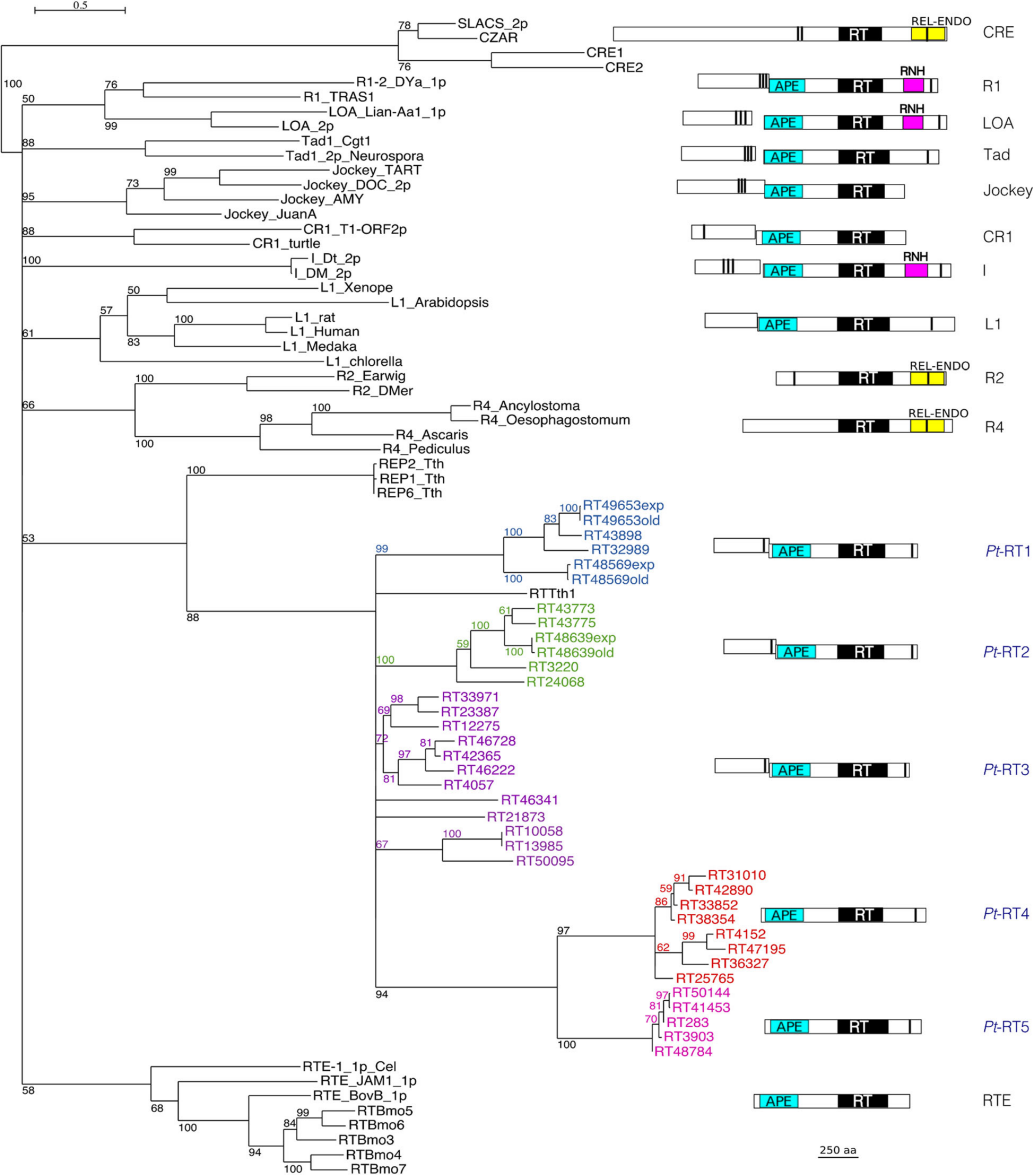
Phylogeny of non-LTR elements based on their RT domains. The phylogeny is based on the alignment shown in (Additional file 1: Figure S3) of the ~ 250 aa catalytic RT domains of the elements listed in (Additional file 2: Table S5). The phylogeny is a 50% maximum likelihood tree, rooted with the CRE clade. The numbers at nodes represent the percentage of bootstrap values for 100 replicates. Clade names are prefixed to the element names for the 11 major non-LTR clades. The ciliate non-LTR form a new clade. The names of the elements for the 5 *Paramecium* groups are colored: *blue*, Group 1; *green*, Group 2; *magenta*, Group 3; *red*, Group 4; *pink*, Group 5. The amino acid divergence scale is indicated. Schematic diagrams of ORF structure of representative *Paramecium* elements from each group and representatives of the 11 major clades identified in [18] are shown next to the phylogeny. The representatives are the same as in [18]; however for Tad, Tad1 from *N. crassa* is shown; for R1, TRAS1 from *B. mori* is shown; and for I, the element from *D. melanogaster* is shown. The domains are RT, reverse transcriptase; APE, apurinic/apyrimidinic endonuclease; REL-ENDO, restriction enzyme-like endonuclease; RNH, RNase H domain. Vertical bars represent zinc-finger domains. The two ORFs are shown as offset whether or not they are in the same frame. For Group 1 and Group 2, there is a +1 frameshift. For Group 3, the two ORFs are in the same frame

The non-LTR retrotransposons all have an ORF2 that contains both apurinic/apyrimidinic endonuclease (APE) and reverse transcriptase (RT) domains, like most known groups of non-LTR retrotransposons [17, 18]. They fall in 5 groups, the first 3 of which also contain an upstream ORF1 (Fig. 4). A phylogeny was built using an alignment of the *Paramecium* RT domains with those of elements belonging to 11 previously characterized major clades [18] (Fig. 4, Additional file 1: Figure S3, Additional file 2: Table S5). The *Paramecium* retrotransposons, along with elements from the ciliate *Tetrahymena thermophila* [19], emerge as a distinct new clade in the tree, with good branch support. The consensus sequences of the first 3 groups, which contain an ORF1, suggest that ORF2 translation depends on +1 ribosomal frameshifting or translation re-initiation (Groups 1 and 2), or on translational read-through of the ORF1 stop codon (Group 3). Like other non-LTR retrotransposons [20, 21], these elements contain short stretches of variable tri-, tetra-, or penta-nucleotide repeats at their 3′ ends (Additional file 3: Table S3). Seven elements (solo ORF1s A-G) appear to contain only an ORF1, ending with a zinc finger similar to that found at the C-terminal end of ORF1 in Groups 1–3, and are likely mobilized in *trans* by proteins encoded by other elements; a (TAAA) n repeat was found at the end of the element in 3 cases.

**Fig. 5:**
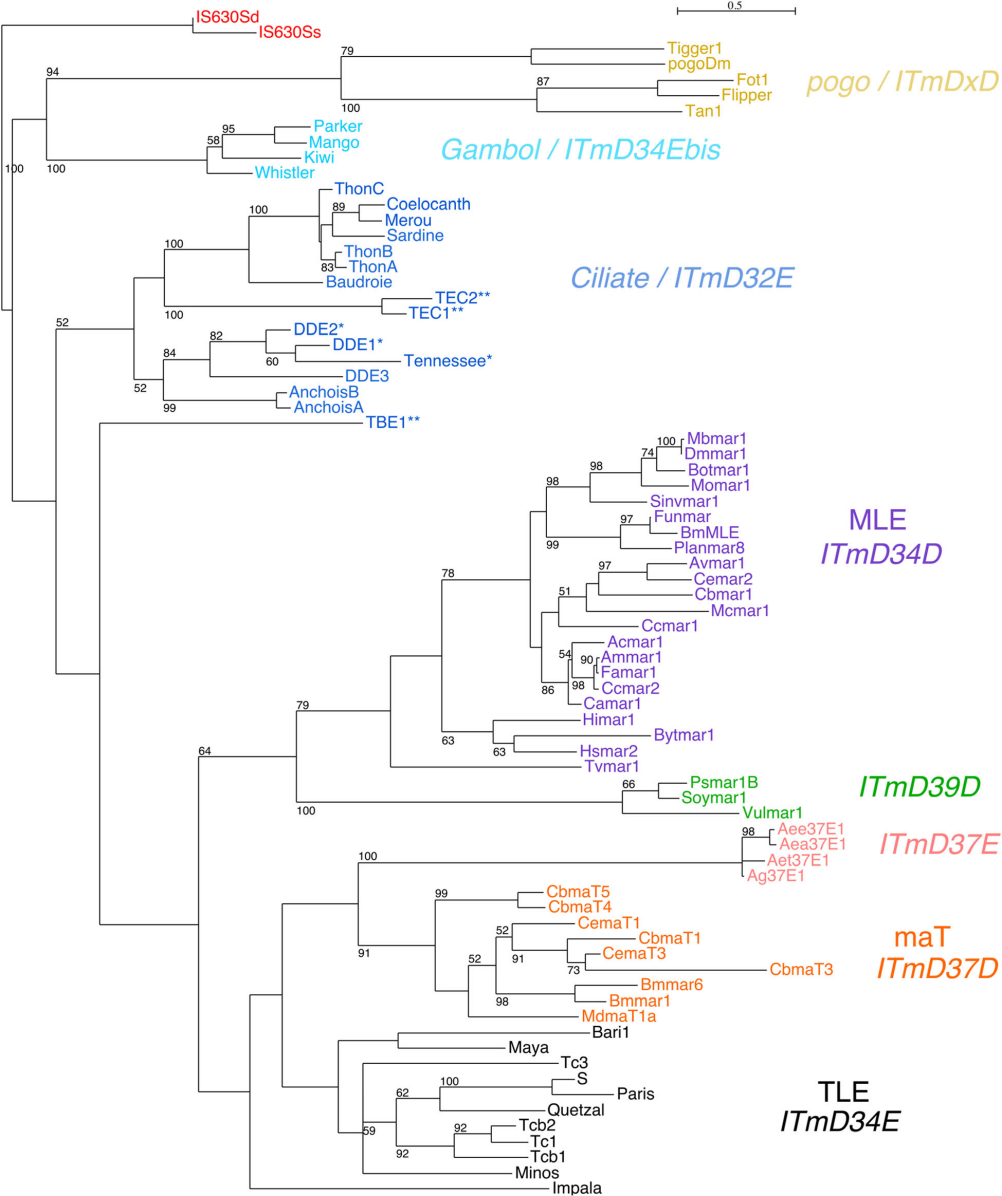
Phylogeny of ITm elements based on their DDE domains. The phylogeny is based on the alignment shown in (Additional file 1: Figure S5) of the ~ 150 aa catalytic DDE domains of the elements listed in (Additional file 2: Table S6). The phylogeny is a maximum likelihood tree, rooted with bacterial IS630 elements. The numbers at some nodes represent the percentage of bootstrap values for 100 replicates if 50% or greater. Clade names are in bold to the right of the tree. As in [22, 26], the names include the distance between the last two catalytic residues. Most of the elements in the ciliate clade are D32E, however those with one star are D33E and those with two stars are D34E. The amino acid divergence scale is indicated

The 13 DNA transposons, all of the ITm superfamily [22], are unusual in that they contain multiple ORFs (Additional file 3: Table S4). In addition to the DDE ORF common to all ITm elements, an ORF2 of unknown function is found in all *Paramecium* transposons and shares detectable sequence similarity among all of them (Additional file 1: Figure S4). The largest *Paramecium* transposons contain 4 ORFs, ORF4 being a tyrosine recombinase, a property shared with TEC and TBE transposons from distantly related ciliates [23–25]. As seen in the Maximum Likelihood tree built using many ITm DDE domains [22, 26] (Fig. 5, Additional file 1: Figure S5, Additional file 2: Table S6), the composite *Paramecium* elements with a tyrosine recombinase group together, along with TEC1 and TEC2. A distance of 32 aa between the second and third residues of the DDE catalytic triad, characteristic of the 7 tyrosine-recombinase containing *Paramecium* ITm and 3 of the 6 simpler elements, is among the shortest ever reported for ITm.

RepeatMasker was used to identify copies of the TEs in the MIC contigs. Tandem Repeat Finder was used to identify putative satellite sequences (see Methods). As shown in Table 1, 96% of the short unique copy IESs and 92% of the TE copies are in the MIC-limited compartment. However, about one third of tandem repeats were found in the MAC-destined compartment and include WD40, TPR and surface antigen repeats.

### MIC and PGM DNA are not equivalent

To compare the sorted MIC DNA with the unrearranged DNA from *PGM*-silenced cells, used until now to represent germline DNA, we calculated the depth of coverage of the MIC assembly by the sorted MIC DNA and the sorted PGM DNA sequencing datasets. The calculation was performed for 90,017 non-overlapping 1-kb windows.

**Fig. 6:**
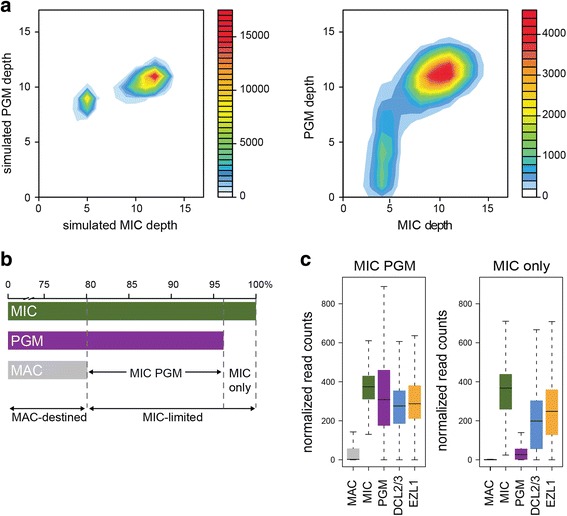
Coverage of the MIC assembly by different sequencing samples. **a**. Global comparison of the sequences in simulated (*left*) and real (*right*) PGM and MIC sequencing samples. Depth is calculated by mapping reads to the MIC assembly, and counting the reads in 1-kb non-overlapping windows. The graph shows the density of windows as a heat map color, for each combination of MIC and PGM normalized depth values. **b**. Representation of the genomic compartments identified by analysis of differential read coverage of the MIC assembly (cf. Methods, DESeq2 analysis and Table 2). The horizontal bars show the percentage of the MIC assembly covered by each sequencing sample, defining three genomic sub-compartments. “MAC-destined” is the genomic compartment covered by MIC, MAC and PGM reads, i.e. windows with no differential coverage according to the DESeq2 analysis; “MIC PGM” is the compartment covered by MIC and PGM reads; “MIC-only” is the compartment covered only by MIC reads. **c**. Barplots of the normalized DESeq2 read counts, across all windows and all samples (Additional file 2: Table S1) for the “MIC PGM” compartment (*left*) and the “MIC-only” compartment (*right*)

We visualized the comparison between the two datasets by creating dot plots of the depth for each window, and representing the density of the dots using heat map colors. To help interpret the comparison, we simulated PGM and MIC datasets, using enrichments in MIC-limited sequences of 80 and 40% respectively (see Methods). As shown in Fig. 6a left plot, the simulated data present two clouds of points. The larger cloud, with the higher depth of coverage in both samples, corresponds to windows present in both the MIC and the MAC DNA. The smaller cloud, with lower depth of coverage in both samples, represents sequence windows present only in MIC DNA. The real data deviates from this unbiased profile (Fig. 6a, right). The larger clouds representing windows present in both MIC and MAC DNA are comparable (Additional file 1: Figure S6). Surprisingly, the smaller cloud is now vertically elongated, indicating that genome coverage in the PGM DNA is variable and mostly less covered than expected (depth between 0 and 7). Both PGM samples behave in the same way. The same windows are found to be under-represented in both PGM and unsorted PGM samples (Additional file 1: Figure S6).

To refine this observation and determine which sequences are missing from the PGM DNA, we used the uniquely-mapped read counts in the 1-kb non-overlapping windows to identify differentially covered windows, in the same way as RNA-Seq counts for genes are used to identify differentially expressed genes (see Methods). The statistical software package we used takes into account the small number of independent samples (2 or 3 biological replicates for most samples, Additional file 2: Table S1).

We looked for windows less covered by MAC or PGM reads with respect to MIC reads (Additional file 1: Figure S7). This allowed us to define three genomic compartments (Fig. 6b, Table 2): 80% of the MIC genome non-overlapping windows are not differentially covered and represent the part of the germline genome that is collinear with MAC chromosomes (“MAC-destined”). The remaining 20% of the windows was significantly less covered by MAC than by MIC reads, corresponding to the germline-limited part of the genome (“MIC-limited”). As anticipated by the previous analysis of read depth, ~ 3% of the windows not covered by MAC reads are not well-covered by the PGM reads. We thus subdivided the MIC-limited compartment into “MIC PGM” and “MIC-only” sub-compartments (Fig. 6b). Figure 6c shows barplots of the normalized read counts of the windows for each of the samples, for the “MIC PGM” and “MIC-only” sub-compartments. As expected, the two sub-compartments are not covered by MAC reads and are well-covered by MIC reads. Interestingly, the “MIC-only” sub-compartment, which is poorly covered by PGM reads, is well-covered by DCL2/3 and EZL1 reads (Additional file 1: Figure S6). These two factors are required for developmental DNA elimination and act respectively in small RNA and histone post-translational modification pathways upstream of the introduction of DNA double-strand breaks by the Pgm endonuclease [27].

**Table 2:** Characterization of different sub-compartments of the MIC assembly

	MAC-destined	MIC PGM	MIC only
Complexity	76 130 194 bp	15 983 936 bp	2 905 995 bp
Genome proportion	80.12%	16.82%	3.06%
Longest contiguous region	286 000 bp	58 000 bp	79 000 bp
GC content	27.58%	26.29%	27.47%
IES	97.70%	2.26%	0.04%
Low complexity	5.40%	5.50%	5.43%
Tandem repeats	0.83%	1.39%	5.95%
TE	1.33% (0.97 Mb)	21.47% (3.43 Mb)	7.35% (0.23 Mb)
not TE	98.67% (75.16 Mb)	78.53% (12.56 Mb)	92.65% (2.68 Mb)
*TIR*	0.45% (0.33 Mb)	4.96% (0.79 Mb)	0.53% (0.02 Mb)
*LINE*	0.84% (0.62 Mb)	15.98% (2.56 Mb)	6.57% (0.2 Mb)
*SINE*	0.04% (0.03 Mb)	0.53% (0.08 Mb)	0.25% (0.01 Mb)

Columns from left to right: “MAC-destined” is the genomic compartment covered by MIC, MAC and PGM reads (i.e. windows with no differential coverage according to the DESeq2 analysis, see Methods); “MIC PGM” is the sub-compartment covered by MIC and PGM reads; “MIC only” is the sub-compartment covered only by MIC reads. These compartments are represented schematically in Fig. 6b. The IES reference set was mapped to the MIC assembly and then the IESs were assigned to a window. The total complexity of tandem repeats (micro- and mini-satellite) was calculated using Tandem Repeats Finder. Low complexity sequences identified by Repeat Masker include stretches of poly-purine or poly-pyrimidine and regions of high AT (>87%) or high GC (>89%) content. Repeat Masker was also used to find TE copies, using the TE consensus library reported in this study (See Methods, Additional file 4: Text S1 and Text S2. The difference between the “MIC PGM” and the “MIC only” sub-compartments was judged highly significant for Tandem repeats and for TE (*p*-value: 9.88e-324 and 9.45e-105, respectively). The MIC only sub-compartment, representing germline-limited sequences not present in either of the PGM samples, is thus enriched in satellites and depleted in TEs.

Different sequence characteristics were calculated for the three genomic sub-compartments (Table 2). GC content and low complexity content did not vary across sub-compartments. Approximately 99% of the IES reference set could be mapped to the MIC assembly. Since 90% of IESs are shorter than 100 bp (median IES size 51 nt) it is not surprising that nearly all IES-containing 1-kb windows are covered by MAC reads and are thus found in the “MAC-destined” compartment.

The TE consensus library was used to find TE copies in the 3 genomic sub-compartments (Table 2). The important difference between the 16 Mb “MIC PGM” and the 3 Mb “MIC-only” sub-compartments is that the latter is significantly depleted in TE copies and enriched in tandem repeats i.e. micro- and mini-satellite (Table 2).

We can suggest two possible, non-exclusive explanations for why 3 Mb of sequence complexity present in the MIC DNA are absent from the PGM DNA: i) the Pgm domesticated transposase is not needed for the elimination of some MIC-limited sequences or ii) *PGM* RNAi is released at the end of development and this release is sufficient for elimination of some MIC-limited sequences. Consistent with the latter explanation, depletion of other factors involved in programmed DNA elimination, and whose function is likely upstream of Pgm, did not lead to underrepresentation of MIC-limited sequences (Fig. 6c and Additional file 1: Figure S6). Whatever the reason of the under-representation of MIC-limited sequences upon *PGM* RNAi, it indicates that PGM DNA, used up until now as a proxy for MIC DNA, does not provide a faithful representation of the MIC genome.

## Conclusions

We report the development of an efficient flow cytometry-based method to sort nuclei in *P. tetraurelia*. This method represents a major breakthrough over previously published methods [3, 7], in that it provides (i) improved reliability; (ii) high purity; and (iii) quality control evaluated by flow cell imaging and high throughput sequencing. Our work also provides a clear demonstration that flow cell imaging is a powerful means to detect the population of interest and help refine sorting parameters.

We expect that cytometry-based purification of subpopulations of macronuclei during development may allow kinetic studies of the DNA elimination and endoreplication processes. We have shown that our procedure allows high throughput Illumina sequencing of the *P. tetraurelia* germline genome, paving the way for sequencing the germline genome of other *P. aurelia* sibling species for future comparative genomic studies.

So far, only a few studies have made use of flow cytometry to sort nuclei [28–40], mostly in plants and neurons. Our work highlights the unique potential of flow cytometry to analyze and sort heterogeneous populations of nuclei. It demonstrates that flow cytometry and sorting provide a powerful way to purify minority subpopulations of nuclei, provided that specific nuclear characteristics or a specific fluorophore can be unambiguously and exclusively associated with the subpopulation of interest.

The contigs assembled from the sorted MIC DNA have allowed discovery of 61 germline TEs. The majority are Class I non-LTR retro-transposons (LINE elements), never before characterized in *Paramecium*. This library of manually curated TE consensus sequences constitutes a precious resource for future automated approaches to TE identification and classification in the germline genomes of *Paramecium* species, especially given the relatively large phylogenetic distances to related elements from other taxa.

## Methods

### Cells and cultivation

All experiments were carried out with the entirely homozygous strain 51 of *Paramecium tetraurelia*. Cells were grown in a wheat grass powder (WGP, Pines International, USA) infusion medium bacterized the day before use with *Klebsiella pneumoniae*, unless otherwise stated, and supplemented with 0.8 mg/L of β-sitosterol (Merck). Cultivation and autogamy were carried out at 27 °C.

### Developing MAC purification

We used the feeding method described in [41] to silence the *PGM* gene. *Escherichia coli* HT115 [42] harboring plasmid L4440 [43], with the 567-bp HindIII-NcoI fragment of the *PGM* gene inserted between two convergent T7 promoters [9], was induced for the production of PGM dsRNA in WGP1X medium containing 100 μg/mL ampicillin by overnight growth at 37 °C with shaking. The next day, the culture was diluted into the same medium to OD600 = 0.04. IPTG (Euromedex) was added at a final concentration of 0.4 mM to induce dsRNA synthesis. After 4 h of induction at 37 °C with shaking, the medium was cooled down to 27 °C, and supplemented with 0.8 mg/L of β-sitosterol just before use.

*P. tetraurelia* cells were first grown in standard *K. pneumoniae* medium for 20–30 vegetative fissions then washed twice in silencing medium. Cells were allowed to grow for 8 to 10 vegetative fissions in a final volume of 3 L of silencing medium (freshly induced medium was added the second day) then starved to trigger autogamy. Progression of autogamy was monitored by Hoechst staining (Sigma). At day 4 of starvation, 30 autogamous cells were picked and transferred individually to 200 μL of *K. pneumoniae* medium to monitor the viability of sexual progeny and evaluate the efficiency of *PGM* silencing. As expected, *PGM* RNAi led to high rates of lethality in the sexual progeny.

At day 4 of starvation, cells were 100% autogamous with about 90% of cells displaying two large developing MACs. Purification of developing new MACs was performed using the protocol described in [3] with minor modifications. Cultures were filtered on 8 layers of sterile gauze. Cells were centrifuged at 600 g for 1 min in an oil-testing centrifuge (Sigma 6–16, rotor 13116) then washed in 100 mL of Tris–HCl 10 mM pH 7.4 and centrifuged again to obtain a compact pellet (~1 mL). After centrifugation, the cell pellet was resuspended in 2 volumes of lysis buffer (~2 mL) (0.25 M sucrose; 10 mM MgCl2; 10 mM Tris pH 6.8; 0.2% NP40) and kept on ice for 5 min. All steps were performed at 4 °C. Cells were then lysed with a Dounce homogenizer until approximately 90% of the cells were broken as observed under a microscope (×20). Developing MACs were collected by centrifugation at 1,000 g for 1 min. The pellet that contained the developing MACs was washed twice with 9 volumes (~9 mL) of washing buffer. The pellet was then resuspended in 2 mL sucrose solution (2.1 M sucrose; 10 mM MgCl2; 10 mM Tris pH 7.4) and loaded on top of a 3 mL sucrose solution layer in an Ultra-clear centrifuge tube (Beckman Coulter 344059). After gentle addition of washing buffer to fill the tubes, the samples were centrifuged at 210,000 g for 1 h, in a SW41ti swinging rotor (Optima L-80 XP ultracentrifuge, Beckman Coulter). After centrifugation, the sucrose solution was carefully removed. The pellet was gently rinsed with washing buffer, before resuspension into ~ 3 mL of washing buffer containing glycerol (13% final concentration). The samples were aliquoted and frozen at −80 °C.

### Micronuclei purification

Transgenic *Paramecium* cells expressing a micronuclear (MIC)-localized version of the Green Fluorescent Protein (GFP) were obtained by microinjection of the vegetative macronucleus with the CenH3a-GFP plasmid, described in [15], in which the centromeric histone variant (CenH3a) gene fused to GFP is expressed under the control of the constitutive promoter of the elongation factor Tu. In the transformed clones, GFP was exclusively found in the MICs. Transformed clones were selected for their GFP signal/noise ratio. Transgene quantification indicated a copy number close to the endogenous CenH3a gene level (transgene/endogenous gene ~ 0.6 to 1). Viability of the sexual progeny after autogamy of the transformed clones was systematically monitored to make sure that the presence of the transgene did not impair the functionality of the MICs.

Transformed and non-injected cells were grown in standard *K. pneumoniae* medium in a final volume of 3 L at a cell density of 1,000 to 1,500 cells/mL. The vegetative state of the cells was assessed by nuclear staining with a 33:1 (vol/vol) mix of carmine red (0.5% in 45% acetic acid) and fast green (1% in ethanol). Detection of GFP signal in the MICs was monitored in the transformed cells. Cultures were filtered on 8 layers of sterile gauze. Cells were centrifuged at 600 g for 1 min in an oil-testing centrifuge (Sigma 6–16, rotor 13116) then washed in 100 mL of Tris–HCl 10 mM pH 7.4 and centrifuged again to obtain a compact pellet.

We used the same fractionation scheme as the one previously published to enrich in MICs [7] with some improvements. After centrifugation, the cell pellet was resuspended in 2 volumes of lysis buffer (0.25 M sucrose; 10 mM MgCl2; 10 mM Tris pH 6.8; 0.2% NP40) and kept on ice for 5 min. All steps were performed at 4 °C. Cells were then lysed with a Dounce homogenizer until approximately 90% of the cells were broken as observed under a microscope (×20). Three volumes of washing buffer (0.25 M sucrose; 10 mM Tris pH 7.4; 5 mM MgCl2; 15 mM NaCl; 60 mM KCl; 0.5 mM EGTA) were added. The sample was dispatched into 2 mL Eppendorf tubes and mixed by inversion 5 times then centrifuged at 200 g for 1 min. The supernatant that contained most MICs was recovered and presence of the MICs was verified under a microscope. The supernatant was then transferred into Ultra-clear centrifuge tubes (Beckman Coulter 344059, 2 mL per tube), and 10 mL of 50% Percoll solution (50% Percoll pH 7.5; 0.25 M Sucrose; 10 mM MgCl2) were added drop by drop with gentle agitation. The supernatant and the Percoll solution were gently mixed by pipetting and centrifuged at 24,000 g for 15 min in a SW41Ti swinging rotor (Optima L-80 XP ultracentrifuge, Beckman Coulter). During centrifugation, the Percoll gradient is formed and MICs accumulated at the top of the gradient and MACs at the bottom. After centrifugation, MICs were carefully recovered in a white-to-brown powderous band with a 200 μL Pipetman into a 1.5 mL Eppendorf tube. The MIC-enriched sample was gently mixed then diluted 1/1/1 with washing buffer and glycerol 40% (13% glycerol final concentration). Usually several hundred MICs per microliter could be counted under a microscope. The samples were aliquoted and frozen at − 80 °C for further flow cytometry analysis and sorting.

### Flow cytometry

Samples of MICs and developing MACs were thawed on ice, diluted 1/5 to 1/10 in washing buffer and stained with DAPI (3 μM final, Invitrogen #D3571). All steps were performed at 4 °C. The samples were filtered (30 μm Sysmex filters, 04-004-2326) and sorted on an Influx 500 cell sorter (BD Biosciences) with a 488 nm laser for scatter measurements (Forward Scatter, or FCS, and Side Scatter, or SSC) and GFP excitation, and a 355 nm laser for DAPI excitation. GFP and DAPI staining signals were collected using a 528–38 nm band pass filter and a 460–50 nm band pass filter, respectively. Phosphate Buffered Saline (Isoflow TM Sheath Fluid, Beckman Coulter) was used as sheath and run at a constant pressure of 15 PSI. Frequency of drop formation was 27 kHz. The instrument used a 100 μm nozzle. For the MIC samples, a threshold on the GFP signal was optimized to increase collecting speed (2500 events per second). For developing MACs, an important threshold on FCS was optimized to not consider the crystals present in the sample and increase collecting speed. *Paramecium* cells contain crystals composed of guanine, xanthine and hypoxanthine [44], which are pelleted together with developing MACs during the purification procedure and can represent an important part of the elements detected by the instrument. Since they do not contain DNA, hiding crystals allowed a faster collecting speed without increasing DNA contamination. Sorting rates typically ranged from 10,000 to 100,000 MICs per hour depending on the preparation. Data were collected using Spigot software. Micronuclei were sorted based on their SSC, FSC, GFP and DAPI signals. Events in GFP and DAPI gates were backgated onto FSC vs SSC to optimize the sorting. Developing MACs were sorted based on their SSC, FSC, DAPI, and time-of-flight (pulse width) signals. Events with high DPAI signal were backgated onto FSC vs SSC to optimize the sorting. Nuclei were recovered in washing buffer into a 1.5 mL Eppendorf tube.

### Flow cell imaging

Purity of the sorted samples was evaluated by flow cell imaging. Samples before and after sorting were imaged on a 2 camera, 12 channel ImageStream^X^ (Amnis/MerckMillipore) imaging flow cytometer with a 60× magnification, using 405, 488, and 785 nm lasers, at respectively 125, 100, and 0.05 mW. Phosphate Buffered Saline (137 mM NaCl; 2.7 mM KCl; 6.7 mM Na2HPO4; 1.5 mM KH2PO4) was used as sheath. Acquisitions were performed using Inspire software. Brightfield was collected in channel 1 and 9, SSC in channel 6 (745–800 nm bandwidth), GFP in channel 2 (480–560 nm bandwidth), and DAPI in channel 7 (430–505 nm bandwidth). At least 5,000 elements were analyzed for each sample (before and after sorting) in order to detect enough MICs, given the rarity of MICs in the sample (~0.2–3% of all events detected by the Influx cell sorter before sorting). Cell classifiers were set for channel 1 area lower limit of 10 to allow the instrument to focus despite low concentration of the sample after sorting. Beads were excluded from the analysis based on their low DAPI and GFP fluorescence signals. Analysis was performed using the IDEAS software.

### Genomic DNA extraction and sequencing

After sorting, MICs and developing MACs were treated with 3 volumes of proteinase K solution (0.5 M EDTA pH 9; 1% N-lauroylsarcosine; 1% SDS; 1 mg/mL proteinase K) at 55 °C overnight. Genomic DNA was extracted with the addition of one volume of Tris–HCl-phenol pH 8 with gentle agitation at room temperature for 1 h (no vortex). After centrifugation at 300 g for 15 min, the aqueous phase was recovered, dialyzed twice against TE (10 mM Tris–HCl; 1 mM EDTA, pH 8) 25% ethanol for 2 h, against TE overnight, then against Tris 1 mM pH 8 for 2 h. DNA was concentrated with a Concentrator plus (Eppendorf) down to 50 to 100 μL. DNA concentration was quantified using QuBit High sensibility kit (Invitrogen) and stored at 4 °C. DNA was then sequenced by a paired-end strategy using Illumina Hi-Seq next-generation sequencer (Additional file 2: Table S1). DNA-seq datasets have been deposited at the NCBI short read archive (SRA) (Accession numbers: SAMN05323659; SAMN05323660; SAMN05323661).

### Transposable element annotation

Putative LINE elements were discovered as follows. Reverse transcriptase coding domains were identified from a small cluster of homologous sequences retained in the MAC genome, after building a consensus from their alignment. These partial peptide sequences were then used to search the MIC contigs (tblastn using default parameters, with no low complexity filter). The matches were culled and used to extend the consensus protein sequences. Then blastn searches (default parameters, no low complexity filter) were used against the MIC contigs to find more copies. The procedure was used recursively to extend and find as many copies as possible. Copies were aligned with MUSCLE [45] and adjusted manually, with a requirement of potentially functional ORF1 and ORF2 sequences. Finally, the best adjusted consensus sequences were used to search for other related elements by a tblastn search for long, poorly scoring matches which might be recent copies of a different element. In this way, 5 distinct groups of LINE elements were found. A similar procedure was used to annotate Class II DNA transposons, starting from published sequences for the *P. primaurelia* Tennessee element ORFs [16] and the *P. tetraurelia* Sardine and Anchois element ORFs [3]. Finally, some sequences inserted in other elements were found to be present in multiple copies in the MIC assembly but yielded consensus sequences with no protein-coding potential; these sequences were annotated as putative SINE elements. Fasta files with the nucleotide and putative peptide sequences are provided (Additional file 4: Text S1-S2), (Additional file 3: Tables S3-S4).

### Phylogenetic tree reconstruction

Non-LTR Class I retrotransposon ORF2 (pol) protein sequences representative of different clades [18] and IS630-Tc1-mariner (ITm) superfamily transposase protein sequences [22, 26] were recovered from GenBank or RepBase (Additional file 2: Tables S5-S6). Corresponding *Paramecium* consensus sequences were added to each set of proteins. The proteins were aligned using MSAProbs [46]. The alignments were trimmed manually to correspond to the RT and DDE catalytic domains, respectively (Additional file 1: Figures S3 and S5) and used for phylogenetic tree reconstruction by Maximum Likelihood [47, 48], with PhyML version 3.1 (PhyML -d aa -m LG -v 0.0 -c 4 -a E -f M --no_memory_check -i < phylip_alignment_file > −b 100). The non-LTR retrotransposon RT tree was collapsed if branch support (determined using 100 bootstrap replicates) was less than 50%, using TreeGraph2 [49]. Seaview [50] was used for preliminary tree-building, to convert alignment formats and to visualize, re-root, swap branches and prepare figures of the trees.

### Bioinformatic analyses

#### IES retention

IES retention scores were calculated with ParTIES v1.0 [11] (MIRET module, −max_mismatch 1 –score –method Boundaries) using the *P. tetraurelia* IES reference set [3] and two reference genome assemblies available from ParameciumDB (http://paramecium.cgm.cnrs-gif.fr/download/fasta/assemblies/): ptetraurelia_mac_51.fa and ptetraurelia_mac_51_with_ies.fa. The score for each IES corresponds to the mean of the two boundary scores.

#### Assembly of MIC reads

The MIC flow cytometry sequencing reads (acc. no. SAMN05323660; Additional file 2: Table S1) were assembled into contigs using ParTIES v1.0 [11] (default parameters except for the Assembly module, −k 51). ParTIES filters out reads that contain a MAC IES junction using the MAC reference genome prior to a Velvet (version 1.2.10) [51] assembly. Assembly statistics for the resulting MIC contigs are given in (Additional file 2: Table S2).

#### Analysis of depth

The MIC contigs (Additional file 2: Table S2) were used as reference genome for this analysis. The contigs were divided into 1-kb non-overlapping windows. For each sequencing sample, the mean depth for each window was calculated with Samtools [52] depth (v0.1.18 –q 30 –Q 30) on Bowtie 2 [53] (v2.2.3 –local –× 800) mappings. The mean depth was normalized according to the number of nucleotides sequenced in the sample, after excluding reads which match known contaminants (mitochondrial DNA, rDNA, bacterial genomes).

#### Sequencing simulation

We simulated sequencing data using ART version 2.3.7 [54] (−−noALN --len 100 --seqSys HS10 --qShift 90 --qShift2 90 --mflen 300 --sdev 100). We specifed coverage using the fcov parameter, to obtain final coverage of 100×. Thus, to obtain a dataset with 40% enrichment in MIC sequences, we simulated 40× coverage on the MIC assembly and 60× coverage on the MAC assembly and pooled the simulated reads. The analysis of depth was applied to the simulated read datasets.

#### Differential coverage analysis

DESeq2 software [55] was designed for differential analysis of NGS count data, and is typically used for gene expression studies i.e. to compare RNA-Seq read counts for genes across experimental conditions. We used DESeq2 (v. 1.14.0) to compare DNA-Seq read counts for non-overlapping MIC windows (1 kb windows and >400 bp windows at contig ends) across samples. For each sample (Additional file 2: Table S1), we provide to DESeq2 the number of uniquely mapping reads in each window. We considered windows with a fold-change >2 between MIC and other samples and an adj.*p*-value < 0.05 to be differentially covered. Barplots (Fig. 6c) used the normalized counts determined for each sample by DESeq2.

#### Sequence properties

For selected windows (see text and Table 2), tandem repeats (micro- and mini-satellite) were identified using Tandem Repeats Finder [56] (version 4.07b, TRF parameters: 2 7 7 80 10 50 500) and the corresponding complexity determined using the R Bioconductor package “GenomicRanges_1.26.1” [57]. RepeatMasker [58] (version 3.3.0) was used to identify low complexity sequences (RepeatMasker -noint –no_is –s) and transposable elements (TE; RepeatMasker -nolow –no_is –s –lib < TE consensus library>). The TE consensus library is that reported in this study (Additional file 3: Tables S3-S4). We performed exact binomial tests using the R package binom_1.1–1 [59].

## Additional files

Additional file 1: This PDF contains the following supplementary figures: **Figures S1- S7.** Legends for these figures appear at the beginning of Additional file 1 (PDF 12230 kb)

Additional file 2: This word file contains the following supplementary tables: **Tables S1, S2, S5, S6.** (DOCX 110 kb)

Additional file 3: This excel file contains the following supplementary tables: **Tables S3-S4.** (XLSX 45 kb)

Additional file 4: This text file contains the following supplementary text: Text S1-S2. Text S1 is a fasta file of TE consensus nucleotide sequences. Text S2 is a fasta file of putative TE protein sequences. (TXT 280 kb)

## Abbreviations

APE: Apurinic/apyrimidinic endonuclease
FSC: Forward-scattered light
GFP: Green fluorescent protein
IES: Internal Eliminated Sequence
ITm: IS630-Tc1-mariner
MAC: Macronucleus
MIC: Micronucleus
RT: Reverse transcriptase
SSC: Side-scattered light
TE: Transposable element
