# Supplementary material for: Flow cytometry sorting of nuclei enables the first global characterization of Paramecium germline DNA and transposable elements
Source: BMC Genomics. 2017 Apr 26;18:327. doi: 10.1186/s12864-017-3713-7 (PMC5405496; doi:10.1186/s12864-017-3713-7)
Supplement: Supplementary file 1 — This PDF contains the following supplementary figures: Figures S1- S7. Legends for these figures appear at the beginning of Additional file 1 (PDF 12230 kb) [file 12864_2017_3713_MOESM1_ESM.pdf]

Figure Legends

Figure S1: Overview of the approach.
Paramecium cells, which contain two types of nuclei of different size, chromatin composition and ploidy, were lysed and used to isolate specific subpopulations of nuclei (in red: MICs, in blue: MAC) by flow cytometry. Flow cell imaging was used to detect the population of interest, help refine sorting parameters and assess quality control. DNA extracted from isolated nuclei was used for high throughput sequencing, providing further validation of the method.

Figure S2: Venn Diagram of IESs found in MIC and PGM reads.
The Venn diagram show that ~ 97% of IESs identified using sorted MIC reads, published PGM reads [1] and sorted PGM reads are common to the three datasets.

Figure S3: **Alignment of RT domains used for phylogeny.**
The RT domains defined here correspond to NCBI's RT_nLTR_like conserved domain (cd01650) and are slightly shorter than those used in [2]. Numbers in red above the alignment indicate the positions of conserved motifs 1-7, as defined in Figures 1 and 2 of [2].

Figure S4. Alignment of ORF2 domains of *Paramecium* ITm elements.
Amino acids containing the ORF2 intron are shown in red.

Figure S5. Alignment of DDE domains used for phylogeny.
The catalytic domains of ITm transposases were here defined to include the DDE or DDD triad. Red asterisks above the alignment indicate the positions of the 3 acidic residues.

Figure S6: Coverage depth after RNAi of different genes involved in programmed DNA elimination.
The plots compare depth of coverage in each non-overlapping 1-kb window of the MIC assembly (contigs > 2kb) for pairs of samples and the density of windows with the same values is indicated by heatmap colors. a and b, simulated and real PGM with MIC comparison, reproduced from Figure 6a. c. EZL1 with MIC comparison, d. DCL2/DCL3 with MIC comparison. e. MAC with MIC comparison, showing that the smaller cloud representing the germline-limited windows is centred at 0 in the MAC DNA. Accession numbers for the samples used: see [Additional File 3: Table S1].

Figure S7: DESeq2 differentially covered windows.
The diagram uses rectangles to show the relationships between the windows differentially covered for contrasts between MIC and each of the other samples (see Methods and Table S1). Thus a contrast between MIC and MAC will give “not MAC” i.e. windows significantly less covered by MAC than by MIC reads. The fewer the number of differentially covered windows, the more similar the sample is to MIC. Note that 35 of the windows in the “not PGM” set are MAC windows; these windows are probably false positives (indicating a false positive rate of ~ 1%). The genomic compartment “MAC-destined” corresponds to the green shading, “MIC PGM” corresponds to the gray shading and “MIC-only” corresponds to the purple shading.

REFERENCES

1. Arnaiz O, Mathy N, Baudry C, Malinsky S, Aury J-M, Wilkes CD, et al. The Paramecium germline genome provides a niche for intragenic parasitic DNA: evolutionary dynamics of internal eliminated sequences. PLoS Genet. 2012;8:e1002984.

2. Malik HS, Burke WD, Eickbush TH. The age and evolution of non-LTR retrotransposable elements. Mol. Biol. Evol. 1999;16:793–805.

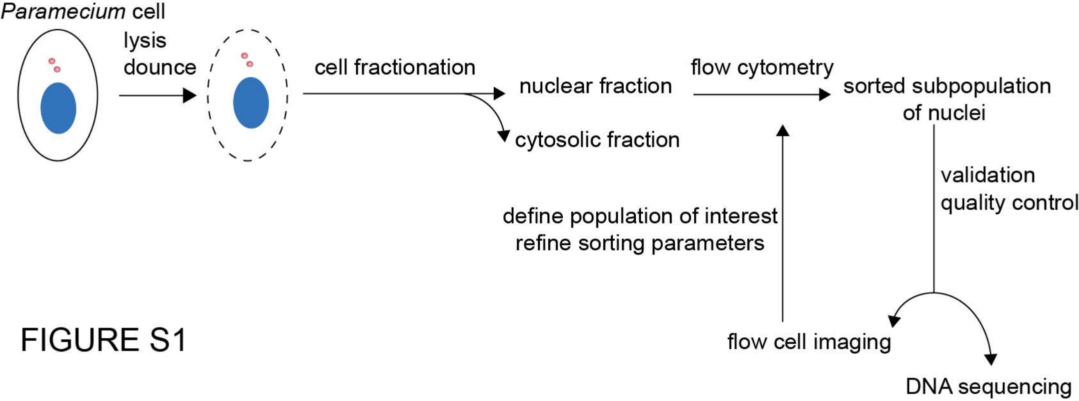

Paramecium cell
lysis
dounce
cell fractionation
nuclear fraction
cytosolic fraction
flow cytometry
sorted subpopulation of nuclei
validation
quality control
define population of interest
refine sorting parameters
flow cell imaging
DNA sequencing

FIGURE S1

FIGURE S2

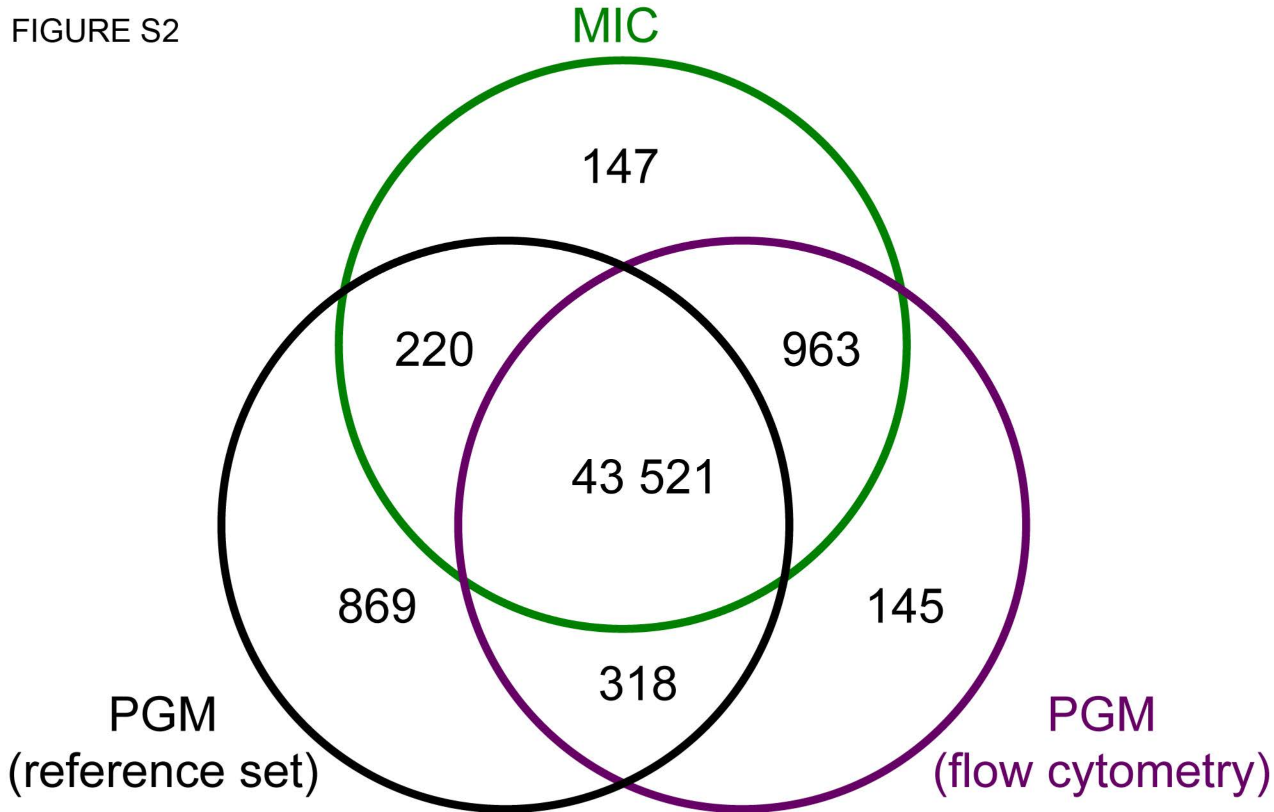
MIC
147
220
963
43 521
869
145
318
PGM
(reference set)
PGM
(flow cytometry)

```
                                  1                        2                               2a
SLACS 2p            1 ATSLTVL----RKP-N------------GKYRPIGAESVWAKLASHIAISRVMKTAE--K----KFSGIQFGVGG-----
CZAR                1 ATNLTVL----RKP-N------------KKFPPIGAECVWAKAISLMAVDAVMPALK--T----CFKNLQYGVGN-----
CRE1                1 SSAVTPI----PKD-E----------AGTKIRPIVPESAWLKLASLVAMAEIPSSFK--E----TFKGWQYGVWG-----
CRE2                1 ACILHPF----RKE-A----------GSAKVRPITPESALMKLAAHIALDSVEKSFR--S----TFKGWQYGVWG-----
R1-2_DYa_1p         1 LQRLVLI----SKG-K---GD---PLTPSAYRPLCMLNTTGKLLEKMIKPRLSAAIERGG----GLSPRQHGFRPGRSTI
R1 TRAS1            1 AATVVIL----RKS-G---KDS--YTEPKSHRPIGLLPVLGKLYEKMLVARLKYHLLP------RMSTRQFGFMPQRSTE
LOA Lian-Aa1 1p     1 DITVKFI----PKG-G---RAS--YEEAKSFRPISLTSFLLKCLERIIDHHIRDVYLANM----PLHVNQHAYQSGKSTV
LOA_2p              1 RARVVFI----PKA-G---KKD--ITDPKSFRPISLTSFLLKTLEKMVDYKIRSTLLKQR----PLHPAQHAYRVGRSTD
Tad1 Cgt1           1 KAEVVMI----QKL-G---KTD--FSKTGSWRPIALLSCLGKGLERLIAKRMAYLTILEG----VASPQQVGALPGRAAV
Tad1 2p Neurosp     1 DAKVVVL----KKP-G---KSAAQQKLAGAWRPISLLSNVGKILEALVAKRLTQAAEEFN----LLPEGQMGNRAGRSTE
Jockey_TART         1 HAQVKMI----LKP-G---KS---ANEPRSYRPISLLSGLSKIFERLLLKRLFKVDLFKK----AIPLHQFGFRKEHGSE
Jockey DOC 2p       1 KSTIVMI----PKP-G---KD---KTQPSSYRPISLLTCLSKLFEKMLLLRISPHLRINN----TLPTHQFGFREKHGTI
Jockey_AMY          1 EADVIGI----HKP-G---KP---KNDPTSYRPISLLMSLGKLYERLLYKRLRDFVSSKG----ILIDEQFGFRTNHSCV
Jockey_JuanA        1 NAKVVPI----LKP-D---KN---PAEASSYRPISLLSSISKLFEKVILNRMMAHINENS----IFANEQFGFRHGHSTT
CR1 T1-ORF2p        1 SCWLFPV----HKK-G---C----RSIVSNYRGITQTCATAKTFELCIFPTILHSCSS------AISPKQHGFMPGRSTS
CR1_turtle          1 KANIVPI----FKK-G---K----KEDPGNYRPVSLTSXPGKIMEQVLKESILRHLEERK----VIRXSQHGFTKGKSCL
I_Dt_2p             1 TSLIIPI----LKP-N---TD---KTKTTSYRPISLNCCIAKKLDKIIAKRLWWLVTHSN----LLSENQFGFKKGKSTS
I DM 2p             1 TSLIIPI----LKP-N---TD---KTKTSSYRPISLNCCIAKILDKIIAKRLWWLVTYNN----LINDKQFGFKKGKSTS
L1_Xenope           1 RAVLSLL----PKK-G----D---LRLIKNWRPVSLLSTDYKIVAKAISLRLKSVLA--E----VIHPDQSYTVPGRTIF
L1_Arabidopsis      1 STILALI----PKK-K----E---AKEMKDYRPISCCNVLYKVISKIIANRLKLVLP--K----FIVGNQSAFVKDRLLI
L1 rat              1 EATITLI----PKP-H---KD---TTKKENFRPISLMNINAKILNKILANRIQEHIK--T----IIHHDQVGFIPGMQGW
L1_Human            1 EASIILI----PKP-G---RD---TTKKENFRPISLMNIDAKILNKILANRIQQHIK--K----LIHHDQVGFIPGMQGW
L1_Medaka           1 EAIISII----PKE-G---KD---RLDCANYRPVSVLNIDYKLFTSIISRRLETILP--M----LMHKDQTGFIKQRQTQ
L1 chlorella        1 QGRITLL----YKGKG---AD---RESLASYRPITLLNTDYKLAARAIASRIGPLLN--Q----VVDATQTGFLPKRWAG
R2_Earwig           1 ESKTTLI----PKK-P----N---STEPGDFRPITVQSVLVRQLNKILAARVAQHIP--------LDERQRGFRPVDGVA
R2_DMer             1 MARTIFI----PKT-V----R---ANRPQDFRPISVPSIVVRQLNAILASRLTAAVS--------WDPRQRGFLPTDGCA
R4 Ancylostoma      1 QGRVVLI----PKE-LKRGQS---TRGPGDYRPIACLNTCYKILTASMSAQVLRTIG--D----RFPQEQIALRKGVWGC
R4_Oesophagosto     1 EGRVVLI----PKE-LKGDQS---IRGPGDFRPIACLNVSYKILTASMSTRILQSVG--D----RFPREQIALRKGVWGC
R4_Ascaris          1 RGRTILI----PKK-G----D---RGDPSNYRPITCLNTCYKVLTSVMNSVILSHLSRGE----ALPMNQRAMRKREWGC
R4 Pediculus        1 TGKTYLL----PKK-S----G---ATEPKDFRPITCLPTMYKIITAIIAEKIYGHLRKNN----IFPPEQYGCRKGSYGC
REP2_Tth            1 ARQIYLL----KS-------E---SRQIINCRPITIQSSAIKILENAMLQHVQKLKD--EGKVKDFHISQCGFQKNRSTI
REP1_Tth            1 ARQIYLL----KS-------E---SRQIINCRPITIQSSAIKILENAMLQHVQKLKD--EGKVKDFHISQCGFQKNRSTI
REP6 Tth            1 ARQIYLL----KS-------E---SRQIINCRPITIQSSAIKILENAMLQHVQKLKD--EGKVKDFHISQCGFQKNRSTI
RT49653exp          1 EFQIIPL----NKC-Y---PQ---IPQVGEFRPITILSPLYKWIELRFEKALRQYCE--R----KIIKQQVGFIRGLGTH
RT49653old          1 EFQIIPL----NKC-Y---PQ---IPQIGEFRPITILSPLYKWIELRFEKALRQYCE--R----KIIKQQVGFIRGLGTH
RT43898             1 EFYIIPL----NKR-Y---PN---IPQVDEFRPITILSPLYKWIELRFERSLRLYCE--R----HIIKQQVGFIKGFGTH
RT32989             1 EFFIIPL----NKN-Y---PN---IPQASEFRPITILSPLYKWLELRFEKSLSRYCE--S----KIIKQQVGFIKGFGTQ
RT48569exp          1 ENRLIPL----NKA-F---PN---IPEKDQFRPIIVQSPIMKWLELRFLKKLQQYCE--K----KIIKEQFGFVQGAGTQ
RT48569old          1 ENRLIPL----NKA-F---PN---IPEKDQFRPIIVQSPIMKWLELRFLKKLQQYCE--K----KIIKEQFGFVQGAGTQ
RTTth1              1 AARLIPL----NKV-F---PN---IPSAGDFRPITVTSPAYKWLENRFSTKIQQYLK--E----RLDRNQTGFIEGCGTG
RT43773             1 WVRLCPL----NKC-H---PE---IPSRTQFRPLVIMSPLLKFLELRFHNQLQDYLK--D----KILKPQTGFISGLGTQ
RT43775             1 WVRLCPL----NKC-H---PE---IPKCDQFRPLVIMSPLLKFLELRFHDKLQDYLK--N----RILKPQTGFISGMGTH
RT48639exp          1 WVRLCPL----NKC-H---PE---IPSKEQFRPLVIMSPLFKFLELRFYQKLQDYLR--Y----KILKPQIGFISGMSTH
RT48639old          1 WVRLCPL----NKC-H---PE---IPSKEQFRPLVIISPLFKFLELRFYQKLQDYLR--Y----KILKPQIGFISGMSTH
RT3220              1 WARLLPL----NKM-H---PN---IPKNDQFRPLVILSPLYKFLELRFHSQLQDYLN--N----DIQKPQTGFINGLGTQ
RT24068             1 WVRLIPL----NKV-H---PK---IPTKEQFRPLVILSPLFKFIELRFYQQLMDYMI--H----KIKKEQTGFIKGYGTQ
RT33971             1 RARLVPL----NKV-F---PD---IPTKTQFRPITILSPLFKLLELRFLPKLQQYLS--T----RLTKGQAGFIPGSSTQ
RT23387             1 RARLVPL----NKV-F---PN---IPNKNQFRPITILSPLFKLLELRFLPKLQYYLS--F----RLSKGQAGFIPNSSTQ
RT12275             1 RARIVPL----NKV-Y---PS---IPNKMQFRPISILSPLFKLLELRFLPKLQTYLT--Q----KLSKEQTGFIPFSSTQ
RT46728             1 QARLVPP----NKA-F---PQ---IPSNSQFKPIKILSSLFKFLELRFLPQLQKYIE--T----RLNNQQTGFVSGSSTQ
RT42365             1 QARLIPL----NKV-F---PQ---IPTNSQFRPITVLSSLFKFLELRFLPKLQEYME--T----RLNKQQTGFVPGSSTQ
RT46222             1 QARIIPL----NKA-F---PQ---IPSSTQFRPISVLSSLFKFLELRFLPQLQNYME--I----KLCNQQTGFVPKSSTQ
RT4057              1 QARLIPL----NKV-Y---PK---LPLANEFRPITILSPLFKLLELRFLPKLQQYML--I----KLNKEQIGFVPDQSTQ
RT46341             1 EARLVPL----NKA-F---PQ---IPTKGQFRPITILSNLYKFMELRFKGKIDDYMQ--K----RMTIKQNGFVKGGGTS
RT21873             1 EARLIPL----NKV-Y---PL---IPKTSQFRPITVFSALYKFIELRFLNKINEFLQ--N----QVSNRQNGFVKGGGTQ
RT10058             1 EARLVPL----NKA-Y---PD---IPSQFQFRPIVVMSPLFKFLELRFATQLQTYNL--N----NLKFYQAGFIQGRGTQ
RT13985             1 EARLVPL----NKA-Y---PD---IPSQFQFRPIVVMSPLFKFLELRFATQLQTYNL--N----NLKFYQAGFIQGRGTQ
RT50095             1 KARIVPL----NKN-Y---PE---IPTKQQFRPITILSPLLKFMELRFMGKLQNYCK--E----NLRFYQAGFIKGRGTQ
RT31010             1 AQRLIPL----NKI-A---PQ---IAGVNDIRPIMVQSHLVKFMEWSFMKAFKQANS--L----RVDPCQIGFTSKLGTQ
RT42890             1 AQRLIPL----NKV-A---PR---IAGVNDVRPIMVQSHLVKFMEWSFMKAFKQANS--L----RVDPCQIGFTSKLGTQ
RT33852             1 AQRLIPL----NKV-A---PK---IAGVDDVRPIMVQSHLVKFMEWSFMKAFKLANS--L----RIEPCQVGFTSKLGTQ
RT38354             1 AQRLIPL----NKI-A---PK---IAGVNDVRPIMVQSHFVKFMEWSFMKAFKLANS--L----RIEPCQVGFTSKLGTQ
RT4152              1 AQRLIPL----NKV-A---PK---IAGVDDVRPIMVQSHLVKYMEWMFMDALKNINS--L----RLDPCQVGFTSKLGTQ
RT47195             1 AQRLIPL----NKV-A---PK---IAGVDDVRPIMVQSHLVKYMEWMFMDALKNINN--L----RLDPCQVGFTSKLGTQ
RT36327             1 AQRLIPL----NKV-A---PK---IASVDDVRPIMVQSHLVKFMEWSYMAAFKNINS--L----RIDPCQVGFTSKLGTQ
RT25765             1 AQRLIPL----NKV-A---PK---IASVDDVRPIMVQSHLVKFMEWSFMKAFKLTNS--L----RMDPCQVGFTSKLGTQ
RT50144             1 SMRLIPL----NKM-H---PM---IPIRGQFRPIMVISHFVKLFEASYVQKFKKICS--S----KINPSQIGFVSYLGCS
RT41453             1 SMRLIPL----NKM-H---PM---IPIRGQFRPIMVISHFVKLFEASYVQKFKTICS--S----KINPSQIGFVSYLGCS
RT283               1 SMRLIPL----NKM-H---PQ---IPIRGQFRPIMVISHFVKLFEASYVQKFKKICT--S----KINPSQIGFVSYLGCS
RT3903              1 SMRLIPL----NKM-H---PK---IPIRGQFRPIMVISHFVKLFEASYVQKFKDICT--S----KINPSQIGFVSYLGCS
RT48784             1 SMRLIPL----NKM-H---PQ---IPIRGQFRPIMVISHFVKLFEASYVQKFKKICT--S----KINPSQIGFVSYLGCQ
RTE-1 1p Cel        1 TSKTTLI----FKK-G----D---RENLENYRPICLLPVLYKVFTKCLLNRMRRSLD--E----AQPVEQAGFRRSFSTI
RTE_JAM1_1p         1 QGVICPI----YKK-G----D---KLECENYRAITILNAAYKVLSQILFRRLSPIAN--E----FVGSYQAGFIDGRSTT
RTE_BovB_1p         1 RSVFIPI----PKK-G----N---AKECSNYRTIALISHASKVMLKILQARLQQYVN--R----ELPDVQAGFRKGRGTR
RTBmo5              1 KSIILPL----HKK-G----S---TRKCDNYRTLALVSHCSKVLLYILNTRLRYFLD--W----QIPQEQAGFVKGKGTR
RTBmo6              1 SSIILPHKKALHKK-G----S---TKDCNNYRTLALISHASKILLHIINSRIRHFLD--W----QIPQEQAGFVKGRGTR
RTBmo3              1 ESLLIPL----HKK-G----S---TRTCSNYRTVALISHASKVMLYVINGRLQSYIQ--W----QIPSEQAGFVKGRGTR
RTBmo4              1 HSIFIPL----HKK-G----S---TKKCNNYRLISLVSHASKVMLHIINTRLQGYLS--R----EIAPEQAGFVKGRGTR
RTBmo7              1 HTVFVPL----HKK-G----S---TKSCSNYRLIALIPHACKILLRIVNERLKSYLS--K----EIAPEQAGFVKGKGTR
```

FIGURE S3 1/5

```
                                                                        3
SLACS 2p              53 -HIEEAI-A-KIRK-DF-------AT-KGSLAMLDGRNAYNAISRRAILEAVYG-DSTW-----SPLWRLVSLLLGTTGE
CZAR                  53 -NIELAI-Q-KIRR-DF-------HL-KGSVAMLDGRNAYNAISRTAILSAVYG-NTAW-----SPLWRVTRLLLGTEGL
CRE1                  55 -DVAKAV-A-KIRR-DS-------EE-HEYLVALDGVNAYNTMSRAHILQAVYA-EQRL-----KPIWGVVKVALGGPGF
CRE2                  55 -DSTEAV-K-RIRE-AYA--E---AS-SDTLVALDATNAYNRMSRRHILEAAYA-QPEL-----RFAFGVVNLSLGAAGE
R1-2_DYa_1p           66 GAIQEVV-S-QALS-SQQ--GNHFSRPVVLLATLDVKNAFNSVQWGNIIDALKRRFQIS-----GYLMRIIQNYLDNR-R
R1 TRAS1              65 DSLYTLM-Q-HVKE-KLK--E---KR-IITIVSLDIEGAFDSAWWPALEVRLAE-EKCP-----EYLRRVISSYLSDR-R
LOA Lian-Aa1 1p       67 TLLHKVV-Y-DIEK-AFA--Q---KQ-SCLGVFLDIEGAFDNVSFDAILEAARN-HGLP-----TMITNWIHQMLKNR-H
LOA_2p                67 TALYQLQ-R-TLSA-AID--Y---KE-VALCAFLDIEGAFDNTSHDAIKDTLSR-RGLD-----PTTSRWILALLRSR-Q
Tad1 Cgt1             67 DLTTCLT-H-EIER-ALD--L---GL-TATLVTMDVKGAFDSVLRNRLIIRLRQ-QGWP-----NSLLRLIFTFASSR-T
Tad1 2p Neurosp       69 FAVRVVT-D-AVHT-AWK--L---GA-VTSLMLLDLKGAFDRVNHRWLLHTLWE-MQLP-----TWIIRWVASFVAGR-R
Jockey_TART           66 QQIARVT-Q-FILE-AFE--R---KE-YCSAVFLDISEAFDRVWHEGLLLKLAK-I-LP-----YNLYIILESYLTNR-T
Jockey DOC 2p         66 EQVNRIT-S-EIRT-AFE--H---RE-YCTAIFLDVAQAFDRVWLDGLLFKIIK-L-LP-----QNTHKLLKSYLYNR-V
Jockey_AMY            66 QQVHRLT-E-HILV-GLNRPK---PL-YTGALFFDVAKAFDKVWHNGLIFKLFN-MGVP-----DSLVLIIRDFLSNR-S
Jockey_JuanA          66 HQLLRVT-N-LTRA-NKS--E---GY-STGLALLDIEKAFDSVWHEGLIVKLKN-FNFP-----TYIVRIIQSYLSNR-T
CR1 T1-ORF2p          63 TNLMSFV-T-NIFR-SFE--A---GT-QLDAIYTDFHAAFDSLPHSLLLAKLSK-LGFG-----DGIISWLSSYLSNR-S
CR1_turtle            65 TNLIAFY-E-EVTG-SVD--V---GK-AVDVIYLDFSKAFDTVSHSILISKLKK-YGLD-----EWTIKWIENWLXHR-A
I_Dt_2p               66 DCLLYVD-Y-LITK-SK--------M-HTSLVTLDFSRAFDRVGVHSIIHQLQE-WKTG-----PKIINYIQNFMINR-K
I DM 2p               66 DCLLYVD-Y-LITK-SK--------M-HTSLVTLDFSRAFDRVGVHSIIQQLQE-WKTG-----PKIIKYIKNFMSNR-K
L1_Xenope             63 DNVFLIR-D-LLHF-ARR--T---GL-SLAFLSLDQEKAFDRVDHQYLIGTLQA-YSFG-----PQFVGYLKTMYASA-E
L1_Arabidopsis        63 ENVLLAT-E-IVKD-YHK-DS---VS-SRCALKIDISKAFDSVQWKFLINVLEA-MNFP-----PEFTHWITLCITTA-S
L1 rat                64 FNIRKTI-N-VIHY-INK-LK---EQ-NHMIISLDAEKAFDKIQHPFMIKVLER-IGIQ-----GPYLNIVKAIYSKP-V
L1_Human              64 FNIRKSI-N-VIQH-INR-AK---DK-NHMIISIDAEKAFDKIQQPFMLKTLNK-LGID-----GTYFKIIRAIYDKP-T
L1_Medaka             64 DSIRKVL-N-IIHQ-VVQ--Q---KQ-ETLVISLDAEKAFDSVRWTFLYKVLGK-FGFC-----KSIIETISGLYNKP-T
L1 chlorella          65 DNVLAHL-E-EISY-LEA--T---HQ-PGVQVFLDFEKAFDRLDRAWIERCMAA-VGFG-----PGVQRWVHILHSGT-T
R2_Earwig             61 HNIFELD-M-ILRC-HRS--E---FR-DLRLASLDIAKAFDSITHNTIEDTMEV-RGFP-----KPMINYIMACYRRS-K
R2_DMer               61 DNATIVD-L-VLRD-HHK--R---YA-SCYIATLDVSKAFDSVAHDAVFNTVTA-YGAP-----KSFVDYVRRWYSGG-G
R4 Ancylostoma        67 THAQILD-QTVVKD-ALR--H---KK-ELHMLWVDLTKAFDSLSHGAIRWTIKQ-WGVP-----SDLRRLLSTIMSMQ-S
R4_Oesophagosto       67 THAQILD-QTVIRD-AMR--H---KK-ELHMLWVDLTKAFDSLSHGSIRWAVKQ-WGVP-----SDVRRMLTVLMSLQ-S
R4_Ascaris            65 THAMVLDRAMVMDA-MAQ--K---KH-SLSVAWLDYRKAYDSVSHEYIRWAINS-VNIP-----RSVQLTLKRLMSDW-E
R4 Pediculus          65 KEVLLIN-KLIMAS-AKQ--K---RK-NLSMAWIDYQKAFDSVPHEWIIEALKI-YKVD-----PNITAFCEKSMKNW-C
REP2_Tth              65 INICRTI-A-LIQS-TVA--K---KE-NRVAIFVDVKSAFDSVNHEQLFQALRN-QGFD-----DIFIKSVAFLYQH---
REP1_Tth              65 INICRTI-A-LIQS-TVA--K---KE-NRVAIFVDVKSAFDSVNHEQLFQALRN-QGFD-----DIFIKSVAFLYQH---
REP6 Tth              65 INICRTI-A-LIQS-TVA--K---KE-NRVAIFVDVKSAFDSVNHEQLFQALRN-QGFD-----DIFIKSVAFLYQH---
RT49653exp            64 VQILKLI-Q-DIQK-F-K--A---AN-NRLVLFIDFKSAYNTANLDTLFQYMQE-KQILE----QKELQFLRALISNQ-Y
RT49653old            64 VQILKLI-Q-DIQK-F-K--A---AN-NRLVLFIDFKSAYNTANLDTLFQYMQE-KQILE----QKELQFLRALISNQ-Y
RT43898               64 VQILKLI-Q-DIQK-F-K--A---RD-KRLILFIDFKSAYNTANLELLFQYMDK-KQILQ----EAELKFLKALISNQ-Y
RT32989               64 FQILKLI-K-DISR-L-R--A---YD-NKLVIFIDFKSAYNTANLDLLYRYMQE-KTILA----INELNFLKALISNQ-Y
RT48569exp            64 PQILKLI-T-NMQQ-F-K--K---KD-RRVVIFIDFRSAYNCAQIDDLFNFLKE-MAVLD----EDELNFLQSLTSWQ-Y
RT48569old            64 PQILKLI-T-NMQQ-F-K--K---KD-NRVVIFIDFRSAYNCAQIGELFNFLKE-MAVLD----EDELNFLQSLMSWQ-Y
RTTth1                64 LNIFMLI-N-KIQQ-M-R--K---KD-AVGALFIDFKSAYNTICRDRLFKILQD-KDILN----LEESQFLRYLQNNL-F
RT43773               64 LNIFSIL-S-WIKQ-T-T--I---SN-NQCLLFIDFTSAYNTIDRHRLYGILKE-QQVLT----DLEVDYLAAIHSRI-I
RT43775               64 VNIFLLL-D-WVRQ-T-E--K---RN-NQCLIFIDFTSAYNTIHRQRLYEILKE-RRTLS----EVEVDYLEAIHSRI-L
RT48639exp            64 VNIFLMM-N-WIKQ-M-D--N---KN-RYCLIYIDFTSAYNTIDRQRLYGILKQ-RKILD----DQEVDYLEAIHSRI-V
RT48639old            64 VNIFLMM-N-WIKQ-M-D--N---KN-RYCLIYIDFTSAYNTIDRQRLYGILKQ-RKILD----DQEVDYLEAIHSRI-V
RT3220                64 LNIFMIL-N-WIKN-I-K--G---KD-NRILIFIDFSSAYNTICRSKLYGILKK-QNILK----QNEIDFLKALHSRM-L
RT24068               64 TNLLLIK-D-IQKN-Y-T--K---KD-QKVIIFVDFSSAYNTIDRYRLYQLLSM-NNILP----EEEIRFLQAIHSRI-L
RT33971               64 INIIRLL-N-TLTG-Y-K--K---QD-KMDCIFIDFSNAFNSINRQRLFNILSS-KSILD----GIEVKFLNHLYTNL-H
RT23387               64 INITRLL-N-ILVN-L-K--K---QD-KMNCVFIDFSNAFNSINRHKLFSILKS-KAILN----KIEIEFLSNLYTNL-H
RT12275               64 INITNLL-N-RING-F-T--K---RD-KMNCIFIDFSNAFNSVDRGKLFQILRQ-KQILP----QQEIHFLQNLYQNV-Y
RT46728               64 INITNIL-N-LINS-F-K--K---QE-KMNCIFIDFSNAFSCVNRSLLFQILSK-KKILN----NQDIDFLHNLYSNL-F
RT42365               64 INITNIL-N-LINS-F-K--K---QD-KMNCIFIDFSNAFNCVNRSLLYHILKK-KQILS----TQDIDFLRNLYSNL-F
RT46222               64 IHITNIL-N-LINS-F-R--K---AD-RMNCIFIDFSNAFNSVNRTLLFQILID-KQILF----LQDILFLKNLYTNV-Y
RT4057                64 IHITNLL-N-LINS-Y-Q--R---RD-KMNCIFIDFSNAFNSINRPKLFQILKK-KIL-D----TEEISYLYNLYTNL-H
RT46341               64 INLFNIF-Q-QIQK-Y-K--K---QD-KVSLLFIDFSSAYNTIRREKLYQILRN-KNIME----EKEIQFLEAMHSQL-H
RT21873               64 LNITQII-K-LMNQ-Y-N--K---DD-NMDFIFIDYSNAYNTVDRNKLYDIMKK-KQVLN----DLEISFIQALQDQV-Y
RT10058               64 INIASII-H-KINN-T-T--K---EQ-KLDAVFIDFSNAYNTIHRKKLYEILKV-RQILQ----DDEIDFLKALHSNL-Y
RT13985               64 INIASII-H-KINN-T-T--K---EQ-KLDAVFIDFSNAYNTIHRKKLYEILKV-RQILQ----DDEIDFLKALHSNL-Y
RT50095               64 VNISQLI-V-NIKE-K-S--R---EL-KWNAIFIDYSNAYNTIIREKLYQILKQ-RQVLD----EAEIEFIRGIQSQI-Y
RT31010               64 VHISQLV-D-RMK----K--K---GS-GKSFVFFDLSDAYNTIHREAAHHLIKI-KKIID----EDRLNFLFNVYDQL-Y
RT42890               64 VHISQLL-S-RMK----N--K---ES-GKSFIFFDLSDAYNTIHREAAHHLIKT-KKIID----EDRLNFLFNVYDSL-Y
RT33852               64 VHIGQIL-S-KMK----M--K---GT-GKNFIFFDLSDAYNTIHREAAYALIKS-KNIID----EDRLNFLFNVYDSL-Y
RT38354               64 VHIGQIL-S-KMK----S--K---GS-GKNFVFFDLSDAYNTIHREAAYEMIRS-KNIID----EDRLNFLFNVYDSL-Y
RT4152                64 VHISKII-S-RMK----T--K---GE-GKSFVFFDLSDAYNTINREALYGMIQM-RRLID----EKRLNFLFNSYDSL-Y
RT47195               64 VHICKII-S-RMK----T--K---GE-GKSFVFFDLSDAYNTINREALYGMIAL-RRLID----EKRLDFLFNCYDSL-Y
RT36327               64 VHINKVI-S-KMK----T--K---GE-GKCFMFFDLSDAYNTINREAVYTMIKE-DNLID----EDKLDFLFNAYDQL-Y
RT25765               64 VHIGQVI-S-RMK----Q--K---GE-GKNFIFFDLSDAYNTIHREALYEMIKQ-KQVID----EDKLNFLFNVYDSL-Y
RT50144               64 QSIDQII-S-ELKDKTKG--L---NK-GRVSVFMDLSNAYNMVRRQDIFKQLRE-NKQYSMMFNQNEIYALQWMYEQL-Y
RT41453               64 QSIDQII-S-ELKDKTKG--L---NK-GRVSVFMDLSNAYNMVRRQDIFKQLRE-NKQYSMMFNQNEIYALQWMYEQL-Y
RT283                 64 QSIDQII-S-ELKNKTKD--L---NK-GRVSVFMDLSNAYNMVRRQDIFKQLRE-NKQYSSMFNENEIYALQWMYEQL-Y
RT3903                64 QSIDQII-S-ELKTKTKD--L---NR-GRVSVFMDLSNAYNMVKRQDIFKYLRE-NKQYASLFNENEIYALQWMYEQL-Y
RT48784               64 QSIDQII-S-ELKTKTKD--L---NK-GRVSIFMDLSNAYNMVKRQDIFKCLRE-NQQYASLFNENEIYALQWIYEQL-Y
RTE-1 1p Cel          63 DHIHSLQ-R-LLEV-GRE--Y---QI-PLTLVFIDFKKAFDSVEHQAIWKSLDE-QGAD-----GAYIDLLKECYKNC-T
RTE_JAM1_1p           63 DQIFSVR-Q-ILQK-CRE--Y---QV-PTHHLFIDFKAAYDSIDRIELWKIMDE-NSFP-----GKLTRLIRATMDGV-Q
RTE_BovB_1p           63 DQIANIR-W-IMEK-ARE--F---QK-NIYFCFIDYAKAFDCVDHNKLWKILKE-MGIP-----DHLTCLLRNLYAGQ-E
RTBmo5                63 EQILNIR-Q-LIER-SYE--F---GT-PMIMCFIDYSKAFDCVKWDHLWKILAE-LGVP-----QHLVLLLHSLYINN-Q
RTBmo6                67 EQILNIR-Q-LIER-CHE--F---DT-PIILCFVDYSKAFDCVGWNCLWRVLQE-LGVP-----MHLKAFLQSLYYGS-Q
RTBmo3                63 EQIVNVR-Q-IIEK-SRE--F---NM-PILLCFIDYTKAFDCVRWDCLWRILRE-MGVP-----QHLVSLIASLYRDG-V
RTBmo4                63 EQLLVMR-Q-IVEK-ARE--F---NI-SLYVCFVDFRKAFDTVKWWKLWLVLTE-MGVP-----QHLVHTIRRLYEDG-T
RTBmo7                63 EQILTVR-Q-IIEK-SRE--F---NK-PTYLCFVDFSKAFDSVKWPKLWETVLA-MGTP-----KHLVHLLRRLYEEG-T
```

FIGURE S3 2/5

```
                                                       4
SLACS 2p          115 VGFYE----N-G-KLCHTWESTRGVRQGMVLGPLLFSIGTLATLRR-----LQ-Q----------T--------------
CZAR              115 VGFYE----K-G-QLVHSWKSTRGVRQGMVLGPVLFSIGTIATLRQ-----LE-S----------S--------------
CRE1              117 LGVYR----D-G-CLKGNLWSTKGIRQGMVLGPLLYATGMAAAIGP-----VR-Q----------R--------------
CRE2              119 LALYE----N-G-AKIHALKSTEGVRQGMVLSPLLFANAMSGIIRP-----LM-E----------M--------------
R1-2_DYa_1p       135 LLYQT------I-AGEKCIDITSGAAQGSILGPELWNISYDEIFHL-----E--M-------------------------
R1 TRAS1          129 VSVRY----A-G-AEY-ERATSKGCVQGSIGGPILWNLLLDPLIHQ-----LQ-A-------------------------
LOA Lian-Aa1 1p   131 LFSTL----R-Q-AAI-RKLSVCGCPQGGVLSPLLWNLVADTLLRQ-----LN-N-------------------------
LOA_2p            131 VTASV----H-D-STV-TVLTTKGCPQGGVLSPLLWSLLVDELLNR-----LT-N-------------------------
Tad1 Cgt1         131 ARVRL----E-D-TTTEDFPLSCGLPQGSPLSPILFLLYIADILAD-----N----------------------------
Tad1 2p Neurosp   133 GSLFF----D-D-ETSRPYAITAGVPQGSPLSPILFILFITPLYRK-----LA-T-------------------------
Jockey_TART       129 FEVKD----QAG-ETSRTGQIGAGVPQGSNLGPLLYSIFSSDMPLPYIYRPS--P-------------------------
Jockey DOC 2p     129 FAIRC----D-T-STSRDCAIEAGVPQGSVLGPILYTLYTADFPID----------------------------------
Jockey_AMY        132 FRYRV----E-G-TRSSPRPLTAGVPQGSVLSPLLFSLFVNDIPRS-----P----------------------------
Jockey_JuanA      130 LQVNY----Q-N-SRSERLPVRAGVPQGSILGPILYNIFTSDLPEL-----P----------------------------
CR1 T1-ORF2p      127 CRVKT----G-S-YLSEEFFCTSGVPQGCVLSPLLFSLFINDVCNV-----LP---------------------------
CR1_turtle        129 QRVVI----N-G-SMSSWQPVTSGVPQGSVLGPVLFNIFINDLEDG-----VD---------------------------
I_Dt_2p           127 IIVRV----G-P-HTSSPLPLSNGIPLGSPISVILFLIAFNKLSNI-----IS-L-------------------------
I DM 2p           127 ITVRV----G-P-HTSSPLPLFNGIPQGSPISVILFLIAFNKLSNI-----IS-L-------------------------
L1_Xenope         127 CLVKI----N-W-SLTAPLAFGRGVRQGCPLSGQLYSLAIEPFLCL-----LR-K----------RL----T--------
L1_Arabidopsis    128 FSVQV----N-G-ELAGVFSSARELRQGCSLSPYLFVISMDVLSKM-----LD-K----------AV----G--------
L1 rat            129 ANIKL----N-G-EKLEAIPLKSGTRQGCPLSPYLFNIVLEVLARP-----IR-K----------QK----E----IK--
L1_Human          129 ANIIL----N-G-QKLEAFPLKTGTRQGCPLSPLLFNIVLEVLARA-----IR-Q----------EK----E----IK--
L1_Medaka         128 ARIKI----N-G-DLTETITLERGTRQGCNMSALLFALYIEPLGQW-----IR-Q----------RA----D----IK--
L1 chlorella      129 SRVAF----N-G-WHTDAFPVAAGVFQGSPLSPLLFVLARAPMAAH-----AR-M----------LA----G-QLAFQ--
R2_Earwig         125 TRFTF----N-G-WISDTVKPTCGVKQGDPLSPILFNLVMDRMIRK-----LP-K----------E-----------V--
R2_DMer           125 TYFNG----G-D-WRSEEFVPARGVKQGDPLSPVLFNLIIDRLLRS-----LP-K----------D-----------I--
R4 Ancylostoma    132 VRYYGFKNGK-V-VTSSRLQIRNGLMQGDTLSPLLFCLAIAPVSHW-----IR-S----------HI----R----PY--
R4_Oesophagosto   132 VRYYGWVNGR-V-RRSSRLQIRNGLMQGDTLSPLLFCLAIAPTSHW-----IR-Q----------NV----K----PY--
R4_Ascaris        131 TRFESTQCRP-K-LRSDKMKVLNGIFQGDSLSPTLFVLCIAPISYA-----LN-K----------GV----GQCQSSS--
R4 Pediculus      130 TQLEV----Q-K-YSSRKIFIKRGIFQGDSLSPLLFCMSLIPLSRQ-----LN-I----------KD--------QGY--
REP2_Tth          127 -------------CRINGYQIGRGVIQGGKLSPILFNYLYEEVRVK-----IL-E----------IW----------K--
REP1_Tth          127 -------------CRINGYQIGRGVIQGGKLSPILFNYLYEEVRVK-----IL-A----------IW----------K--
REP6 Tth          127 -------------CRINGYQIGRGVIQGGKLSPILFNYLYEEVRVK-----IL-E----------IW----------K--
RT49653exp        128 ---IQ--------YEGVKYSFKNGVPQGSILSPYLFDIYTDLFISK-----AN-E----------FN-------------
RT49653old        128 ---IQ--------YEGVKYSFKNGVPQGSILSPYLFDIYTDLFISK-----AN-E----------FN-------------
RT43898           128 ---IV--------HEGVKYSFKNGIPQGSILSPFLFDIYTDLFISK-----AN-E----------IK-------------
RT32989           128 ---YD--------NNGVKYSFRNGVPQGSILSPYLFDIYTDLFMAE-----TK-K----------IN-------------
RT48569exp        128 ---IK--------SKNQKIAFLNGVPQGSVLSPFLFDIYSHILIKK-----ME-Q----------IY-------------
RT48569old        128 ---IK--------SKNQKIAFLNGVPQGSVLSPFLFDIYSHILIKK-----ME-K----------IY-------------
RTTth1            128 ---YQ--------TKSQKVYLRDGVIQGSSLSPGLFNIYMEDVMKT-----LI-S----------QV-------------
RT43773           128 ---YV----D-AYNINNKIQFKNGVPQGSPISPALFNIYLDEFIKE-----LL-K----------AS-------------
RT43775           128 ---YV----D-AYNTENKVYFKDGVPQGSPISPALFNIYLDEFLRE-----LL-Q----------AS-------------
RT48639exp        128 ---YV----D-AYNFKNIIYFQNGVPQGSPISPALFNIYLDEFLKE-----LQ------------IT-------------
RT48639old        128 ---YV----D-AYNFKNIIYFQNGVPQGSPISPALFNIYLDEFLKE-----LL-Q----------IT-------------
RT3220            128 ---FI----N-PYNISEKFYYSKGVPQGSPISPALFNIYLDEYLKD-----LF-N----------LI-------------
RT24068           128 ---YI----H-PHNMQEKFHYKNGVPQGSPISPLLFNVYMDNFLKK-----LS-E----------KL-------------
RT33971           128 ---YI----C-P-QMNTTFYFQNGVPQGSPLSPALFNIYLEDFLAS-----LK-Q----------NC-------------
RT23387           128 ---YI----C-P-QTNTRYYFGNGVPQGSPLSPGLFNIYLEDFLNT-----LK-Q----------NC-------------
RT12275           128 ---YI----C-P-LTNKKYHFANGVPQGSPLSPALFDIYLENFLIE-----LK-K----------AS-------------
RT46728           128 ---YK----D-P-VSNQSYYFDYGVPQGSPLSPALFNIYLEDILDK-----VS-Q----------VS-------------
RT42365           128 ---YK----D-P-VSNQAYYFDYGVPQGSPLSPALFNIYLEDLLDR-----VS-Q----------VS-------------
RT46222           128 ---YD----D-P-ATNQAYYFDNGVPQGSPLSPALFNIYLEDLLDK-----VL-N----------ES-------------
RT4057            127 ---YK----N-P-ISQELYYFHYGVPQGSPLSPSLFNIYLEELLIT-----IK-Q----------QC-------------
RT46341           128 ---YK----E-P-ESNNMYYFKDGVPQGSSLSPGLFNIYLEAFLEQ-----IG-L----------KL-------------
RT21873           128 ---YE----D-P-INKKKISYLNGLHQGSILSPGLFNIYLNEVINQ-----LK-E----------TF-------------
RT10058           128 ---FK----D-PLYKKEKVWLENGVMQGSPISPLLFNIYLDEFLRD-----FE-N----------EY-------------
RT13985           128 ---FK----D-PLYKKEKVWLENGVMQGSPISPLLFNIYLDEFLRD-----FE-N----------EY-------------
RT50095           128 ---FE----D-PINKERKVCFKNGLVQGSPLSPMLFNIYLDELLSE-----VQ-T----------ET-------------
RT31010           126 ---IY----D-P-ILKKKVRFKNGVQQGSALSPFIFNIFFENVIAR-----TK-E----------KI----N----ERIG
RT42890           126 ---IY----D-P-ILKKKVRFKNGVQQGSALSPFIFNIFFENVILM-----MK-D----------KI----I----ERIG
RT33852           126 ---IY----D-P-ILKKKVRFKNGVQQGSALSPFIFNIFFENVILM-----MK-Q----------QI----D----ERIG
RT38354           126 ---IY----D-P-ILKKKVRFKNGVQQGSALSPFIFNIFFENVILM-----MK-Q----------QI----E----ERIN
RT4152            126 ---IY----D-P-IEKKKIRFKNGVQQGSALSPFLFNIFFEEVIQS-----YK-Q----------QI----K----NTIE
RT47195           126 ---VY----D-P-VEKKKIRFKNGVQQGSALSPFLFNIFFEEVIKH-----YK-Q----------QI----R----ETIE
RT36327           126 ---IY----D-P-ITKKKVRFKNGVQQGSALSPFLFNIFFEKVLER-----VK-L----------LA----Q----EQAK
RT25765           126 ---IY----D-P-ILQKKVRFKNGVQQGSSLSPFLFNIFFENVIKS-----TK-L----------KI----N----QYVE
RT50144           134 ---IY----D-P-IAKEKIYFQNGVHQGSLLAPFLFDIYFNQLIDE-----LE-R----------RIRIKLI----CNKI
RT41453           134 ---IY----D-P-IAKEKVYFQNGVHQGSLLAPFLFDIYFNQLIDE-----LE-R----------RIRIKLI----CNKI
RT283             134 ---IY----D-P-IVKEKVYFKNGVHQGSLLAPFLFDIYFNQLIDE-----LE-R----------RIRIKLI----CNKI
RT3903            134 ---VY----D-P-INKENVYFKNGVHQGSLLAPFLFDIYFNQLIDE-----LE-R----------RIRIKLI----CNKI
RT48784           134 ---IY----D-P-INKENVYFKNGVHQGSLLAPFLFDIYFNQLIEE-----LE-R----------RIRIKLI----CNKI
RTE-1 1p Cel      127 TNFTP------F-HRPVAVPVTKGVRQGDPISPNLFSACLEHVFRK-----LS-WIELKGEAEDYDT---------IP--
RTE_JAM1_1p       127 NCVKI----S-G-EHSSSFESRRGLRQGDGLSCLLFNIALEGVMRR-----AG-L----------N----------SR--
RTE_BovB_1p       127 ATVRT----G-H-GTTDWFQIGKGVRQGCILSPCLFNLYAEYIMRN-----AGLE----------ET---------QA--
RTBmo5            127 GIIRV----E-E-TISAPFKFRKGVRQGCILSPVLFNIYGEYIMRR-----TC-E----------GW---------DG--
RTBmo6            131 GTVRV----D-Y-TMSNRFNFRRGVRQGCILSPILFNIYGEYIMRK-----TL-E----------NW---------DG--
RTBmo3            127 SMVRV----N-D-VISGPFKPEKGVRQGCILSPILFNVYGEYVMRK-----AL-E----------EW---------EG--
RTBmo4            127 AAVRV----D-S-IDSERFSTQAGVRQGCILSPLLFNIYTEYIMRI-----VL-D----------DW---------DK--
RTBmo7            127 ASVRI----D-D-ILSRHFHPNAGVRQGCIISPLLFNIYTELIMRI-----AL-E----------NW---------SD--
```

FIGURE S3 3/5

```
                                                      5                                        6
SLACS 2p            159 --------------------FPEAQFTAYLDDVTVAAPPEE-------LKNVC-AATAEAMEALGIVNNADKTEVLELTG
CZAR                159 --------------------FSNASFTAYLDDVTVAAPPGM-------LGKVC-EATSRAMRALGIETNEDKTEVLNKGG
CRE1                161 --------------------IPGVPVTAYIDDITLAASGAE-------GARAA-EAYADALETVGVVTNARKSMVVGPEG
CRE2                163 --------------------HPRVKVVAYLDDVTLIGPHAA-------VQDFL-AEAGPQLSRVGFDINPAKSHHLAKLE
R1-2_DYa_1p         176 --------------------PDDTFLVGYADDIVAVITARNTEYAQRKLTQVM-TRVKRWLNSHDLKLADEKTELLLVTR
R1 TRAS1            171 ---------------------RGEYIQAFADDVVLVFDGDSALQIERQANTSL-EHVQAWGVRNKLKFAPHKTCAMTITR
LOA Lian-Aa1 1p     173 ---------------------CGFPTYGFADDYLALIVGMCISTLFDLMQSAL-QVVESWCRQYGLSVNPNKTSIVLFTE
LOA_2p              173 ---------------------SGIQCQGYADDIVIMARGKFEESLCDMVQSGL-RITYDWCKEVGLNLNPTKTVIVPFTR
Tad1 Cgt1           172 ----------------------QRLRFGYADDIGLLETSPSLEENTTALSQEI-TQILNWGTDNKVAFEPAKCEAVHFSR
Tad1 2p Neurosp     176 --------------------IPNTITVGFADDTNVVAVARTTEENCRTLQAAW-EVCSGWAGARGMEFEATKTELMHFTR
Jockey_TART         177 --------------------TERIMLSTYADDTIVLSSDTLATAATRNNENYL-KSFSDWADKWGISVNAAKTGHVIFTL
Jockey DOC 2p       169 ---------------------YNLTTSTFADDTAILSRSKCPIKATALLSRHL-TSVERWLADWRISINVQKCKQVTFTL
Jockey_AMY          173 ----------------------PTHLALFADDTTVYYSSRNKSLIAKKLQSAA-LALGQWFRKWRIDINPAKSTAVLFQR
Jockey_JuanA        171 ---------------------QGCQKSLFADDTGLSAKGRSLRVICSRLQKSL-DIFSSYLQKWEISPNASKTQLIIFPH
CR1 T1-ORF2p        169 ----------------------PDGHLLYADDIKIFLPVSSSS-DCMSLQHYL-NAFVHWCSSNLLRLCPDKCSVISFSH
CR1_turtle          171 -----------------------CTLSKFADDTKLGGVVDTLE-GRDRIQKDL-NKLEDWAKRNLMRFNKDKCRVLHLGW
I_Dt_2p             170 --------------------YKEIKFNAYADDFFLIINFKKNT--NISLDNLL-NDIGNWGSYSGASLSLSKCQHLHICK
I DM 2p             170 --------------------HKEIKFNAYADDFFLIINFNKNTNTNFNLDNLF-DDIENWCSYSGASLSLSKCQHLHICR
L1_Xenope           173 -G------LVL-----KEP-DMRVVLSAYADDVILVAQDLV------DLERAQ-ECQEVYAAASSARINWSKSSGLLEGS
L1_Arabidopsis      174 -A------RQF-GYHPKCR-AIGLTHLSFADDLMILSDGKVR-----SIDGIV-KVLYEFAKWSGLKISMEKSTMYLAGV
L1 rat              177 -G------IQI-------G-KEEVKISLFADDMIVYLSDPKS-----STREQL-KLINNFSKVAGYKINSNKSVAFLYTK
L1_Human            177 -G------IQL-------G-KEEVKLSLFADDMIVYLENPIV-----SAQNLL-KLISNFSKVSGYKINVQKSQAFLYTN
L1_Medaka           176 -G------VKV-------S-GKEQKLSLFADDLLLTISQPTK-----TLPIIM-DSLKDFGTLSGYKINVNKIQVLTLNY
L1 chlorella        180 -P------IRL-----PSG-EPAPVMHQHADDTSVHARTPG------MLRSCWGPSVGLHCAATGARLQRSKSQALGLAA
R2_Earwig           170 -G------VNV-------G-SKHYNGLTFADDLLLFATTPE------GLQSSI-DIVHLFLLECGLLINKQKSFVLTVKA
R2_DMer             170 -G------VHV-------G-NAKVNACAFADDLMLFASTPK------GLQELL-NTTVKFLSSVGLTLNADKCFTISIKG
R4 Ancylostoma      184 -E------TRTGAGNRSDG-TLTLGHILYMDDLKIFTPDSG------DMALAE-GGIRRVFGQLGLELNARKCATRSLNC
R4_Oesophagosto     184 -R------TQTGSSVRSEG-VLELGHILYMDDLKIYTPERE------GLEVAL-DGIVGLFGQLGLELNARKCATRCLNS
R4_Ascaris          187 -G------WSA-------GYGFEIGHQFYMDDLKLYARTPA------MLDSQI-QVVSEVSEAMGLHLNLSKCAKAHYAP
R4 Pediculus        178 -E------LVP-------G-GRKITHMLYMDDLKIYAKNEE------ELNKML-RTVQTFSSDINMKFGLEKCARINIVR
REP2_Tth            166 -S------KKL-------D-CQDLHFELFADDMLIILKKYK------LTATLL-EVLKQAYRDINLQINESKTKIMLIGK
REP1_Tth            166 -S------KKL-------D-HQDLHFELFADDMLIILKKYK------LTATLL-EVLKQAYSEINLQINESKTKIMLIGK
REP6 Tth            166 -S------KKL-------D-CQDLHFELFADDMLIILKKYK------LTATLL-EVLKQAYQEINLQINESKTKIMLIGK
RT49653exp          168 -L----------------V-NLFDHYYFYADDLAIILEEKA-------AKQII-EGLKIVSTQLGLIMNFKKCGLLKLHQ
RT49653old          168 -L----------------V-DLFDHYYFYADDLAIILEEKA-------AKQII-EGLKIVSTQLGLIMNFKKCGLLKLHQ
RT43898             168 -L----------------V-DLFDKYYLYADDLAIILKEID-------ATKII-EGLQLLALQLDLKINLKKCGLMKLFS
RT32989             168 -I----------------D-EKFDNYYFYADDLAIICSEQY-------APEVI-KGLELVAKQLSLIINTKKCAIMKLHQ
RT48569exp          168 -D----------------F-SRIDHYYLYADDLAFICKEKY-------LDSTL-ECLNKASQNLNLQINTSKCAIIRITR
RT48569old          168 -N----------------F-SRIDHYYLYADDLAFICKEKY-------LDSTL-ECLNKASQNLNLQINTQKCAIIRITR
RTTth1              168 --------------------QSPIWYKVYADDLVIITQIQH-------LKRVI-QQLRQICLEYKLIINNNKSAVLPLKN
RT43773             171 --------------------GNSFKFLGYADDLVLLTERRN-------LGFLI-SELKRLSTNWNLQINNRKSGIMPIKR
RT43775             171 --------------------GNSFQFLGYADDLVLLTEQGN-------LGFLI-PLIKSLSVQWNLRVNNKKSGIMPMKF
RT48639exp          170 --------------------NDSFQFLGYADDLVVLTEQPN-------LGILL-PQLKNLSSQWNLHINNKKCGIMAIKR
RT48639old          171 --------------------NDSFQFLGYADDLVVLTEQRN-------LGILL-PQLKNLSSQWNLHMNNKKCGIMAIKR
RT3220              171 --------------------SYKVRFLGYADDLVLETEYRN-------VSDLI-QQISILADRWNLVINKKKSGIMPIKF
RT24068             171 --------------------GYAVQTLGYADDLAFILRKCQ-------IQGFV-QILNQLSQEWELHINNTKSGIMPIKY
RT33971             170 --------------------NFNYTSYEFADDIVIIIAHRN-------IQSFL-QQLVLISEEYELRLNQKKCGIFFIQN
RT23387             170 --------------------PFQYNSLEFADDIVIVIEHKY-------TLSFL-QLLAQISEQYELRINKNKCGIFFIQN
RT12275             170 --------------------LFDYESYQFADDLVFFVRQKE-------INSFL-IQIQEISILYNLFINKSKSGIFYIKN
RT46728             170 --------------------QIQYYCFQFADDISSIIHNKY-------TPTYL-QILEEYSMEYNLTIKKKKSGIFLLHN
RT42365             170 --------------------QLQYYSFQFADDIASIIHHKH-------TLKYL-QILEKYSMQYNLSINKKKSGIFLLHN
RT46222             170 --------------------KLNYHSFQFADDIATIIHHKD-------TSKYL-QVLEKFSAQYNLRINKRKCGIFLLHN
RT4057              169 --------------------KFPYQSYQYADDIVFIIKHKF-------ATKLL-QLLELTSSNFDLKINKNKIGIFLLHN
RT46341             170 --------------------NIQYEDYEYADDLCVLIKNKY-------LKSFI-LELSQESVEWNLILNKAKCGIIKLKQ
RT21873             170 --------------------NRELDDYEYADDIVLITKHTN-------TRKVL-AELQMLSQQWNLKINKQKSGIIRLKN
RT10058             171 --------------------QRGLEVQGYADDLIFFVKHQD-------LQQFL-TQLEDVANNWMLKVNKKKSGIFIIKN
RT13985             171 --------------------QRGLEVQGYADDLIFFVKHQD-------LQQFL-TQLEDVANNWMLKVNKKKSGIFIIKN
RT50095             171 --------------------GIAFQSLAYADDVVYFVEHKL-------TQKFV-IKLCELSLAWGLKVNKAKSGIFLFKS
RT31010             173 QLKKNQKNMKR-------W-MSECEILAYADDVVIISRNKV-------AGELA-KIFCESADYHGLVVNVKKTKYLANRE
RT42890             173 QLKKNQKRIKR-------W-MNECEILAYADDIVIISRNKI-------AGEMA-DIFCECADKHGLLVNVKKSKYLANRV
RT33852             173 LLKKKQKRIKR-------W-MKECEILAYADDVVIITRKEI-------AGEMA-QIFSECAEKHGLQVNVKKTKYLANRI
RT38354             173 LLKKKQKRIKR-------W-MQECEILAYADDVVIIARKEI-------AGEMA-QIFCESAEKHGLLVNVKKTKYLANRI
RT4152              173 SLPKNQKRKKR-------W-LEESELLAYADDVVICARREV-------ASICS-EAFIEATEGFKLKTNLKKTKYLANRP
RT47195             173 SLPKNQVKKKK-------Q-LEQSEILAYADDVVIFAHKRV-------AAICA-NTFMGIAEGFKLKTNLKKTKYLANRE
RT36327             173 NLPKKQKRRKR-------W-IEDGEILAYADDVVIYTRKEV-------AKIWA-DAFIQGAKDFELKVNIKKTKYLSNKV
RT25765             173 NLPKKQKRRKR-------W-FQECEILAYADDVVIIARKEV-------AKECA-QFFIERAEHHMLKVNIKKTKYLANRM
RT50144             185 LKKQKENKFQL-------F-LKATKVFAYADDVAITTFDSC-------AGWVV-EQFLDLAEKYGFKVNIKKSNYISNKK
RT41453             185 LKKQKENKFQI-------F-LKATKVFAYADDVAITTFDSC-------AGWVV-EQFLDLAEKYGFKVNVKKSNYISNKK
RT283               185 LKKQKENKFQL-------F-LKATKVFAYADDVAITTFDSC-------AGWIV-EQFLDLAEKYGFKVNVKKSNYISNKK
RT3903              185 LKKQKQNKFQL-------F-LKATKVFAYADDVAITTFDSC-------AGWIV-EQFLDLADKYGFKVNIKKSNYISNKK
RT48784             185 LKKQKQTKFQL-------F-LKATKVFAYADDVAITTFDSC-------AGWIV-DQFLDLADKYGFKVNLKKSNYISNKK
RTE-1 1p Cel        183 -G------MRV-------N-GRNLTNLRFADDIVLIANHPN------IASKML-QELVQKCSEVGLEINTGKTKVLRNRF
RTE_JAM1_1p         173 -G-------TI-------F-TRSGQFVCFADDMDIIGRKFE------TVADLF-TRLKREATRVGLMVNASKTKYMLVGG
RTE_BovB_1p         175 -G------IKI-------A-GRNINNLRYADDTTLMAESEE------ELKSLL-MKVKVESEKVGLKLNIQKTKIMASGP
RTBmo5              174 -G------VTI-------G-GVKLSNLRYADDTTLLAANES------EMAALM-SKLEKISLELGLAINRSKTKVMVIDR
RTBmo6              178 -G------ITI-------G-GVKVTNLRYADDTTLLATTEA------EMTELL-NRMEHIGLEMGLALNRSKTKIMVVDR
RTBmo3              174 -G------ISV-------G-GIKISNLRYADDTTLFASSEK------ELADLF-RRVEYESSLVGLSVNKSKTKVMIVDR
RTBmo4              174 -G------ISV-------G-GRKISNLRYADDTTLLASTRD------EIEVLL-RRLETTALDFGLAINRDKTKMMIVDR
RTBmo7              174 -G------MTI-------G-GRKISNLRYADDITLVASGVS------QMEELL-GRVERVSLDFGLKINRNKTSVMIVDR
```

FIGURE S3 4/5

```
                                                                                7
SLACS 2p         211 -D-------------------------------------------TGF--GTAV-KRVREFLERT
CZAR             211 -P-------------------------------------------VDM-PTEYIRPFARVLGAGV
CRE1             213 -T--------------------------------RV--GI--G-GVDL-P--VV-AEARILGAHF
CRE2             215 VPE-------------------------------AL--SV--S-GRTI-P--IAQGVVRILGAGF
R1-2_DYa_1p      235 -KR----IPL----------E-----I-------DM--RV--G-ENVI-R--TR-KDIKYLGVRL
R1 TRAS1         229 -RL----KYD----------T-----P-------RL--NM--G-GTEI-A--TY-KELRILGLTI
LOA Lian-Aa1 1p  231 -RR----NRD---------GI-----R-------PL--RL--F-GTEI-N--VT-DQVKYVGVIL
LOA_2p           231 -RH----KLQ---------RM-----R-------QI--WL--S-GTPL-E--RS-REVKYLGVIF
Tad1 Cgt1        229 -KH----KQR--------KDL-----P-------DI--QA--R-ELTIKA--ST-KPVRWLGVWF
Tad1 2p Neurosp  235 -TR----APR--------TE--------------TL--QL--G-DTVL-Q--PT-ESTRFLGVWL
Jockey_TART      236 -KN----DLP--------TSL-----R-------TM--KI--K-GQVI-K--IE-SKQSYLGVIL
Jockey DOC 2p    227 -NK----QTC----------------P-------PL--VL--N-NICI-P--QA-DEVTYLGVHL
Jockey_AMY       230 -GS----STRISSRIRRRNLT-----P-------PI--TL--F-RQPI-P--WA-RKVKYLGVTL
Jockey_JuanA     229 -KP----K----ALYLKPSSR-----H-------VV--TM--R-GVPI-N--WS-DEVKYLGLML
CR1 T1-ORF2p     225 -SL----SPI---------S------F-------NY--TL--S-NSSL-S--RV-LSIRDLGIIL
CR1_turtle       226 -KN-----PM----------------H-------SY--RL--G-TDEL-G--SS-SAEKDLGVTV
I_Dt_2p          227 -KH----HC-----------T-----S-------KI--SC--N-NIQI-P--TV-TSLKILGITI
I DM 2p          229 -KR----HC-----------T-----C-------KI--SC--N-NFQI-P--SV-TSLKILGITL
L1_Xenope        233 -LK---VDFL----------------P-------PA---F--R---DISW--ES-KIIKYLGVYL
L1_Arabidopsis   239 -QA----SVY----------------Q-------EI---V--Q-KFSFDV--GK-LPVRYLGLPL
L1 rat           236 -EK----QAE----------------K-------EI---R--E-TTPFII--DP-NNIKYLGVTL
L1_Human         236 -NR----QTE----------------S-------QI---M--S-ELPFTI--AS-KRIKYLGIQL
L1_Medaka        235 -SP------P----------------Q-------NI---K--D-EYKWEW--QA-DSIKYLGIAL
L1 chlorella     241 -SA----ISP----------------G-------PI--QS--R-GVTFAA--SS-DGVKHLGIPL
R2_Earwig        228 -YP----KLK--------------KTAVIVTEKYML---DRHI-LPAI-D--RE-KLFHYLGVPF
R2_DMer          228 -QP----KQK----------------VTVVEQRTFC---IGRA-RVQL-K--RS-EEWKYLGIHF
R4 Ancylostoma   249 -TS----ACS----------------V-------QL---D--E-IPIL-G--AS-EFYKYLGAEQ
R4_Oesophagosto  249 -PG----ASQ----------------V-------QL---D--L-IPSL-G--AT-ELYRYLGAEQ
R4_Ascaris       246 -HGAGGAQEA----------VEGAEGS-------RK---G--E-IPIL-G--LR-STYKYLGVEQ
R4 Pediculus     236 -GK-LKQKQN----------IE---DS-------EE---E--L-IKEL-D--PG-SSYKYLGIEE
REP2_Tth         224 -QE----TYI----------------K----DSLKL---R--Q-AKGI-D--LV-MEFKYLGLVI
REP1_Tth         224 -QE----TYI----------------K----DSLRL---R--Q-AKDI-D--LV-MEFKYLGLVI
REP6 Tth         224 -QE----TYI----------------K----DSIRL---R--Q-AKGI-D--LV-MEFKYLGLVI
RT49653exp       222 -QQ----SKV----------------D-------TN---S--S-ILGI-Q--FV-KSYNYLGIQI
RT49653old       222 -QQ----SKV----------------D-------TN---S--S-ILGI-Q--FV-KSYNYLGIQI
RT43898          222 -QK----QKL----------------D-------EN---S--Q-IQGI-P--FV-KSYKYLGIEI
RT32989          222 -GK----KNF----------------Q--------E---K--V-MNGF-S--FV-RTYRYLGVEI
RT48569exp       222 -RL----MKL----------------E----PQEQK---N--N-LLGI-S--FQ-RSYKYLGVEI
RT48569old       222 -RL----MKL----------------E----PQEQK---N--N-LLGI-S--FQ-RNYKYLGVDI
RTTth1           220 -YQ-----I-----------------D--------S---N--S-IEGI-P--IV-NQYKYLGINI
RT43773          223 -TV-----------------------K--------N---D--S-ICEL-P--IV-YNYKYLGIEI
RT43775          223 -KA-----------------------A--------S---A--S-ICEL-P--VV-YNYKYLGIEI
RT48639exp       222 -KI-----------------------K--------Q---L--S-ICDF-P--IV-QKYKYLGIEI
RT48639old       223 -KI-----------------------K--------Q---L--S-ICDF-P--IV-QKYKYLGIEI
RT3220           223 -II-----------------------Q--------E---E--Q-CEGF-P--IL-YSYKYLGIQI
RT24068          223 -KM-----------------------K--------Q---L--E-MNDL-P--II-YKYRYLGITM
RT33971          222 -HK----RC-----------------Q--------Y---N--N-IQGF-P--IV-KSYKYLGINI
RT23387          222 -HK----YC-----------------K--------Q---D--N-LNGF-P--IV-KNYKYLGVNI
RT12275          222 -HS----VPN----------------N--------I---K--Q-IQDF-P--IV-KHYKYLGITI
RT46728          222 -HS----K---------------------------L---I--Q-IMSY-P--IV-KEYKYLGLLI
RT42365          222 -HS----K---------------------------L---V--E-VKLY-P--VV-KEYKYLGLLI
RT46222          222 -HS----Q---------------------------L---S--E-IMNY-P--VV-QEYKYLGLLI
RT4057           221 -HS----S------------------N--------I---Q--N-LLQF-P--IV-KEYKYLGILI
RT46341          222 -QQ----E------------------K--------Q---K--Y-IEGI-P--IV-DQYTYLGISM
RT21873          222 -QV----K---------------------------T---G--D-ILGF-P--IV-TKYVYLGITV
RT10058          223 -HL----QK-----------------K--------P---I--S-IFDY-P--VV-KQYVYLGVMI
RT13985          223 -HL----QK-----------------K--------P---I--S-IFDY-P--VV-KQYVYLGVMI
RT50095          223 -IK----E------------------K--------G---N--A-IEGI-P--IV-REYKYLGVAL
RT31010          237 -IK--------------------------------D---L--E-LMKV-Q--KV-ESFRYLGTWL
RT42890          237 -IE--------------------------------D---L--E-LKKI-E--RV-ESFRYLGTWL
RT33852          237 -NE--------------------------------D---L--E-LKKI-E--RV-ESFRYLGTWL
RT38354          237 -NE--------------------------------D---L--E-TKKI-E--RV-ESFRYLGTWL
RT4152           237 -IQ--------------------------------G---L--D-DMQV-E--KV-KNFRYLGVWL
RT47195          237 -VK--------------------------------E---M--D-ELQI-E--KV-KTFRYLGVWL
RT36327          237 -IE--------------------------------P---L--E-QIQI-E--KV-QNFRYLGTWL
RT25765          237 -NE--------------------------------G---L--E-ELKI-E--RV-ENFRYLGTWL
RT50144          249 -SN----H------------------Q--------D---L--V-KLGI-L--KV-KNFKYLGVYI
RT41453          249 -SN----H------------------Q--------D---L--V-KLGI-L--KV-KNFKYLGVYI
RT283            249 -SN----H------------------Q--------D---L--V-KLGI-L--KV-KHFKYLGVYI
RT3903           249 -SN----H------------------Q--------D---L--V-KLGI-L--KV-KHFKYLGVFI
RT48784          249 -SN----H------------------E--------E---L--V-KLGI-F--KV-KHFKYLGVYI
RTE-1 1p Cel     241 -AD----PSK----------------V-------YFGSPS--P-TTQL-D--DV-DEYIYLGRQI
RTE_JAM1_1p      230 -TE----RDR----------------TRL-GSSVTI---D----GDTF-E--VV-DEFVYLGSLL
RTE_BovB_1p      233 -IT----SW-------------------------EI---D----GETV-E--TV-SDFIFLGSKI
RTBmo5           232 -MN----KL-------------------------EH---T--G-SLHL-E--TT-ERFIYLGSMI
RTBmo6           236 -TK----KL-------------------------EL---S--G-TLNL-E--LV-DNFIYLGSNI
RTBmo3           232 -TS----QL-------------------------SR---T--GELSDL-E--FV-SEFIYLGSLL
RTBmo4           232 -AN----INQ----------------P-------EV---Q--H-IAGC-E--VV-NSYVYLGSTI
RTBmo7           232 -AN----NNS----------------P-------EV---T--K-IANC-D--VV-QSYIYLGALI
```

FIGURE S3 5/5

```
Tennessee    1 MNEQQVSSPNLKLQNYSFINIQIFSHQLLQVITIEEANQIVGSMKSLYTGLILKGYYLPKFESACITSEYLLNVALKKVF
DDE1         1 ---MYFTLKKITNIFKLNRYIIFMIYQF-QKVTIEEANRIVGSLKSMYTGLTIKGYYLPQINSPCVTSDYLLGVALKKFF
DDE2         1 -MDQVKIILSVKITSPAIYNTQIIIFQY-QVITIDEANRLLGSLKSVYTGLILKGYYLPKFNSPCITSSYLLGVTLQRLF
NFA          1 ------------------MNQQIAEGVP-LLISRVDANQLIPSIESAYIGLQIQGKYLPKITSSIITSNYLLKVLFDEYY
NFB          1 ------------------MNQQIAEEVP-LLISRVDANQLIPSIESAYIGLQIQGKYLPKITSSIITSNYLLKVLFDEYY
Merou        1 -------------------MNQIQEEEL-IALSRQQANQLIPSLESAYYGLVLQGKYLPKLNSSIITSEYLLGVLFETYY
Coelo        1 -------------------MNQIQEEEF-VALSRQQANQLIPSLESAYYGLVLQGKYLPKLNSSIITSEYLLGVLFDTYY
Sardine      1 ------------------MQNIFQEEENEFKISRADANQLIPSLESAYYGLLIQGKYLPKLTSSIITSEYLLGVLFEKIY
ThonC        1 ------------------MQNNLQEEEY-MKLTRQDANQLIPSIESAYYGLLIQGKYLPKLTSSIITSDYLLGVLFDRIY
ThonA        1 ------------------MQNIFQEEEY-MKLTRQDANQLIPSLESAYFGLLMQGKYLPKMTSSIITSEYLLGILFDKIY
ThonB        1 ------------------MQNILQEEEY-MKLTRQDANQLIPSLESAYFGLLMQGKYLPKMTSSIITSEYLLGILFDKIY
DDE3         1 ---MVMKNNDDLSYAYQNYQNQFTLLFQ-AYVTIQEGNAAVGSIESMYTALIKKGIYLPEMNSAAVTSQYLLNVALGKCF
AnchoisA     1 ------------------------------MISIFQANQATGSVESMYYGLILAGYYLPAIHSSGVTAQYLLKVATKQCF
AnchoisB     1 ------------------------------MISILQANQATGSVESMYYGLILAGYYLPAIHSSGVTAQYLLRVATKKCY

Tennessee   81 VCKLKDIQIAYLIKRATKLDLYRELCQI-IGQEIDLGFTSEKMPDRQWLINILFHVSEQHPYFLQPESN---IFKGLNQT
DDE1        77 SQKQADIKIAYLIRKATKIDLYRELSQI-LGQEQDLGFTSEKLPNREWLINVLYKISPNHRYFQEPSSN---LLAGMSQQ
DDE2        79 HCKQQDIKIAYLIKRATKIDIYREIFQI-IGQEIELGFTSDKLPDREWLINVLYFISPNHRYYQEPTS---FRLSGLSQQ
NFA         62 VPETEDVKIGVLLRPVKKQDVIFELMKVKLNQNQSWGIDEKYQPDKQWAANVLKSIKPDSDLFKKYIPQ-KFDMKRFTLE
NFB         62 VPETEDVRIGVLLRRVKKQDVIFELMKVKLNQNQSWGIDEKHQPDKQWAVNVLKSIKPDSDIFKKYSPQ-KFDMKQFTSE
Merou       61 VPKIDEINIGVLLKPIKKLELIEELLKIQINGQK-WGIDVKHTPNKEWIVNVLKTLKPGHCVFKTQSDIAKFDMRKFTSE
Coelo       61 VPQVEDINIGVLLKPIKKLELIDELIKIQIQGQK-WGIDLKHTPNKEWIVKVLKTLKPDHAIFKSQTEIAQFDMKKFSIE
Sardine     63 VPNLSEVQIGVLLKAVKKYEIIEEVQKIPIQQKT-WGIDQKHQPDKDWIINVLKTLKPNHSFFQKYKTDSKYDMKIFTQD
ThonC       62 VPKLEEVQIGVLLKSVTKYELIQEILKIPIQQQI-WGIDQKRTPDKDWIINVLKTLKPNHVFFQKYKQESKLEMELFTQE
ThonA       62 APKLDEVQIGVLLKSVTKHDLIQELIKIPIQQQI-WGIESKRTPDQEWIINVLKTLKPNHIFFQKYKQESKYDMKLFTLE
ThonB       62 APKLDEVQIGVLLKSVTKHELIQELIKIPIQQQI-WGIESKRTPDKDWIINVLKTLKPNHIFFQKYKQESNYDMKLFTLE
DDE3        77 RLDQQNIRQGQLFKKVSKLKLVQQLEKWCNQQQQILGIEEDKLPDKKWLINILYTVNQENEIFQSYQDSDR--IIGLTRD
AnchoisA    51 GLESKKVQIGQLLRSATKEEILHEIKKKLNGQEL--GFSDGNYPDKTWAVNVLKTIEPNHSLFKNPKVQ---DQSNLTQD
AnchoisB    51 SLESKKVQIGQLLRSATKEEILHEIQKKLNGQEL--GFADGNYPDKTWAVNILKTIDPNHSLFKNPKVQ---DQSNLTQD

      Intron-containing codons
Tennessee  157 DFLKLKELNTSMFRSKRL-RFFKTQPEQRIQRQANLIKSRIQKIKTQFEEETKRQTDRAGKIEQLKQSLKQLLPNQTFRQ
DDE1       153 DIQKLKELNTSLFRTKRL-RFFKTTPNQRLEKRQQKLKDQMNKLKTQLEEDKMNQTNRIGKLQLLDTQLKELKAIQQENQ
DDE2       155 DIQKLKELNTSLFRKKKL-RFFKSTLEQRVLKKSLKIKDKMYNLTAQLQEEKKKQTNIIGKLQHLNTQLQEISQIEKQEN
NFA        141 QMEKIQYLDVTQGKKGSVQRFFRVPKDKQNDIKLERQLLFKKELEKKIERKKQSLVWSQKEILEMGEKMQQI--------
NFB        141 QMEKIKSLDITQGKKGSVRRFFRVPKDKKNDIKLEKQLQLKKELEKKIERKKQSLVWTQKEILEMGEKMQQI--------
Merou      140 QVAKIKELDFSMGKKSNVRRFFRVSKDKQLELYKQKQIIKKQNLLQKMKRKKSQIDEYNKDLDTIQEKVTNIQNI-----
Coelo      140 QINKIKELDLSMGRKSNVRSFFKISKEKQIELEKQRQFIKKKALLQKMSRKKSQIEEFNKEIVSIHEKVSNIQPN-----
Sardine    142 QIKKIKELDFSMGKKTNVKRFFRISKDKQMEADKQKKILKKKTLESKMDRKLKIIDDYEKELKLIENKIKDIEGE-----
ThonC      141 QYNKIKELDLSMGKKSSVKRFFRISKDKQQEARQLKKNLKKKNLEFKIECKNKLIEECERDIPQFENRIKDIDHS-----
ThonA      141 QYNKIKELDLSMGKKSSVKRFFRISKEKQQEAEQFRKSLKKKNLEVKIDRKHKLIQECEKDIQVIEDRIKDIAGN-----
ThonB      141 QYNKIKELDLSMGKKTSVKRFFRISKEKQQEAEQFRKNLKKKNLEVKMDRKHKLIEECEKDIQFIEDRIKDITGN-----
DDE3       155 ELNILKQLDTSLGRTKKM-NFFKRNPEQIRQRKQNLAKQKLSNTKSRLDKKKKEAEKIEEKIKEYQIQLSGPVNQSVIRQ
AnchoisA   126 DLMLIRQLDTSIGRNKRL-RYFRRTKEQVEIFKQNAAKKKLARKELIQAKKLLKLSKLTEELEIVNQGIQEEVEQRQQFQ
AnchoisB   126 DLILIRQLDTSIGRNKKL-RYFRRTKEQVEKFKQDAAKKKQARKELIQAKKLLKLSKLTEELEQINQGIQEEVQQAQSFQ

Tennessee  236 NQNQGQVQFMQNFELQEESQ-SQ
DDE1       232 QQNQGSQAFRENFNLQEELF-SE
DDE2       234 NQNLRSQAFQSNFNLQEEVVQSE
NFA            -----------------------
NFB            -----------------------
Merou          -----------------------
Coelo          -----------------------
Sardine        -----------------------
ThonC          -----------------------
ThonA          -----------------------
ThonB          -----------------------
DDE3       234 EEMAIKKQQRL------------
AnchoisA   205 RRN--------------------
AnchoisB   205 RRN--------------------
```

FIGURE S4 1/1

```
                                *
IS630Sd        1 HPVFY-QDEVDIDLN-PK-I----GADWMPKG---Q-QKRI-----------ATP-GQNQ-KHY-LAG-----ALHS-G-
IS630Ss        1 HPVFY-EDEVDIHLN-PK-I----GADWQLRG---Q-QKRV-----------VTP-GQNE-KYY-LAG-----ALHS-G-
Tigger1        1 QQIFN-VDETAFYWK-KM-P----SRTFIARE---E-KSMP-----------GFK-ASK--DRLTLLL-----GA-N-A-
pogoDm         1 EDIFN-ADETALFYK-AM-P----NATFFTCG---K--QLN-----------GQK-SQR--VRLTLLF-----IC-N-A-
Fot1           1 ERRYN-ADEGGIMEG-QG-V----NGLVIGSSQESP-NAVP----------VKTA-TVR--TWTSIIE-----CI-S-A-
Flipper        1 ENRWN-MDEAGIIEG-QG-L----NGMVLG--SSKR-RFIQ----------KKQP-GSR--TWTSFIE-----CI-S-A-
Tan1           1 DDIYN-FDETGFAMG-MI-A----TYKVV-TSSQRA-GRPS----------LVQP-GNR--EWVTAIE-----CI-R-S-
Parker         1 TGIVF-LDEVGFNLS-MR-T----SQGRSQKG---T-IPTL-----------VVP-QLRS-RNISIIC-----AM-D-K-
Mango          1 SGLIY-LDEVGFNVS-MR-T----SKGRSQKG---T-PAVT-----------VVP-QIRT-RNISIVC-----AM-N-S-
Kiwi           1 DAIIF-IDEVGFQVN-MR-V----AYGRSPVG---E-RAVK-----------VVP-ALRS-RNISIVC-----AM-T-R-
Whistler       1 YEVIF-IDEVGFNVS-MR-D----TRGRALAG---K-PAVK-----------ELP-ALRA-RNISVVC-----AM-S-R-
ThonC          1 YKLIF-IDECSLGNISKS-T----HKQWHVLG---S-FKES-----------TQR---IS-SFKYLIG-----SL-G-D-
Coelocanth     1 YKLIF-IDECQLGNISKS-S----HKQWHVVG---S-FKQT-----------SHR---IS-SFKYLIG-----AL-G-F-
Merou          1 YKIIF-IDECSLGNISKC-S----HKQWHILG---S-FKQM-----------THR---VS-SFKYLIG-----AL-G-F-
Sardine        1 YKLIF-IDECSLGNISKA-S----HKQWHVVG---T-FKEI-----------THR---IS-NFKYLIG-----SL-G-D-
ThonB          1 YKIVF-IDECSLGNISKS-A----HKQWHVLG---T-FKES-----------TQR---LS-SFKYFIG-----AL-G-E-
ThonA          1 YKIVF-IDECSLGNISKS-A----HKQWHILG---T-FKES-----------TQR---LS-SFKYLIG-----PL-G-E-
Baudroie       1 QKMIF-IDECSIGNISKQ-N----HKQWHQQG---S-FKQS-----------FER---NR-LLLTLIA-----AI-G-D-
TEC2           1 YTIVY-IDECSFNAS-AL-P----LYTWNKIG---D-EPVK-----------LIR-STNQ-RFNCIAA-----QV-E-Q-
TEC1           1 FTVVY-IDECSFNRS-AL-P----LYTWHAKG---T-EAPK-----------LIR-SSNQ-RYNCIAA-----QV-C-N-
DDE2           1 YQIIY-IDETTFSHQ-SH-N----PKCWQSKK---Q-QKFR-----------RLT--NHK-QHLNVIG-----GI-G-N-
DDE1           1 FKTIY-IDESTFAHQ-SR-N----PKCWQQKK---N-QKFR-----------KLV--NLQ-KSVNVMG-----GI-G-Q-
Tennessee      1 YNLLF-LDETTFTHH-SK-N----PKCWQLKE---D-IKYR-----------RLI--QLK-KPLHILG-----CI-G-Q-
DDE3           1 TQFIF-LDETTFGHS-HK-S----IKCWQLKG---Q-SKTQ-----------IYS--KRD-KRISVLG-----GI-G-T-
AnchoisA       1 QKIVY-IDECSFGRN-LK-V----ARGWQKKN---T-FPLL-----------QIK-SSSS-KNKCVIG-----AI-A-C-
AnchoisB       1 QKLVY-IDECSFGRN-LK-A----GRCWQKKN---S-YPLL-----------QIK-SNSS-KSKCVIG-----AI-C-C-
TBE1           1 IKFIH-ADEAVFTFS-TF-I----QKSWYKRN---S-NIEV-----------YDQ-KVKV-QTMAILG-----GI-S-E-
Mbmar1         1 HRIVT-GEEKWIFFV-NPKR----KKSYVDPG---Q-PATS-----------TARPNRFG-KKTRLCV-----WW-D-Q-
Dmmar1         1 HRIVT-GDEKWIFFV-NPKR----KKSYVDPG---Q-PATS-----------TARPNRFG-KKTMLCV-----WW-D-Q-
Botmar1        1 HRIVT-GDEKWIYFE-NPKR----KRSWVAPG---E-PPTS-----------TTRPNRYG-KKAMLCV-----WW-D-Q-
Momar1         1 YRIIT-SDEKWIYYD-NPGR----KRSWVSPG---E-PAEK-----------SVRRNRFG-KKTMLCV-----WW-D-Q-
Sinvmar1       1 HRFVT-GDEKWIYFD-NPKR----KKSWVDPG---Q-PSVS-----------TPKRNIHE-HKTLLCV-----WW-D-Q-
Funmar         1 NRIIT-CDEKWILYD-NRKR----SSQWLNPG---E-PAKS-----------CPKRKLTQ-KKLLVSV-----WW-T-S-
BmMLE          1 NRINT-CDEKGVLYD-NRKR----LSQWLNPG---D-PAKS-----------YPKRKLTQ-KKLLVSV-----WW-TSI-
Planmar8       1 HRIVT-CDEKWIMYD-NRKR----SSQWLDPD---E-PPKH-----------CPKRKVHQ-KKLMVTV-----WW-S-S-
Avmar1         1 SNLVT-GEEKWVLYV-NYTR----RRQWLSAG---Q-TGVA-----------TPKADLHP-KKLMISV-----WW-G-I-
Cemar2         1 NNLVT-GDEKWVLYV-NHSR----KRQWLPIG---E-KGIP-----------TPKPDLHP-KKIMICV-----WW-G-V-
Cbmar1         1 DRLITGVDEKWVFYD-NHHR----KSQWVGEG---E-SPQD-----------VPKPDLHP-KKVMISV-----WW-G-V-
Mcmar1         1 DQIIT-CDESWILYD-NRAS----EKQWLAVG---Q-DANA-----------TPK-QLHP-KKQLISV-----WW-C-V-
Ccmar1         1 DRLLT-GDEKWILYN-NVQR----KRTWKQAH---E-GAEP-----------MSKGGLHP-MMVLLCI-----WW-D-I-
Acmar1         1 KRIVT-GDEKWIVYD-NIKR----EL-----N---E-PAQR-----------TSKNNIH--KKVMISV-----WW-D-F-
Ammar1         1 KRLIT-GDEKWVVYN-NIKR----KRSWSRPR---E-PAQT-----------TSKAGIHR-KKVLLSV-----WW-D-Y-
Famar1         1 KRLIT-GDEKWVVYN-NIKR----KRSWSRPG---E-PAQT-----------TSKAGIHQ-KKVLLSV-----WW-D-Y-
Ccmar2         1 KRLIT-GDEKWVVYN-NIKR----KRSWSRPG---E-PTQT-----------TSKADIHQ-KKVLLSV-----WW-D-Y-
Camar1         1 KRIIT-GDEKWIVYN-NVNR----KRSWSKHG---E-PAQT-----------TSKADIHQ-KKVMLSV-----WW-D-W-
Himar1         1 RRYVT-MDETWLHHY-TPES-NRQSAEWTATG---E-PSPK-----------RGKTQKSA-GKVMASV-----FW-D-A-
Bytmar1        1 SRVMT-GDETWVHHW-DPET-KLESMAWKQKG---S-PTPL-----------KFWTQPLA-GKIMATI-----FW-D-A-
Hsmar2         1 RRIVT-GDETWLYQY-DPED-KAQSKQWLPRG---G-SGPV-----------KAKADWSR-AKVMATV-----FW-D-A-
Tvmar1         1 RFLVT-GDESWFQYA-TE-A----KIMWIPKD---E-NPQT-----------FPKKKIDT-PMMMISV-----FW-G-V-
Psmar1B        1 HNIIH-IDEKWFYMT-KK-S----EKYYLLPD---EDEPYR-----------TCKSKNFI-AKVMFLVAQTRPRF-D-SE
Soymar1        1 YNIIH-IDEKWFYMT-KK-S----ERYYLLPD---EDKPHR-----------SCKSKNFV-PKVMFLTAVARPRF-D-SE
Vulmar1        1 ENIIH-IDEKWFYLN-PD-T----RRFYLLPR---EDDPYR-----------CCQSKRFK-IKAMFIGVVSKPMY-D-AQ
Aee37E1        1 GCIIM-DDETYVKAD-FG--QIPGQNFYLATA---RGD-VPAK-------FKFVFADKFA-RKYMIWQ-----GI-C-S-
Aea37E1        1 GCIIM-DDETYVKAE-FG--QIPGQKIYLATA---RGD-VSAK-------FKFVFADKFA-RKNIIWQ-----GI-C-S-
Aet37E1        1 GCLLM-DDETYVKAD-FG--QIPGQKCYLATA---RGD-VPA-----------KFKFVFA-DKFAIWH-----GI-C-S-
Ag37E1         1 GCLLM-DDETYVKAD-FG--QIPGQTFYLATG---RGD-VPAK-------FKFVFADKFA-RKFMIWQ-----GI-C-S-
CbmaT5         1 SEIIW-TDEKIFTVE-PL-PNRQNARQLLSKD---E-AKSP-KR--------RLAYKRLFP-KSVMVWA-----GL-T-S-
CbmaT4         1 SDVIW-TDEKIFTIE-PL-PNRQNQRQLLSKD---D-SMSP-KR--------RLAHNRLFP-KSVMVWA-----GI-T-A-
CemaT1         1 RKVLF-TDEKIFCIE-QS-FNTQNDRVYAKTQ---P-------NS--------RVQRTGYP-KGIMVFA-----GI-T-A-
CbmaT1         1 LITIF-SDEKLFSVE-AE-FNSQNHRVLATNI---Q-QAAE-RG--------KTIHRASHP-ASIMVFG-----AV-C-Y-
CemaT3         1 LKMLF-TDEKLFKVE-AE-FNPQNHRVLASTS---E-QANN-QG--------RMIKRASHP-QQVMVFG-----AI-T-S-
CbmaT3         1 MNMMF-VNEKLLTVE-VE-FSLKNYLVSSPRS---E-----------LSNEHSNMKKVYH-QNVMVFT-----TT-S---
Bmmar6         1 REILF-SDEKIFTVE-ES-YNKQNDKVYAHSS---E-EASN-RI--------PRVQRGHFP-SSLMVWL-----GV-S-Y-
Bmmar1         1 RKILF-TDENFFTIE-QH-FNKQNDRIYAQSS---K-EASQ-LV--------DRVQRGHYP-TSVMVWW-----GI-S-Y-
MdmaT1a        1 PNLVF-SDEKKFVVQ-QF-VNKQNDGVYAPK------------RTCENGHLKLCTRGWPPEAMVMVWA-----AI-T-A-
Bari1          1 FNILW-TDESAFQYQ-GS-YSK--HFMHLKNN---Q---KH-LA--------AQPTNRFGG-GTVMFWG-----CL-S-Y-
Maya           1 KNVAW-SDESRFLLR-HS-NGR--VRIWRKQN---E-NMDP-SC--------LVTTVQAGG-GGVMVWG-----MF-S-W-
Tc3            1 SKVVF-SDEKKFNLD-GP-DGC--RYYWRDLR---K---EP--M--------VFSRRNFGG-GTVMVWG-----AF-T-E-
S              1 DDVIF-CDETKMMLF-YN-DGP--SRVWRKPL---S-ALET-QN--------IIPTIKFGK-LSVMIWG-----CI-S-S-
Paris          1 DDVIF-CDETKIMLY-YH-DGP--SKVWRKPN---T-ALEQ-KN--------IIPTVKFGK-LSVMVWG-----CI-S-S-
Quetzal        1 KKVLF-TDESKFNIF-GW-DGT--IKVWRPPG---E-GLNP-KY--------TAKTVKHNG-GGVLVWG-----CM-A-A-
Tcb2           1 ANHVF-SDESKFNLF-GT-DGI--KWIRRPVG---C-RFDP-SY--------QLQTVKHGG-GSVMVWG-----CF-S-G-
Tc1            1 AKHIW-SDESKFNLF-GS-DGN--SWVRRPVG---S-RYSP-KY--------QCPTVKHGG-GSVMVWG-----CF-T-S-
Tcb1           1 RKVIW-SDESKFLLF-GT-DGI--KFVRRPVG---T-RYHP-SY--------QLPTVKGGG-DSVMVHG-----SF-C-G-
Minos          1 DTIIF-SDEAKFDVS-VG-DTR--KRVIRKRS---E-TYHK-DC--------LKRTTKF-P-ASTMVWG-----CM-S-A-
Impala         1 RRVKW-SDECMVRRG-QG-MRP--IWTFLSPR---E-ALRV-QD--------VQEARRLGA-VRQMFWA-----AF-G-H-
```

FIGURE S5 1/4

```
IS630Sd     49 --------TGRV-HYV----------------------SGSSK--------------------------SSDLFIS-L-L
IS630Ss     49 --------TGKV-SCV----------------------GGNSK--------------------------SSALFIS-L-L
Tigger1     48 --------AGDFKLKPML------------------IYHS---ENPRALKNYAKSTLPVLYKWNNKAWMTAHLFTA-W-F
pogoDm      47 --------TGTY-KKTFV------------------IGRS---KS---PRCFKNANVPIPYYANKKAWMTKDLWRK-I-M
Fot1        52 --------VGVV-LHPLV------------------IFKAKTIQEQWFRREFLQKHLGWQVTFSKNGWTSNSIALE-W-L
Flipper     50 --------TGRA-LLPLV------------------IFKGKTLQQQWFP-IKLDNYEGWEFTATDNGWTTDSTGLE-W-L
Tan1        51 --------NGEV-LPSTL------------------IFKGKTHLKAWYE--GQSIPPTWRFEVSDNGWTTDKIGLR-W-L
Parker      49 --------NGIV-HYHS---------------------HNRAV--------------------------NRELFKQ-F-I
Mango       49 --------NGIV-HYVT---------------------HNQPV--------------------------NRELFTN-F-I
Kiwi        49 --------KGIT-HYQH---------------------RNSAI--------------------------NKVFFIE-F-L
Whistler    49 --------NGIV-HYVS---------------------RTRAI--------------------------NKEFFVS-F-I
ThonC       48 --------DNFL-WYQI---------------------FSGTG--------------------------KAYIFQD-F-L
Coelocanth  48 --------DNFL-FFQL---------------------FEGTG--------------------------KAFIFQD-F-I
Merou       48 --------NGFL-FFQL---------------------FEGTG--------------------------KAYIFQD-F-V
Sardine     48 --------DCFL-QYQI---------------------FSGTG--------------------------KGFIFRD-F-L
ThonB       48 --------DNFL-QYQL---------------------FSGTG--------------------------KAYIFQD-F-L
ThonA       48 --------DNFL-QYQL---------------------FSGTG--------------------------KAYIFQD-F-L
Baudroie    48 --------NNFL-CFQL---------------------IEETG--------------------------KSHIFKH-F-C
TEC2        49 --------HKI---FHI---------------------KTETT--------------------------KDQNFIT-F-L
TEC1        49 --------HKL---FHV---------------------KQDTT--------------------------KEDSFIE-F-L
DDE2        48 --------NGFQ-FFQI---------------------VLGNV--------------------------NQYVFGQ-F-I
DDE1        48 --------NGFQ-IFQI---------------------CIGNV--------------------------NQFVFGQ-F-L
Tennessee   48 --------NGFG-CFQI---------------------CVGHV--------------------------NQYVFAQ-Y-F
DDE3        48 --------QGFV-AYQL---------------------IIGNL--------------------------NSHFYLF-H-L
AnchoisA    49 --------DGFI-AYKC---------------------VIGNV--------------------------NQQVFGE-F-L
AnchoisB    49 --------DGFI-AYKC---------------------VIGNV--------------------------NQQVFGE-F-L
TBE1        49 -------DAGLE-TYII---------------------HPRSI--------------------------KTEQYIK-F-L
Mbmar1      51 --------SGVI-YYELL-------------------KPGETV--------------------------NTARYQQ-Q-L
Dmmar1      51 --------SGVI-YYELL-------------------KPGETV--------------------------NTARYQQ-Q-L
Botmar1     51 --------KSVI-HYELL-------------------KPGETI--------------------------NTERYQQ-Q-M
Momar1      51 --------RGVI-YHELL-------------------KPGETV--------------------------DTARYQQ-Q-L
Sinvmar1    51 --------KGVL-YYKLL-------------------GPNKTV--------------------------TADCYHQ-Q-L
Funmar      51 --------AGVV-HYSFL-------------------KSGLTI--------------------------TADVYCQ-Q-L
BmMLE       52 --------ASVV-HYSFL-------------------KSGQTI--------------------------TADIYCQ-Q-L
Planmar8    51 --------YGVI-HYDFM-------------------VPGTSI--------------------------TSDVYCS-Q-L
Avmar1      51 --------KGVI-HWEVL-------------------PNGYTI--------------------------TADLYCQ-Q-L
Cemar2      51 --------QGPV-HWELL-------------------PTNKTI--------------------------TADYYCA-Q-L
Cbmar1      52 --------DGPI-YWELL-------------------PEGKTI--------------------------TGDLYTT-Q-L
Mcmar1      50 --------HGIV-YWELL-------------------PLNRTI--------------------------TSEVYCE-Q-L
Ccmar1      51 --------RGVI-YFELL-------------------PAGETI--------------------------TANKYCQ-Q-L
Acmar1      45 --------KGVV-FFELL-------------------PNNCTI--------------------------NSEVYCN-Q-L
Ammar1      51 --------KGIV-YFELL-------------------PPNRTI--------------------------NSVVYIE-Q-L
Famar1      51 --------KGIV-YFELL-------------------PPNRTI--------------------------NSVVYIE-Q-L
Ccmar2      51 --------KGIV-YFELL-------------------PPNRTI--------------------------NSDVYIE-Q-L
Camar1      51 --------KGVV-YFELL-------------------PRNQTI--------------------------NSDVYCQ-Q-L
Himar1      54 --------HGII-FIDYL-------------------EKGKTI--------------------------NSDYYMA-L-L
Bytmar1     54 --------GGVL-LVDVL-------------------PRGSTI--------------------------TGKYYAG-V-L
Hsmar2      54 --------QGIL-LVDFL-------------------EGQRTI--------------------------TSAYYES-V-L
Tvmar1      50 --------NGII-AIDIL-------------------QKPNTM--------------------------NAQYLIDNV-L
Psmar1B     57 ENETFSGKIGVF-PF--VTHEPAIRSSINRVAGTMVTKAITTV--------------------------NRDVVRS-FLI
Soymar1     57 KNVTFSGKIGIF-LF--VTQEPAKRTSVNRVAGTMETKAITSI--------------------------NRDLIRS-VFI
Vulmar1     57 GNLIHDGKFGIF-PF--VERVAAKYTTKNRDKGTIETKVVERI--------------------------TQKVIRD-MII
Aee37E1     57 --------CGQK-TKVFL-------------------T-DKTM--------------------------TSEVYKKEC-L
Aea37E1     57 --------CGQK-TKVFL-------------------T-DKTM--------------------------TSEVYKKEC-L
Aet37E1     53 --------CGRK-T-VFV-------------------T-NKTM--------------------------TSELYQKEC-L
Ag37E1      57 --------CGKK-TKVFV-------------------T-NKTM--------------------------TSELYQKEC-L
CbmaT5      57 --------EGKV-PLVFI-------------------DRNVKI--------------------------NSDVYQKLV-L
CbmaT4      57 --------TGKT-PLVFI-------------------ERNVKI--------------------------NSEVYQKIV-L
CemaT1      52 --------NGKT-PLIFV-------------------PQGIKV--------------------------NGNNYLD-M-L
CbmaT1      57 --------DGKC-PLVFV-------------------DKGVKI--------------------------NQKYYVEEI-L
CemaT3      57 --------DGKM-PLIFV-------------------DPGVKI--------------------------NKEYYLEET-L
CbmaT3      54 -------------KLIFV-------------------DFGVKI--------------------------NRNDYAE-I-F
Bmmar6      57 --------WGLT-EVHFC-------------------EKGVKT--------------------------NAVVYQNTV-L
Bmmar1      57 --------EGVT-EPYFC-------------------EKGIKT--------------------------SAQVYQDTI-L
MdmaT1a     58 --------NGRS-PLVLI-------------------DRGIRI--------------------------NAEYYCENI-L
Bari1       53 --------YGFG-DLVPI-------------------E--GTL--------------------------NQNGYLL-I-L
Maya        55 --------HTLG-PLVPI-------------------G--HRL--------------------------NATAYLS-I-V
Tc3         52 --------KKKL-EIQFV-------------------S--SKM--------------------------NSTDYQN-V-L
S           55 --------HGVG-KLAFI-------------------E--STM--------------------------NAVQYLD-I-L
Paris       55 --------KGVG-ELRIF-------------------N--DVM--------------------------TKEFYLD-I-L
Quetzal     55 --------NGVG-NLQVI-------------------D--GIM--------------------------DQYVYIN-I-L
Tcb2        55 --------TSMD-PLRRI-------------------D--SIM--------------------------DRFVYED-I-L
Tc1         55 --------TSMG-PLRRI-------------------Q--SIM--------------------------DRFQYEN-I-F
Tcb1        55 --------KGAG-PLHRI-------------------E--GKM--------------------------DAKMYLN-I-M
Minos       54 --------KGLG-KLHFI-------------------E--GTV--------------------------NAEKYIN-I-L
Impala      55 --------RSRT-PLVPL-------------------V--GNV--------------------------NAIGIYE-L-Y
```

FIGURE S5 2/4

```
                                                                   *
IS630Sd      70 ETLRRTY----R-------------------RAKTITLVADNYIIHKSRKVERW-----LEE--NPKFR---LLFLPMYS
IS630Ss      70 KRLKATY----R-------------------RAKTITLIVDNYIIHKSRETQSW-----LKE--NPKFR---VIYQPVYS
Tigger1      97 TEYFKPT---VE-------TYCSEK----K-ISFKILLLIDNAPGHPRAL-MEM-----Y----K-EIN---VVFMPANT
pogoDm       92 TGF-D------E-------E-M--K----K-QNRKILLFIDNATSHTTVK---D-----F----E-NIK---LCFMPPNA
Fot1        103 EKVFLPQ---TA-------P----A----D-PADARLLIVDGHGSHATEQFMAK-----CYL--N-NVY---LLFLPAHC
Flipper     100 KEVFIPQ---SA-------P----T----R-PKEARLLVLDGHGSHETTQFMLE-----CFK--N-NIH---LLFLPPHT
Tan1        100 QKHFIPL---IR-------G----K----S-VGKYSLLVLDGHGSHLTPEFDQS-----CAE--N-EVI---PICMPAHS
Parker       71 LQLKEKL----R-------T----R------GIDESYLIMDNVAFHKCIEVKEA-----IGN--E-EDK---PLYLPPYS
Mango        71 YELKDIL----R-------S----K------NINRSYLIMDNVAFHKSQSVQEA-----IGT--V-IDK---PLYLPPYS
Kiwi         71 SDLKTKI----V-------D----L------GIGRCILIMDNVPFHRSIDVRNA-----IEE--G-GHT---IMLLPPYS
Whistler     71 DELHDKL----E-------E----K------LITNAILILDNVAFHKSYEVKQK-----IES--Y-GYK---IMYLPPYS
ThonC        70 KQVIQKA----Q-------D----H----Y-KDKKFVLIMDNCSIHKSKEIEQI-----FD-----RFP---CIFTPAYR
Coelocanth   70 TSIINKA----R-------D----F----Y-QQDEFVFVMDNCSVHKSLEMKQI-----YS-----RVP---CIFTPAYR
Merou        70 SSIINKA----Y-------D----Y----Y-KNHKFVLVMDNCSIHKSSQMTSV-----LN-----KIP---CIFTPAYR
Sardine      70 VEVIKKA----Q-------V----F----Y-QEKPFVLILDGCSIHKSSLVNDI-----FE-----EIP---HIYTPAYR
ThonB        70 ISVINKA----Q-------E----H----Y-KNQTFVLIMDNCSIHKSKQIVEI-----FD-----RIP---CIFTPAYR
ThonA        70 ISVINMA----Q-------K----H----Y-KNKKFVLIMNNCSIHKSKQILEI-----FD-----KVP---CIFTPAYR
Baudroie     70 QNLLTYA----N-------Q----H----Y-QEQNFTLVLDNATIHKNQTVKGV-----LD-----RIP---HVFTPPYR
TEC2         69 EKLNSLLKTMIA-------K----K----Q-LMKRTVYVFDNASIHSTEKVVKA-----ITG--M-KMV---CFTIPPYS
TEC1         69 ENLHDKLRTILS-------K----R----Q-LSKRTIYVFDNASIHLTQKVVKC-----VTD--R-KMV---CFTIPPYC
DDE2         70 EKLIQCA----D-------Q----Y----F-NQQNFVIVLDNCSIHKSKLIRQR-----FTQ----NIN---CVFLAPYS
DDE1         70 EKLIISA----T-------E----F----Y-KNQKFLIILDNCPTHQSKMIKQR-----YLH----NIN---YLFLAPYT
Tennessee    70 IQLLESA----T-------Q----F----Y-EKQKFIIIMDNSPIHHSNFMKNK-----IYQ----KCN---VLFLPPYS
DDE3         70 QQIIKQA----R-------Q----F----Y-KEQEFAIVLDNSPIHQSKLIKDF-----LS-----SVT---HLFLPVYC
AnchoisA     71 NELLRLL----Q-------Q----N----S-EENNFCLVLDNASIHRTSQILDQ-----LS-----YVN---YLFLPPYS
AnchoisB     71 NELLKLL----Q-------Q----N----N-EENNFCLVLDNASIHRTQQILNQ-----LS-----FVN---YLFLPPYS
TBE1         72 EQLREKY----P--------------------EQEIILFVDNLSVHKTKETKKS-----YEQ--L-RIT---PVFNVPYS
Mbmar1       75 INLNRALQRKRP-------E----Y----QKRQHRVIFLHDNAPSHTARAVRDT-----LET--L-NWE---VLPHAAYS
Dmmar1       75 INLNRALQRKRP-------E----Y----QKRQHRVIFLHDNAPSHTARAVRDT-----LET--L-NWE---VLPHAAYS
Botmar1      75 IDLNQALREKRP-------E----Y----QKRQHKVILLHDNAPSHTAKPVKET-----IEA--F-SWE---IPSHAAYS
Momar1       75 IDLNRAVKEKRP-------N----W----DQVRNRVILLHDNAQCHTSKPTQET-----LSA--L-NWE---VLTHPRYS
Sinvmar1     75 YQLSDVLMQKRS-------S----V----VNNRCKVILLHDNARPHIAKSVKQV-----LEX--L-AWE---VLPHPAYS
Funmar       75 QVMMEKLAAKQP-------R----L----I-NRSRPLLLHDNARPHTAQQTATK-----LGE--L-QLE---CLRHPPYS
BmMLE        76 QTMKEELAAKQP-------R----L----V-NRSRPLLLHDNARPHTAQQTTTK-----LDE--L-QLA---CLRHPPYS
Planmar8     75 DDMMEKLAIKQA-------K----M----F-NRLTPILLYDNARPHSAKNTVAK-----RQQ--L-GLE---TLRHPPYS
Avmar1       75 DRVAEKLK---------------------G-KQDRVYFLHDNARPHVAKSTREK-----LLK--L-GWI---TIPHPPYS
Cemar2       75 DRVAEKTN---------------------G-KYEKLYFLHDNARPHVAKKTFQK-----LQD--L-GWT---VLPHPPYS
Cbmar1       76 RNLKKAVDR----------S----A----L-KDKKVYYQHDNARPHVSKQVKQE-----LMG--Y-GWN---VLPHPPYS
Mcmar1       74 HRVQQQLRRPPY-------T----V----W-ARKGILFQQDGARPHVSAVTRKK-----IED--L-GWD---ILEHSPYS
Ccmar1       75 VELKKAIDEKRP-------I----F----A-NRKGVLFHYDNARPHVAKPTLAK-----LKE--M-NWE---IMPHSPYS
Acmar1       69 DKLNNSIKQKRP-------E----L----I-NRKGVVFHHDNAKAHMSLMTRQK-----LLQ--L-GWE---VLPHPPYS
Ammar1       75 TKLNNAVEEKRP-------E----L----T-NRKGVVFHHDNARPHTSLVTRQK-----LLE--L-GWD---VLPHPPYS
Famar1       75 TKLNNAVEEKRA-------E----L----T-NRKGVVFHHDNARPHTSLVTRQK-----LLE--L-GWD---VLPHPPYS
Ccmar2       75 TKLNNAVEEKRP-------E----L----T-NRKSIVFHHDNARPHTSLATRQK-----LLE--L-GWD---VLPHPPYS
Camar1       75 DKLNAAINEKRP-------E----L----I-NRKGVIFHQDNARPHTSLMTRQK-----LRE--L-GWE---VLMHPPYS
Himar1       78 ERLKVEIAAKWP-------H----M----K--KKKVLFHQDNAPCHKSLRTMAK-----IHE--L-GFE---LLPHPPYS
Bytmar1      78 GRLRDSIRQKRR-------G----K----L-TRGVLLLLHDNAPVHKAHHAQAA-----LRD--C-GFE---QFNHPSYS
Hsmar2       78 RKLAKALAEKRP-------G----K----L--HQRVLLHHDNAPAHSSHQTRAI-----LRE--F-RWE---IIRHPPYS
Tvmar1       75 TQIINSDE-FEK-------S----K----Q-QKQKFAIHFDNSRVHKSHKVMNY-----LVE--N-NVK---VVPNPIYS
Psmar1B     107 DKVLPAIREKWP-------R----D----E-FESTIFIQQDNARTHINHDDPLFREAATKDG--F-DIR---LMCQPANS
Soymar1     107 EKVLPATKEVWP-------R----D----E-LGSTIFIQQDNARTHINPDDPEFVQAATQDG--F-DIR---LMCQPPNS
Vulmar1     107 QNVLPSIMSKWP-------A---------G-MSKDIFIQQDNARPHIQGNDEAFKAAATQNE--F-NIM---LIQQPPQS
Aee37E1      81 QKRFLPF---IR-------A-----------HDRPVMLWPDLASCHYSKTVIEW-----YAT--N-GVSVIPKDLNPPNC
Aea37E1      81 QKRILPF---IR-------A-----------HDRPVMLWPDLASCHYSKTVIEW-----YAT--N-GVSVIPKDLNPPNC
Aet37E1      76 QKRILPF---IR-------S-----------HDHPVMFWPDLASCHYSKVVQEW-----YAE--K-GVQFVPKHLNPPKC
Ag37E1       81 QKRILPF---IR-------S-----------HDHPVMFWPDLASCHYSKVVREW-----YAE--K-GVLFVPKNLNPPNC
CbmaT5       82 MDVLRPW---VT-------S----H----F-GQQPFILQQDWAPSHGSKSTKAV-----LDA--H-FPGYWGKDMWPASS
CbmaT4       82 MDNLLPW---VT-------Q----H----F-AGGPFILQQDWAPSHGSRSTLAV-----LEA--H-FPGFLDKNLWPASS
CemaT1       76 KTELMPW---VK-------K----H----F-KKTKWTFQQDGAPAHKHKNVQAW-----CES--N-FPDFIAFNQWPPSS
CbmaT1       82 EKHVLPW---AQ-------N----H----F-GQKKYIFQQDGAPAHTAKMAQQW-----CKD--H-FPAFIPKDEWPASS
CemaT3       82 KNHVLPW---TK-------S----H----F-GNQVWTFQQDSAPAHKAKIVHAW-----CTA--N-FPRFITSSEWPASS
CbmaT3       74 KGYVHPW---AN-------F----N----F-ENHVWTIQRDSAPAHKTKIIPCT-----VSF-----SIFSMTCEWSPSS
Bmmar6       82 TNLVEPV---SH-------T----M----F-NNRHWVFQQDSAPAHRAKSTQDW-----LAA--R-EIDFIRHEDWPSSS
Bmmar1       82 EKVVKPL---NN-------T----M----F-NNQEWSFQQDSAPGHKARSTQSW-----LET--N-VSDFIRAEDWPSSS
MdmaT1a      83 KGVLKPW---AR-------K----H----F-GRRPWTFQQDSAPSHLKLLQKEV-------------PRFISSAQWPPKS
Bari1        75 NNHAFTS---GN-------R----L----F-PTTEWILQQDNAPCHKGRIPTKF-----LND--L-NLA---VLPWPPQS
Maya         77 SDHVHPF---MT-------T----M----Y-PSSDGYFQQDNAPCHKARIISNW-----FLEHDN-EYT---VLKWPPQS
Tc3          74 ELELSKY---LR-------H---------Y-SRKDFRFQQDNATIHVSNSTRDY-----FKL--K-KIN---LLDWPARS
S            77 KTNLKAS---AE-------K----FGLFSN-NKPNFKFYQDNDPKHKEYNVRNW-----LLY--N-CGK---VIDTPPQS
Paris        77 KNELSRS---AIKFGFVDPQ----N----P-SKQRYKLYQDNDPKHKSFLCRTW-----LLY--N-CSK---VIDTPAQS
Quetzal      77 KQNLGPS---LE-------K----L----G-MSQDYWFQQDNDPKHTAFNSRLF-----LLY--N-TPH---QLKSPPQS
Tcb2         77 ENTMRPW---AR-------S---------T-VGRAFVFQQDNDPKHTSKHIKEW-----FRR--R-HVD---LLDWPSQS
Tc1          77 ETTMRPW---AL-------Q---------N-VGRGFVFQQDNDPKHTSLHVRSW-----FQR--R-HVH---LLDWPSQS
Tcb1         77 ETVIWPF---VR-------S---------T-ARRGFIFQQDNDPKHKSKLLTKW-----FRD--N-NVP---LLMWPSLS
Minos        76 QDSLLPS---IP-------K----L----L-DCGEFTFQQDGASSHTAKRTKNW-----LQY--N-QME---VLDWPSNS
Impala       77 -SFILPW----------------------F-LQSGDIFMHDNASVHTARIVKAL-----LEE--L-GVD---LMTWPPYS
```

FIGURE S5 3/4

```
                             *
IS630Sd      117 -PWLNPIER-LWLSLHETITR
IS630Ss      117 -PWVNHVER-LWQALHDTITR
Tigger1      148 TSILQPMDQGVISTFKSYYLR
pogoDm       134 TALLQPLDQGIIHSFKLEYRR
Fot1         153 SHVLQPLDLGCFSSLKAAYRT
Flipper      150 SHVLQPPDLSIFSPLKKEYRY
Tan1         150 SHLLQPLDVGCFSVLKRTYGG
Parker       119 -PFLNPIEN-MFSKWKNLVKR
Mango        119 -PFLNPIEN-MFSKWKNYVKR
Kiwi         119 -PFFNPIEN-LFSKWKSLVKR
Whistler     119 -PFLNPIEN-MFAQWKQITKR
ThonC        117 -PEFNAIEH-MFGWLKRGLVA
Coelocanth   117 -PEFNAIEH-MFGWIKRKLVI
Merou        117 -PEFNAIEH-MFGWIKRKLVI
Sardine      117 -PEFNAIEH-MFGWIKRKIVS
ThonB        117 -PEFNAIEH-MFGWLKRGLVV
ThonA        117 -PEFNAIEH-MFGWLKRGLVA
Baudroie     117 -PDLNPIEH-MFSWLKQYLVK
TEC2         122 -PELNKIEH-TFGTLKRNLSR
TEC1         122 -PELNKVEH-TFGLLKNNLSK
DDE2         118 -PELNPIEK-VWKLLKGHVSK
DDE1         118 -PELNPIEK-IWKLLKEQIYK
Tennessee    118 -PELNSIEG-VWNNLKQKIYK
DDE3         117 -PEFNPIER-VWSQLKRMANN
AnchoisA     118 -PQLNCIEK-LWGVAKQKLSK
AnchoisB     118 -PQLNCIEM-LWGIAKRQLSK
TBE1         117 -PQFNGIEF-YWGILKGHYKK
Mbmar1       129 -PDLAPSDYHLFASMGHALAE
Dmmar1       129 -PDLAPSDYHLFASMGHALAE
Botmar1      129 -PDLAPSDYYLFASMGHALSD
Momar1       129 -PDLAPSDYYLFASMSHALNN
Sinvmar1     129 -PDLAPSDYHLFRSMQLTDAH
Funmar       128 -PDLAPTDYHFFRNLDNFLQG
BmMLE        129 --DLAPIDYHFFLNLDNFLQK
Planmar8     128 -PDLAPTDYHFFQSLDNFLSG
Avmar1       122 -PDLAPTDYHLFRSLSNDLRD
Cemar2       122 -PDLAPTDYHLFLSLSDYMRD
Cbmar1       126 -PDLAPSDYWLFGDMTRAFEG
Mcmar1       127 -PDLAPSDY-LFSPLKDFLRG
Ccmar1       128 -PDIAPSDYHLFRSLQNNLNG
Acmar1       122 -PDLAPSDYHLFRSLQNSLND
Ammar1       128 -PDLAPSDYFLFRSLQNSLNG
Famar1       128 -PDLAPSDYFLFRSLQNSLNG
Ccmar2       128 -PDLAPSDYFLFRSLQNSLNG
Camar1       128 -PDLAPSDYHLFRSLQNSLNG
Himar1       130 -PDLAPSDFFLFSDLKRMLAG
Bytmar1      131 -PDLAPNDYFLFRQLKSSLRG
Hsmar2       130 -PDLAPSDFFLFPNLKKSLKG
Tvmar1       127 -PDIAPSDFYLFGTLKKRAEG
Psmar1B      165 -PDLNILDLGFFSAIQSLQYK
Soymar1      165 -PDFNVLDLGFFSAIQSLHYK
Vulmar1      164 -PDLNVLDLGFFRAIQALMYQ
Aee37E1      132 -PQFRPMEK-YWVITKRRLKA
Aea37E1      132 -PQFRPIEK-YWAITKRRLKA
Aet37E1      127 -PQFRPIEK-YWAIMKRMLNA
Ag37E1       132 -PQFRPIEK-YWAIMKRRLKA
CbmaT5       135 -PDLNPLDFSVWGYLEEKVMA
CbmaT4       135 -PDLNPMDFSVWGMLEGKIAG
CemaT1       129 -PDLNPMDYSVWSVLEAKACS
CbmaT1       135 -PDLNPLDYSVWGVLQNKVCA
CemaT3       135 -PDLNPLDYSIWGVLQSKVQE
CbmaT3       125 -PEMNLLDYSAYGIFQSRVQA
Bmmar6       135 -PDLNPLDYKIWQHLEEKACS
Bmmar1       135 -PDLNPLDYDLWSVLESTACS
MdmaT1a      131 -PDTNPLDYCVWSILESKIGT
Bari1        125 -PDLNIIEN-VWAFIKNQRTI
Maya         129 -PDLNPIEH-LWDVVEWELCA
Tc3          123 -PDLNPIEN-LWGILVRIVYA
S            131 -PDLNPIEN-LWAYLKKKVAK
Paris        134 -PDLNPIEN-LWAFLKKRVGK
Quetzal      127 -PDLNPIEH-AWELLERKIRQ
Tcb2         126 -PDLNPIEH-LWEHVERHVRG
Tc1          126 -PDLNPIEH-LWEELERRLGG
Tcb1         126 -PDLNATEN-LWERLKHQVKG
Minos        126 -PDLSPIEN-IWWLMKNQLRN
Impala       122 -PDLNPIEN-LWALMKAEIYR
```

FIGURE S5 4/4

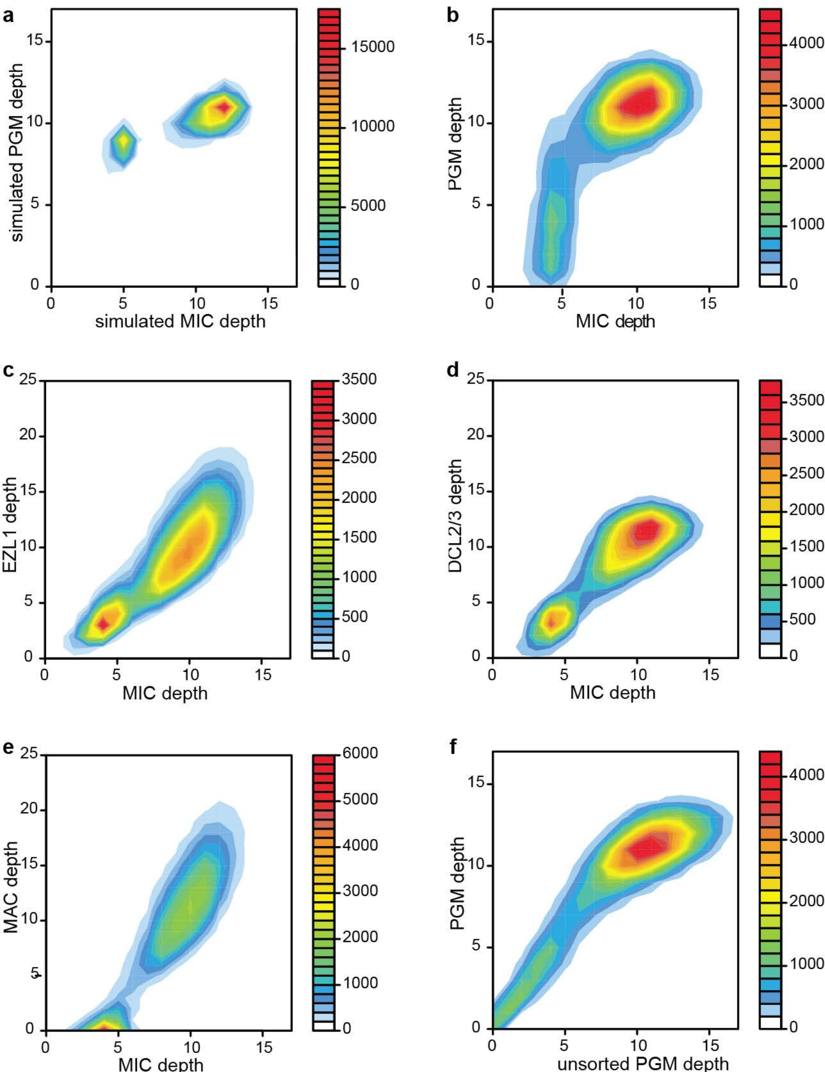
a
simulated PGM depth
simulated MIC depth
0
5
10
15
5000
10000
15000
b
PGM depth
MIC depth
1000
2000
3000
4000
c
EZL1 depth
MIC depth
20
25
500
1000
1500
2000
2500
3000
3500
d
DCL2/3 depth
MIC depth
e
MAC depth
MIC depth
6000
5000
4000
f
PGM depth
unsorted PGM depth

FIGURE S6

FIGURE S7

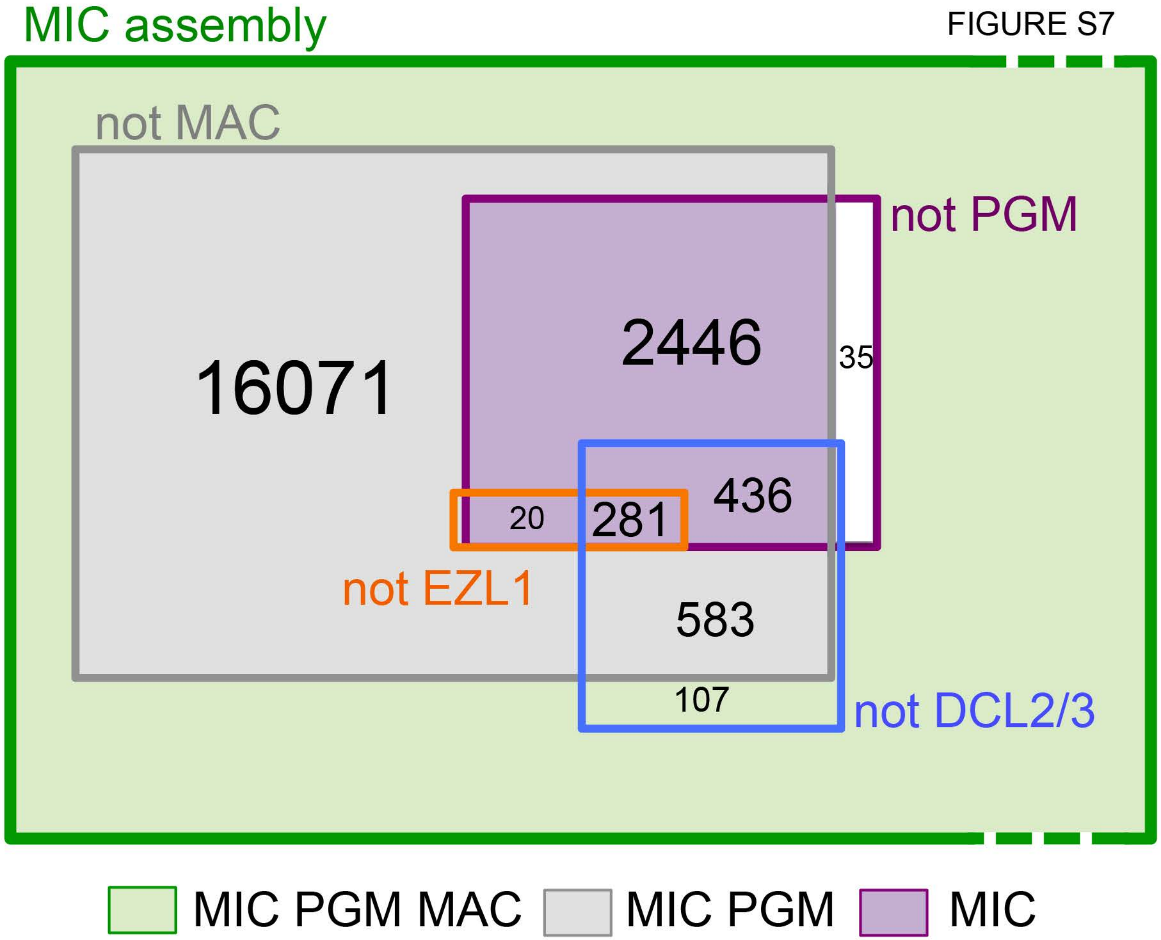
MIC assembly
not MAC
not PGM
16071
2446
35
436
20
281
583
107
not EZL1
not DCL2/3
MIC PGM MAC
MIC PGM
MIC
