# Supplementary material for: Flow cytometry sorting of nuclei enables the first global characterization of Paramecium germline DNA and transposable elements
Source: BMC Genomics. 2017 Apr 26;18:327. doi: 10.1186/s12864-017-3713-7 (PMC5405496; doi:10.1186/s12864-017-3713-7)
Supplement: Supplementary file 2 — This word file contains the following supplementary tables: Tables S1, S2, S5, S6. (DOCX 110 kb) [file 12864_2017_3713_MOESM2_ESM.docx]

**Table S1. DNAseq datasets used in this study.**

| **sample name** | **accession number** | **number of reads (M)** | **read length (nt)** | **percent of reads mapped on contaminants*** | **percent of reads mapped on the MAC** | **percent of reads mapped on the MIC** | **DESeq2 biological replicates** |
| --- | --- | --- | --- | --- | --- | --- | --- |
| MAC | ERA137420 | 87.6 | 74 | 0.4 | 96 | 98 | MAC r1 |
| MAC | SRA063307 | 68.5 | 74 | 4.6 | 94 | 95 | MAC r2 |
| MAC | ERX466735 | 106.1 | 76 | 0.3 | 98 | 99 | MAC r3 |
| MIC | SAMN05323659 | 104.9 | 101 | 8.2 | 89 | 96 |  |
| MIC (flow cytometry) | SAMN05323660 | 100.7 | 101 | 0.2 | 85 | 99 | MIC |
| MIC (Preer) | ERX114956 | 81.8 | 101 | 27.7 | 36 | 41 |  |
| PGM | ERA137444 | 130.2 | 108 | 0.4 | 88 | 97 | PGM r1 |
| PGM (flow cytometry) | SAMN05323661 | 123.2 | 101 | 0.2 | 87 | 99 | PGM r2 |
| DCL2/3 | SRX387766 | 112.4 | 100 | 0.4 | 88 | 99 | DCL2/3 r1 |
| DCL2/3 | ERX466736 | 107.8 | 101 | 0.2 | 93 | 99 | DCL2/3 r2 |
| EZL1 | ERX466733 | 113.3 | 74 | 0.6 | 87 | 97 | EZL1 r1 |
| EZL1 | ERX466734 | 102.5 | 74 | 0.2 | 88 | 99 | EZL1 r2 |

To evaluate contamination, the reads were mapped on reference genomes of known contaminants (*Klebsiella*, *E. coli*, *Pseudomonas*, *Pseudoalteromonas*, *Chryseobacterium*). The filtered reads were then mapped on the MAC or MIC references. More than 95% of reads in control (“MAC”) samples map on a MAC or MIC reference, indicating a maximum of 5% contamination. Between 87-88% of reads map on the MAC and > 97% on the MIC upon *PGM* RNAi with or without sorting (<1% contamination). In the previously published MIC DNA 10, 36 and 41% of reads map respectively on the MAC and MIC references, indicating at least ~60% contamination. MIC DNA (MIC) displayed a contamination of only 8%, demonstrating the improvement of our fractionation procedure. Sorting of the MIC by flow cytometry further decreased the percentage of reads mapping on known contaminants from 8% (before sorting) to 0.2% (after sorting).

**Table S2. Assembly statistics.**

|  |  |  |  |  | **Contig length > 2 kb** | | |
| --- | --- | --- | --- | --- | --- | --- | --- |
| **assembly name** | **file** | **complexity** | **N50** | **number** | **complexity** | **N50** | **number** |
| MAC | ptetraurelia_mac_51.fa | 72102941 | 413026 | 697 |  |  |  |
| PGM | contigs_ABK_COSP_best_k51_no_scaf.fa | 96123393 | 28887 | 23100 | 88689762 | 31908 | 5224 |
| PGM (flow cytometry) | ptetraurelia_PGM_FACS.fa | 95778591 | 55929 | 18728 | 89297215 | 61564 | 3724 |
| MIC | ptetraurelia_mic2.fa | 98489268 | 37181 | 26186 | 90219748 | 42552 | 4492 |

Note that only the MAC assembly consists of scaffolds [1]. The other references assemblies used are contigs produced with Velvet, from paired-end Illumina reads of short-insert libraries (see Methods). The PGM contigs were previously used to characterize IESs [2].

Litterature cited :

1. Aury J-M, Jaillon O, Duret L, Noel B, Jubin C, Porcel BM, et al. Global trends of whole-genome duplications revealed by the ciliate Paramecium tetraurelia. Nature. 2006;444:171–8.

2. Arnaiz O, Mathy N, Baudry C, Malinsky S, Aury J-M, Wilkes CD, et al. The Paramecium germline genome provides a niche for intragenic parasitic DNA: evolutionary dynamics of internal eliminated sequences. PLoS Genet. 2012;8:e1002984.

**Table S5.** **Non-LTR retrotransposon reverse transcriptase proteins used to construct the phylogenetic tree.**

| **Label** | **Species** | **Clade** | **Accession** | **Note** |
| --- | --- | --- | --- | --- |
| REP1_Tth | *Tetrahymena thermophile* | Ciliate | AY371728 |  |
| REP2_Tth | *Tetrahymena thermophile* | Ciliate | AY371729 |  |
| REP6_Tth | *Tetrahymena thermophile* | Ciliate | AY371731 |  |
| RT283 | *Paramecium tetraurelia* | Ciliate | this article | consensus |
| RT50144 | *Paramecium tetraurelia* | Ciliate | this article | consensus |
| RT41453 | *Paramecium tetraurelia* | Ciliate | this article | consensus |
| RT48784 | *Paramecium tetraurelia* | Ciliate | this article | consensus |
| RT3903 | *Paramecium tetraurelia* | Ciliate | this article | consensus |
| RT4152 | *Paramecium tetraurelia* | Ciliate | this article | consensus |
| RT47195 | *Paramecium tetraurelia* | Ciliate | this article | consensus |
| RT36327 | *Paramecium tetraurelia* | Ciliate | this article | consensus |
| RT25765 | *Paramecium tetraurelia* | Ciliate | this article | consensus |
| RT38354 | *Paramecium tetraurelia* | Ciliate | this article | consensus |
| RT33852 | *Paramecium tetraurelia* | Ciliate | this article | consensus |
| RT31010 | *Paramecium tetraurelia* | Ciliate | this article | consensus |
| RT42890 | *Paramecium tetraurelia* | Ciliate | this article | consensus |
| RTTth1 | *Tetrahymena thermophila* | Ciliate | EAR96184 |  |
| RT48569exp | *Paramecium tetraurelia* | Ciliate | this article | consensus |
| RT48569old | *Paramecium tetraurelia* | Ciliate | this article | consensus |
| RT32989 | *Paramecium tetraurelia* | Ciliate | this article | consensus |
| RT43898 | *Paramecium tetraurelia* | Ciliate | this article | consensus |
| RT49653exp | *Paramecium tetraurelia* | Ciliate | this article | consensus |
| RT49653old | *Paramecium tetraurelia* | Ciliate | this article | consensus |
| RT24068 | *Paramecium tetraurelia* | Ciliate | this article | consensus |
| RT3220 | *Paramecium tetraurelia* | Ciliate | this article | consensus |
| RT48639exp | *Paramecium tetraurelia* | Ciliate | this article | consensus |
| RT48639old | *Paramecium tetraurelia* | Ciliate | this article | consensus |
| RT43773 | *Paramecium tetraurelia* | Ciliate | this article | consensus |
| RT43775 | *Paramecium tetraurelia* | Ciliate | this article | consensus |
| RT12275 | *Paramecium tetraurelia* | Ciliate | this article | consensus |
| RT33971 | *Paramecium tetraurelia* | Ciliate | this article | consensus |
| RT23387 | *Paramecium tetraurelia* | Ciliate | this article | consensus |
| RT4057 | *Paramecium tetraurelia* | Ciliate | this article | consensus |
| RT46222 | *Paramecium tetraurelia* | Ciliate | this article | consensus |
| RT46728 | *Paramecium tetraurelia* | Ciliate | this article | consensus |
| RT42365 | *Paramecium tetraurelia* | Ciliate | this article | consensus |
| RT50095 | *Paramecium tetraurelia* | Ciliate | this article | consensus |
| RT10058 | *Paramecium tetraurelia* | Ciliate | this article | consensus |
| RT13985 | *Paramecium tetraurelia* | Ciliate | this article | consensus |
| RT46341 | *Paramecium tetraurelia* | Ciliate | this article | consensus |
| RT21873 | *Paramecium tetraurelia* | Ciliate | this article | consensus |
| CR1_T1-ORF2p | *Anopheles gambiae* | CR1 | AAA29367 |  |
| CR1_turtle | *Acanthochelys spixii* | CR1 | BAA88337 |  |
| CRE1 | *Crithidia fasciculate* | CRE | AAA75435 |  |
| CRE2 | *Crithidia fasciculata* | CRE | AAB40036 |  |
| SLACS_2p | *Trypanosoma brucei* | CRE | CAA34931 |  |
| CZAR | *Trypanosoma cruzi* | CRE | AAA30239 |  |
| I_DM_2p | *Drosophila melanogaster* | I | AAA70222 |  |
| I_Dt_2p | *Drosophila teissieri* | I | AAA74495 |  |
| Jockey_AMY | *Bombyx mori* | Jockey | AAA17752 |  |
| Jockey_JuanA | *Aedes aegypti* | Jockey | AAA29354 |  |
| Jockey_TART | *Drosophila melanogaster* | Jockey | AAC46494 |  |
| Jockey_DOC_2p | *Drosophila melanogaster* | Jockey | CAA35587 |  |
| L1_Arabidopsis | *Arabisopsis thaliana* | L1 | AAC13599 |  |
| L1_Xenope | *Xenopus laevis* | L1 | AAA49976 |  |
| L1_Medaka | *Oryzias latipes* | L1 | AAD02928 |  |
| L1_rat | *Rattus norvegicus* | L1 | AAB41224 |  |
| L1_Human | *Homo sapiens* | L1 | AAC51279 |  |
| L1_chlorella | *Chlorella vulgaris* | L1 | BAA25763 |  |
| LOA_Lian-Aa1_1p | *Aedes aegypti* | Loa | AAB65093 |  |
| LOA_2p | *Drosophila silvestris* | Loa | X60177 |  |
| R1-2_DYa_1p | *Drosophila Yakoba* | R1 | R1-2_DYa | RepBase |
| R1_TRAS1 | *Bombyx mori* | R1 | BAA07467 |  |
| R2_Earwig | *Forficula auricularia* | R2 | AAC34906 |  |
| R2_DMer | *Drosophila mercatorum* | R2 | AAB94032 |  |
| R4_Ascaris | *Ascaris lumbricoides* | R4 | AAA97394 |  |
| R4_Ancylostoma | *Ancylostoma duodenale* | R4 | KIH46084 |  |
| R4_Oesophagostomum | *Oesophagostomum dentatum* | R4 | KHJ80270 |  |
| R4_Pediculus | *Pediculus humanus corporis* | R4 | EEB19129 |  |
| RTE-1_1p_Cel | *Caenorhabditis elegans* | RTE | AAC72298 |  |
| RTE_JAM1_1p | *Aedes aegypti* | RTE | Z86117 |  |
| RTE_BovB_1p | *Bos taurus* | RTE | BovB | RepBase |
| RTBmo4 | *Bombyx mori* | RTE | ADI61815 |  |
| RTBmo7 | *Bombyx mori* | RTE | ADI61820 |  |
| RTBmo3 | *Bombyx mori* | RTE | ADI61814 |  |
| RTBmo5 | *Bombyx mori* | RTE | ADI61816 |  |
| RTBmo6 | *Bombyx mori* | RTE | ADI61821 |  |
| Tad1_2p_Neurospora | *Neurospora crassa* | Tad | AAA21781 |  |
| Tad1_Cgt1 | *Colletotrichum gloeosporioides* | Tad | AAA85636 |  |

The accession numbers are from GenBank unless otherwise noted. The RT from *Paramecium* are manually curated consensus sequences, available in Additional File 4.

**Table S6. List of IS630-Tc1-mariner transposons used for phylogeny**

| **Label** | **Catalytic triad** | **Species** | **Accession** |
| --- | --- | --- | --- |
| IS630Ss | DD35E | *Shigella sonnei* | X05955 |
| IS630Sd | DD35E | *Salmonella dublin* | A43586 |
| TBE1 | DD34E | *Oxytricha fallax* | AAA18578 |
| TEC2 | DD34E | *Euplotes crassus* | AAA91339 |
| TEC1 | DD34E | *Euplotes crassus* | AAA62601 |
| Tennessee | DD33E | *Paramecium primaurelia* | consensus |
| AnchoisA | DD32E | *Paramecium tetraurelia* | consensus |
| AnchoisB | DD32E | *Paramecium tetraurelia* | consensus |
| DDE1 | DD33E | *Paramecium tetraurelia* | consensus |
| DDE2 | DD33E | *Paramecium tetraurelia* | consensus |
| DDE3 | DD32E | *Paramecium tetraurelia* | consensus |
| Sardine | DD32E | *Paramecium tetraurelia* | consensus |
| ThonA | DD32E | *Paramecium tetraurelia* | consensus |
| ThonB | DD32E | *Paramecium tetraurelia* | consensus |
| ThonC | DD32E | *Paramecium tetraurelia* | consensus |
| Merou | DD32E | *Paramecium tetraurelia* | consensus |
| Coelocanth | DD32E | *Paramecium tetraurelia* | consensus |
| Baudroie | DD32E | *Paramecium tetraurelia* | consensus |
| pogoDm | DD30D | *D. melanogaster* | S20478 |
| Tigger1 | DD32D | *H. sapiens* | U49973 |
| Fot1 | DD35D | *Fusarium oxysporum* | S20466 |
| Tan1 | DD35D | *Aspergillus niger* | U58946 |
| Flipper | DD35D | *Botryotinia fuckeliana* | U74294 |
| Famar1 | DD34D | *Forficula auricularia* | AY155492 |
| Ammar1 | DD34D | *Apis mellifera* | AY155490 |
| Ccmar2 | DD34D | *Ceratitis capitata* | AY155493 |
| Camar1 | DD34D | *Chymomyza amoena* | AY155491 |
| Acmar1 | DD34D | *Apis cerana* | AB081476 |
| Ccmar1 | DD34D | *Ceratitis capitata* | U40493 |
| Dmmar1 | DD34D | *Drosophila mauritiana* | X78906 |
| Mbmar1 | DD34D | *Mamestra brassicae* | AF465247 |
| Botmar1 | DD34D | *Bombus terrestris* | consensus |
| Momar1 | DD34D | *Metaseiulus occidentalis* | U15665 |
| Sinvmar1 | DD34D | *Solenopsis invicta* | AF518169 |
| BmMLE | DD34D | *Bombyx mori* | D88671 |
| Funmar1 | DD34D | *Fungia sp.* | AB055188 |
| Planmar8 | DD34D | *Girardia tigrina* | X80895 |
| Cemar2 | DD34D | *Caenorhabditis elegans* | Z81500 |
| Avmar1 | DD34D | *Adineta vaga* | DQ138246 |
| Mcmar1 | DD34D | *Meloidogyne chitwoodi* | AJ437557 |
| Cbmar1 | DD34D | *Caenorhabditis briggsae* | consensus |
| Himar1 | DD34D | *Haematobia irritans* | U11642 |
| Hsmar2 | DD34D | *Homo sapiens* | U49974 |
| Bytmar1 | DD34D | *Bythograea thermydron* | AJ507219 |
| Tvmar1 | DD34D | *Trichomonas vaginalis* | AY282463 |
| CbmaT4 | DD37D | *Caenorhabditis briggsae* | AC084524 (10014-11315) |
| CbmaT5 | DD37D | *Caenorhabditis briggsae* | AC084578 (9469-10771) |
| Bmmar1 | DD37D | *Bombyx mori* | U47917 |
| Bmmar6 | DD37D | *Bombyx mori* | AF461149 |
| CemaT1 | DD37D | *Caenorhabditis elegans* | U41268 |
| MdmaT1a | DD37D | *Musca domestica* | AF315724 |
| CbmaT1 | DD37D | *Caenorhabditis briggsae* | AC084526 (30069-31371) |
| CemaT3 | DD37D | *Caenorhabditis elegans* | Z81459 (10,038-10,808) + Z79604 (5-536) |
| CbmaT3 | DD37D | *Caenorhabditis briggsae* | G2014 (19449-20733) |
| Soymar1 | DD39D | *Glycine max* | AF078934 |
| Psmar1B | DD39D | *Pisum sativum* | AY833550 |
| vulmar1 | DD39D | *Beta vulgaris* | AJ556159 |
| Tc1 | DD34E | *Caenorhabditis elegans* | X01005 |
| Tcb2 | DD34E | *Caenorhabditis briggsae* | M64308 |
| Tcb1 | DD34E | *Caenorhabditis briggsae* | X07827 |
| Paris | DD34E | *Drosophila virilis* | U26938 |
| S | DD34E | *Drosophila melanogaster* | U33463 |
| Quetzal | DD34E | *Anopheles albimanus* | L76231 |
| Bari1 | DD34E | *Drosophila melanogaster* | X67681 |
| Minos | DD34E | *Drosophila hydei* | Z29102 |
| Tc3 | DD34E | *Caenorhabditis elegans* | P34257 |
| Impala | DD34E | *Fusarium oxysporum* | AF282722 |
| Maya | DD36E | *Xenopus tropicalis* | AJ810409 |
| Kiwi | DD34E | *Anopheles gambiae* | AAAB01008846 (12859-14738) |
| Whistler | DD34E | *Anopheles gambiae* | AAAB01008849 (597731-600183) |
| Mango | DD34E | *Anopheles gambiae* | AAAB01008879 (621801-624850) |
| Parker | DD34E | *Anopheles gambiae* | AAAB01008960 (17976068-17979727) |
| Ae.atropalpus.ITmD37E1 | DD37E | *Aedes atropalpus* | AF377999 |
| Ae.epactius.ITmD37E1 | DD37E | *Aedes epactius* | AF378000 |
| An.gambiae.ITmD37E1 | DD37E | *Anopheles gambiae* | AF378002 |
| Ae.triseriatus.ITmD37E1 | DD37E | *Aedes triseriatus* | AF378001 |

*Paramecium* consensus sequences, this article. Botmar1 and Cbmar1 consensus sequences, Brillet et al., Genetica. 2007;130:105–20.
